# LRIG1 is a pleiotropic androgen receptor-regulated feedback tumor suppressor in prostate cancer

**DOI:** 10.1038/s41467-019-13532-4

**Published:** 2019-12-02

**Authors:** Qiuhui Li, Bigang Liu, Hsueh-Ping Chao, Yibing Ji, Yue Lu, Rashid Mehmood, Collene Jeter, Taiping Chen, John R. Moore, Wenqian Li, Can Liu, Kiera Rycaj, Amanda Tracz, Jason Kirk, Tammy Calhoun-Davis, Jie Xiong, Qu Deng, Jiaoti Huang, Barbara A. Foster, Abhiram Gokhale, Xin Chen, Dean G. Tang

**Affiliations:** 1 Department of Pharmacology and Therapeutics, Roswell Park Comprehensive Cancer Center, Buffalo, NY 14263 USA; 2 State Key Laboratory Breeding Base of Basic Science of Stomatology (Hubei-MOST) and Key Laboratory for Oral Biomedicine of Ministry of Education (KLOBM), School and Hospital of Stomatology, Wuhan University, 430079 Wuhan, China; 3 Department of Epigenetics and Molecular Carcinogenesis, University of Texas M.D. Anderson Cancer Center, Science Park, Smithville, TX 78957 USA; 4 Department of Pathology, Duke University of School of Medicine, Durham, NC 27710 USA; 5 Department of Oncology, Tongji Hospital, Tongji Medical School, Huazhong University of Science and Technology (HUST), 430030 Wuhan, China; 6 Cancer Stem Cell Institute, Research Center for Translational Medicine, East Hospital, Tongji University School of Medicine, 200120 Shanghai, China

**Keywords:** Cancer, Tumour-suppressor proteins, Urological cancer, Prostate cancer, Cell biology

## Abstract

LRIG1 has been reported to be a tumor suppressor in gastrointestinal tract and epidermis. However, little is known about the expression, regulation and biological functions of LRIG1 in prostate cancer (PCa). We find that LRIG1 is overexpressed in PCa, but its expression correlates with better patient survival. Functional studies reveal strong tumor-suppressive functions of LRIG1 in both AR^+^ and AR^−^ xenograft models, and transgenic expression of LRIG1 inhibits tumor development in Hi-Myc and TRAMP models. LRIG1 also inhibits castration-resistant PCa and exhibits therapeutic efficacy in pre-established tumors. We further show that 1) AR directly transactivates LRIG1 through binding to several AR-binding sites in *LRIG1* locus, and 2) LRIG1 dampens ERBB expression in a cell type-dependent manner and inhibits ERBB2-driven tumor growth. Collectively, our study indicates that LRIG1 represents a pleiotropic AR-regulated feedback tumor suppressor that functions to restrict oncogenic signaling from AR, Myc, ERBBs, and, likely, other oncogenic drivers.

## Abstract (web summary)

LRIG1 is a tumour suppressor in several cancers. Here, the authors show that androgen receptor directly transactivates LRIG1 which then antagonises ERBB signalling and c-Myc expression to inhibit prostate tumorigenesis.

## Introduction

LRIG1 (also called LIG1) was cloned in 1996 from a cDNA that encodes a surface glycoprotein with unknown functions^1^. It was later re-discovered during searches for human homolog(s) of *Drosophila* surface protein Kekkon-1, which is induced by EGF and functions in a feedback loop to dampen the EGF/EGFR signaling^2^. Earlier Northern blotting analysis reveals prominent *LRIG1* mRNA expression in several post-mitotic tissues with slow cellular turnover including brain, heart, and muscle^2^, implicating LRIG1 in enforcing organ dormancy. Consistently, targeted disruption of *Lrig1* gene in mouse results in epidermal hyperplasia resembling psoriasis^3^. Recent RNA-seq analysis in GTEx (Genotype-Tissue Expression) project reveals wide expression of *LRIG1* mRNA across many human tissues including the prostate (Supplementary Fig. 1a).

LRIG1 is a 1093 amino acid (aa) type I transmembrane (TM) protein with a N-terminus (N-ter) signal peptide, 15 leucine-rich repeats (LRR), 3 Ig domains, a TM domain, and a C-ter 278-aa cytoplasmic tail (Supplementary Fig. 1b). A polyclonal antibody directed against the N-ter (aa 1-151) detected LRIG1, in denaturing SDS-PAGE under reducing conditions, at 143 kDa and 134 kDa, the former of which could be cleaved into an N-ter ~110-kDa species and a C-ter 32-kDa species^4^ (Supplementary Fig. 1c). Shortly after *LRIG1* was cloned, it was hypothesized to function as a potential tumor suppressor gene because the genomic region that harbors the gene, 3p14.3, is frequently deleted in human cancers^5^. Subsequent genomic, histological and functional studies have demonstrated downregulation and tumor-inhibitory effects of LRIG1, and correlated LRIG1 to favorable clinical outcomes, in several human cancers including breast, bladder, colon, cervical, and non-small-cell lung cancers and gliomas^6–14^.

In 2004, two groups^15,16^ reported that LRIG1 negatively regulates the ERBB family (including ERBB1/EGFR, ERBB2/HER2/Neu, ERBB3/HER3, and ERBB4/HER4) of the receptor tyrosine kinases (RTKs) by physically associating with the receptors and promoting their degradation^17–21^. For example, Gur et al.^15^ showed that EGF stimulation upregulated LRIG1, which physically associated with all 4 ERBB family members followed by recruitment of E3 ubiquitin ligase c-Cbl to mediate ubiquitylation and degradation of both EGFR and LRIG1. The authors speculated that LRIG1 is evolved in mammals to attenuate the RTK signaling^15^. In addition to ERBBs, LRIG1 also inhibits other RTKs including c-Met^22,23^, IGF-1R^23^, RET^24^, TrkB (neurotrophic receptor tyrosine kinase 2, NRTK2)^25^, and mutant EGFR (EGFRviii)^23,26^ as well as other oncogenic signaling molecules such as TNFα^27^ and Stat3^28^.

Associated with its inhibition of ERBB and other mitogenic signaling, LRIG1 has been evinced to play a critical role in regulating the quiescence and homeostasis of stem cells in the interfollicular epidermis^29–32^ and the gastrointestinal (GI) tract including the small intestine, colon, and stomach^33–38^. Another concept derived from these studies is that LRIG1 expression marks stem/progenitor cells in these tissues. Of significance, ablation of *Lrig1* results in duodenal adenomas and other GI tumors associated with increased expression of ERBB1-3 and some ligands^34,39,40^, providing genetic evidence that LRIG1 functions as a tumor suppressor. LRIG1 also functions as a haplo-insufficient tumor suppressor in gliomas^41^. Finally, lineage tracing studies demonstrate that loss of one allele of tumor suppressor *Apc* in Lrig1^+^ colonic progenitors^42^ and activation of oncogenic β-catenin in Lrig1^+^ epidermal cells^43^ led to formation of colon tumors and trichoadenomas, respectively, suggesting that Lrig1^+^ epithelial stem/progenitor cells can act as a cell-of-origin for tumorigenesis.

Surprisingly, despite the large body of knowledge on LRIG1 in many tissues and tumor systems, little is known, and few papers have been published, about LRIG1 functions and regulation in prostate cancer (PCa). In fact, only 1 study was dedicated to PCa, in which the authors performed immunohistochemical (IHC) analysis of LRIG1 protein expression in cohorts of Swedish and American PCa patients and reported contrasting results on LRIG1 expression in association with Gleason score, tumor stage, and overall survival^44^. Here, we systematically investigate LRIG1 expression, functions, and regulation in PCa. We find that in contrast to its downregulation in some human tumors^11,12,14,41^, LRIG1 is overexpressed in human PCa. Importantly, our results demonstrate that LRIG1 represents an androgen receptor (AR) regulated gene and exhibits tumor-suppressive functions in both xenograft and genetic prostate tumor models.

## Results

### Upregulation of LRIG1 in PCa correlates with good survival

**Fig. 1:**
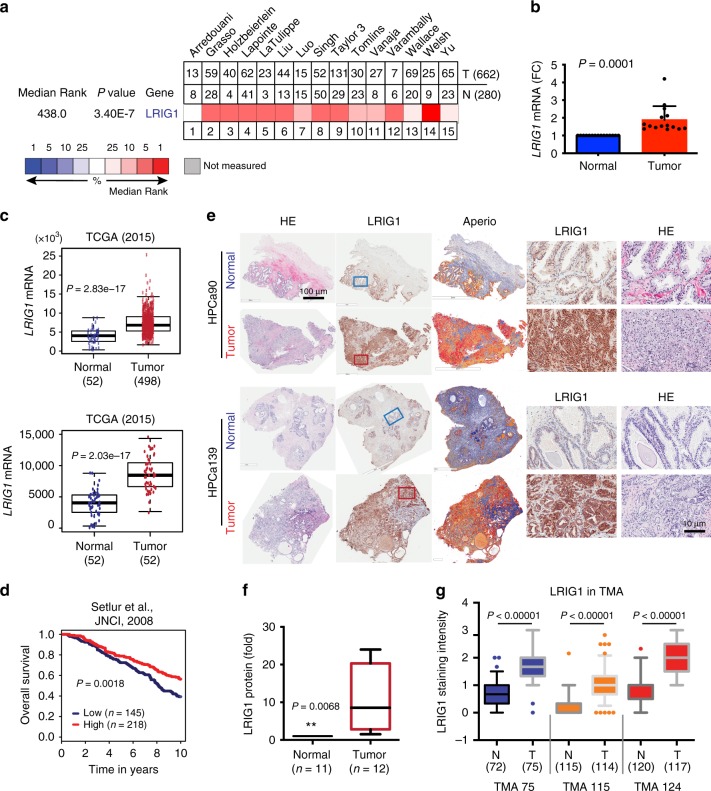
**Overexpression of LRIG1 in PCa and correlation with better patient survival.** **a**–**c** Elevated *LRIG1* mRNA in PCa in Oncomine (**a**, **b**) and in TCGA (**c**). In **b**, the LRIG1 mRNA levels were extracted from tumors (T) in the 15 Oncomine datasets (**a**) and presented as fold changes (FC) after normalizing to the corresponding normal/benign tissues (N). The error bar represents the mean±S.D. In **c**, the center lines represent median values, box edges are 75th and 25th percentiles, and whiskers represent the maximum and minimum values, respectively. **d** High *LRIG1* mRNA levels correlate with better patient overall survival (the Setlur dataset). *P*-value was determined using the Log-Rank test. **e**, **f** Prostate tumors overexpress LRIG1 protein. Shown in e is LRIG1 IHC (using Sigma mAb, 1:100 dilution) in 2 HPCa (i.e., HPCa90 and HPCa139) and matched benign tissues. Enlarged images of boxed regions are shown on the right. Scale bars are indicated in one representative whole-mount (left) and enlarged (right) image, respectively. In **f**, whole-mount slides were scanned by Aperio Scanscope and used to quantify LRIG1 expression levels. The centerline represents the median, box edges are 75th and 25th percentiles, and whiskers represent maximum and minimum values. **g** Increased LRIG1 protein expression in PCa. LRIG1 staining intensity was determined in 3 TMAs that contained both tumors (T) and normal (N) tissues (total numbers indicated below). The centerline represents the median, box edges are 75th and 25th percentiles, and whiskers represent 95% and 5% values, respectively. The *P*-values in **b**, **c**, **f**, and **g** were determined by two-tailed unpaired Student’s *t*-test. *Source data for Fig. 1f, g are provided as a Source Data file.

We first analyzed *LRIG1* mRNA levels in Oncomine PCa database. Among the 15 PCa datasets that had detectable LRIG1, *LRIG1* mRNA was overexpressed in prostate tumors (T) compared to normal (N)/benign prostate tissues (Fig. 1a; Supplementary Figs. 2 and 3a). When *LRIG1* mRNA levels from each microarray dataset were extracted, normalized, and pooled for comparisons, the 662 prostate tumors showed much higher *LRIG1* expression than the 280 normal samples (Fig. 1b). Similarly, PCa samples in TCGA also expressed higher levels of *LRIG1* mRNA than normal tissues (Fig. 1c). Notably, the *LRIG1* mRNA levels positively correlated with PCa patients’ overall survival (OS) (Fig. 1d; Supplementary Fig. 3b).

We then determined LRIG1 protein expression by performing IHC and immunofluorescence (IF) staining using several anti-LRIG1 antibodies (Supplementary Fig. 1c; Supplementary Table 1). IHC studies, combined with Aperio Scanscope-based quantification^45–51^, revealed significantly higher levels of LRIG1 protein in 12 whole-mount (WM) untreated human PCa (HPCa) specimens (Fig. 1e, f; Supplementary Fig. 4a, b; Supplementary Table 2) compared to matched normal/benign tissues. LRIG1 protein in normal prostate tissues was observed mostly at low levels at the plasma membrane of the glandular structures whereas, in contrast, it was detected at significantly elevated levels mostly in the cytoplasm with some membrane localization (Supplementary Fig. 4b). Similar IHC analysis in three tissue microarrays (TMAs) containing 306 prostate tumor and 307 normal prostate tissue cores also revealed increased LRIG1 in PCa (Fig. 1g; Supplementary Fig. 4c–f). Differential LRIG1 protein expression could be readily discerned in matched T/N cores from the same patient (Supplementary Fig. 4d) and, strikingly, in T/N areas of the same TMA core (Supplementary Fig. 4e). Dual IF staining revealed high LRIG1 expression, homogeneously and exclusively, in cancer areas positive for the PCa biomarker AMACR (Alpha-MethylAcyl-CoA Racemase), as illustrated in Supplementary Fig. 4g.

### LRIG1 inhibits AR^−^LRIG1^−/lo^ PCa xenografts

The positive correlation between *LRIG1* mRNA levels and better PCa patient OS suggests a potential tumor-suppressive function of LRIG1 in PCa. We first tested this possibility in xenograft human PCa models that our lab has been utilizing^45–51^ by performing in vitro and in vivo gain- and loss-of-function studies using a suite of new tools we developed (Supplementary Fig. 5). Examination of LRIG1 expression in 11 prostate and PCa cell types revealed an interesting LRIG1 expression pattern associated with AR expression status and levels (Supplementary Fig. 6). For example, cultured LNCaP and xenograft-derived LAPC4 and VCaP cells expressed high levels of AR mRNA and protein and also high, though variable, levels of LRIG1 (Supplementary Fig. 6a–c), which was detected as a major ~143 kD band and minor 110-kD band (Supplementary Fig. 6b), the latter of which likely represented the cleavage fragment of the 143-kD band^4^. IF analysis corroborated LRIG1 expression in these 3 PCa cell types as well as in AR^+^, androgen-dependent (AD) LAPC9 (i.e., LAPC9-AD) cells (Supplementary Fig. 6d). Of note, quantitative analysis using the Wes system (see Methods) revealed that 22Rv1 cells expressed much lower levels of AR and, correspondingly, lower levels of LRIG1, than LNCaP cells (Supplementary Fig. 6c). Other cell types, including RWPE-1, PC3, Du145, IGR^-^1, PPC-1, and LAPC9-AI (i.e., androgen-independent LAPC9 xenograft cells^46,47,49,51^), expressed low/little AR and, correspondingly, barely detectable LRIG1 (Supplementary Fig. 6b, c). Together, these experiments demonstrated that the 5 AR^+^ PCa cell types (LAPC4, VCaP, LNCaP, LAPC9-AD, and 22Rv1) expressed variable levels of endogenous LRIG1 (LAPC4 > VCaP > LNCaP > LAPC9-AD > 22Rv1) that overall correlated with their AR levels whereas 5 AR^−/lo^ PCa cell types (IGR-1, PC3, PPC-1, Du145, and LAPC9-AI) had low to undetectable LRIG1 (Supplementary Figs. 6b, c and 7a; also see Supplementary Fig. 13g, below).

**Fig. 2:**
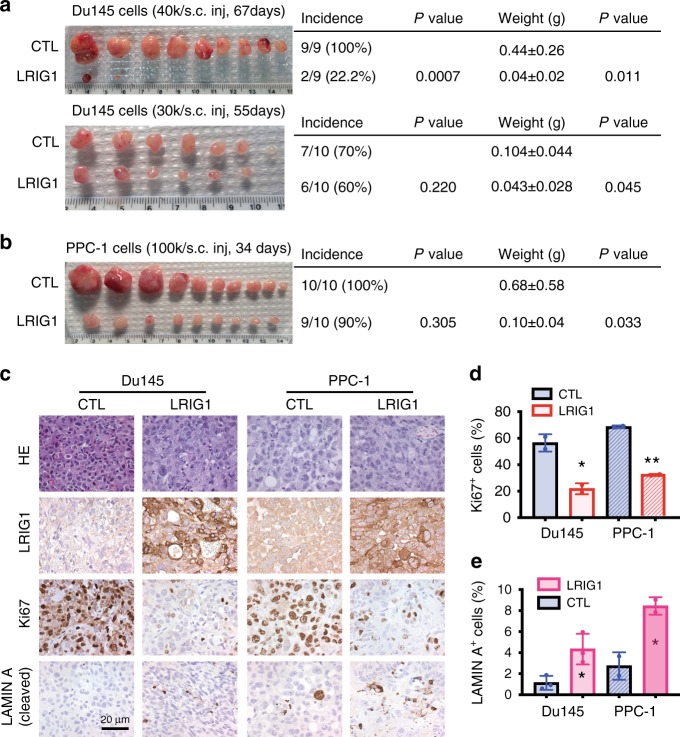
**LRIG1 overexpression inhibits tumor regeneration in AR**^**−/lo**^ **PCa xenografts.** **a**, **b** LRIG1 overexpression inhibits tumor regeneration and growth of AR^-^ Du145 (**a**; two independent experiments shown) and PPC-1 (**b**) cells. PCa cells infected with control (CTL) or LRIG1-expressing lentiviral vectors (MOI 5), and subcutaneously (s.c) injected (cell # indicated) in NOD/SCID mice. Tumors were harvested at the days indicated. Tumor incidence, endpoint tumor weight, and *P* values are presented on the right. The *P*-values for tumor incidence and weight were determined using *χ*^2^ test and Student’s *t*-test, respectively. **c** Representative IHC images of LRIG1 (Sigma mAb, 1:100 dilution), Ki67 (Novus Biological, 1:500 dilution), and cleaved LAMIN A for apoptosis (Cell Signaling, 1:500 dilution) staining in endpoint tumors. In all, 4 μm serial sections were stained for the molecules indicated. Original magnifications were ×400 and a scale bar was indicated on one panel. **d**, **e** Quantification of Ki67^+^ (**d**) and LAMIN A^+^ (**e**) cells in endpoint tumors. Bars, mean ± SEM (by counting a total of 1000–1500 cells from 2 to 3 tumors from each model. *P < 0.05; **P < 0.01 (two-tailed unpaired Student’s *t*-test). *Source data for Fig. 2a, b, d, e are provided as a Source Data file.

Subsequently, we determined how LRIG1 overexpression might influence tumor regeneration and growth in PCa cells that expressed little endogenous LRIG1. Lentiviral-mediated (Supplementary Fig. 5a) LRIG1 overexpression (Supplementary Fig. 7a, b) in AR^−^ PCa cells, Du145 and PPC-1, inhibited tumor incidence and/or tumor growth (weight) in NOD/SCID mice (Fig. 2a, b), which was associated with decreased cell proliferation (Ki-67^+^ cells) and slightly increased cell death (cleaved lamin A^+^ cells) in endpoint tumors (Fig. 2c, d). In vitro, LRIG1 overexpression suppressed 2D clonal and 3D clonogenic and sphere-forming capacities as well as proliferation in both Du145 (Supplementary Fig. 7c–e) and PPC-1 (Supplementary Fig. 7f–i) cells. Stable LRIG1 expression in Du145 cells using a retroviral LRIG1 expression vector^15^ (Supplementary Fig. 5b) similarly inhibited in vitro pro-tumorigenic properties as well as in vivo tumor growth (Supplementary Fig. 8).

### Knocking down endogenous LRIG1 inhibits AR^+^LRIG1^+^ PCa

**Fig. 3:**
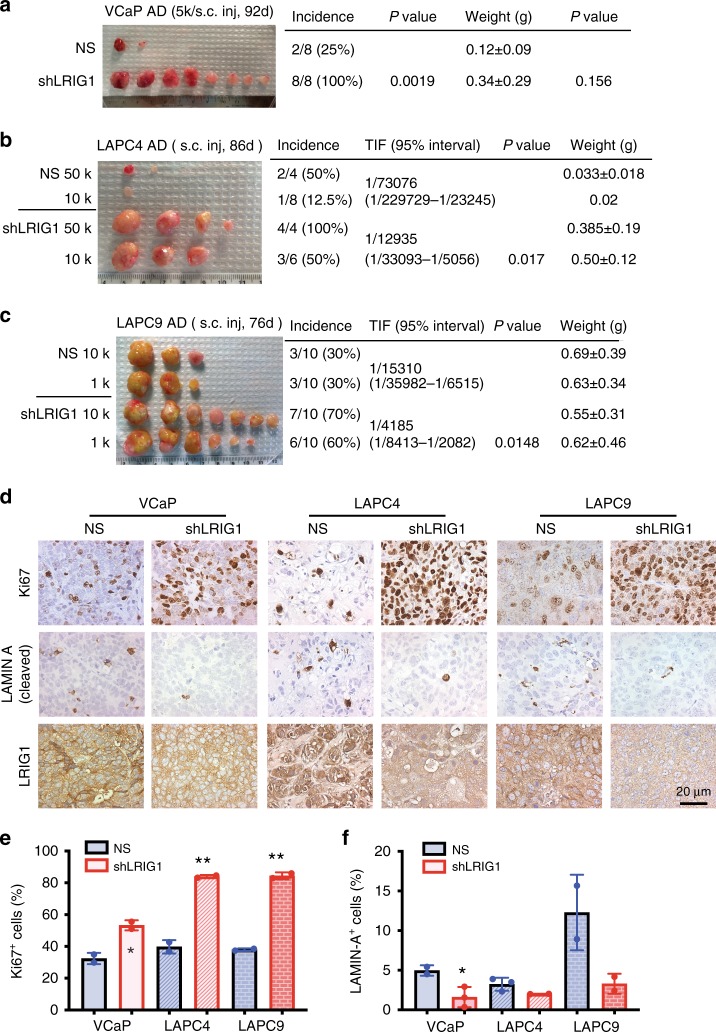
**Knocking down endogenous LRIG1 promotes tumor regeneration in AR**^**+**^ **PCa xenografts.** **a** Knocking down endogenous LRIG1 in AR^+^ VCaP cells promotes tumor regeneration and growth. VCaP cells purified from maintenance tumors were infected with non-silencing (NS) or shLRIG1 lentivectors (MOI 10; 12 h) and 5000 (5k) cells were subcutaneously (s.c) injected in male NSG mice. Tumors were harvested 92 days after implantations. Shown on the right are tumor incidence and weight and corresponding *P* values (*χ*^2^ test for incidence and Student’s *t*-test for weight; note that the *P* value for weight comparison was not statistically significant due to low tumor incidence in the NS group). **b**, **c** Limiting dilution tumor regeneration assays in LAPC4 (**b**) and LAPC9 (**c**) AD cells upon LRIG1 knockdown. Cells freshly purified from maintenance AD xenografts were infected as in A and implanted at two cell doses in male NOD/SCID mice. Tumors were harvested at the indicated time points. Tumor-initiating frequency (TIF) was calculated and compared (*χ*^2^ test). **d** Representative IHC images of LRIG1 (Sigma mAb), Ki67, and cleaved LAMIN A staining (see Fig. 2c for dilutions of these three antibodies) in endpoint VCaP, LAPC4, and LAPC9 xenograft tumors. Tumors derived from LRIG1 KD cells contained more proliferating (Ki67^+^) cells than control tumors. Original magnifications were ×400 and a scale bar was indicated on one panel. **e**, **f** Quantification of Ki67^+^ (**e**) and cleaved LAMIN A^+^ (**f**) cells in endpoint tumors indicated. Bars represent mean ± SEM by counting 1000–1500 cells from 2 to 3 individual tumors in each model. *P < 0.05; ***P < 0.001 (two-tailed unpaired Student’s *t*-test). *Source data for Fig. 3a, b, c, e, f are provided as a Source Data file.

In contrast to above results, knocking down endogenous LRIG1 using pGIPZ-shLRIG1 (Supplementary Fig. 5c) in 3 AR^+^, AD PCa cells, VCaP, LAPC4, and LAPC9, significantly promoted tumor regeneration (Fig. 3a–c). Specifically, LRIG1 knockdown in VCaP cells resulted in increased tumor incidence (*P* = 0.0019; *χ*^2^ test) and larger tumors (note that tumor weight comparison was not statistically significant due to the small number of regenerated tumors in the NS group) (Fig. 3a). Limiting-dilution tumor regeneration assays^45–47,49–51^ in LAPC4 and LAPC9 AD models revealed tumor-promoting effects upon LRIG1 knockdown, as evidenced by increased tumor incidence (Fig. 3b, c). The enhanced tumor regeneration in all 3 AR^+^ xenograft models upon LRIG1 knockdown was associated with increased cell proliferation and slightly reduced cell death (Fig. 3d–f). Consistently, lentiviral-mediated LRIG1 knockdown in vitro enhanced 2D clonal, 3D clonogenic and proliferative capacities in AR^+^ LNCaP, VCaP, and LAPC9 cells (Supplementary Fig. 9a–g). Similarly, siRNA-mediated LRIG1 knockdown (see Methods) promoted clonal growth and reduced live cell numbers in LNCaP cells (Supplementary Fig. 9h, i).

Together, these cell and xenograft experiments reveal tumor-suppressive activities of LRIG1 in both AR^−/lo^ and AR^+^ human PCa models.

### Transgenic LRIG1 inhibits prostate tumorigenesis

**Fig. 4:**
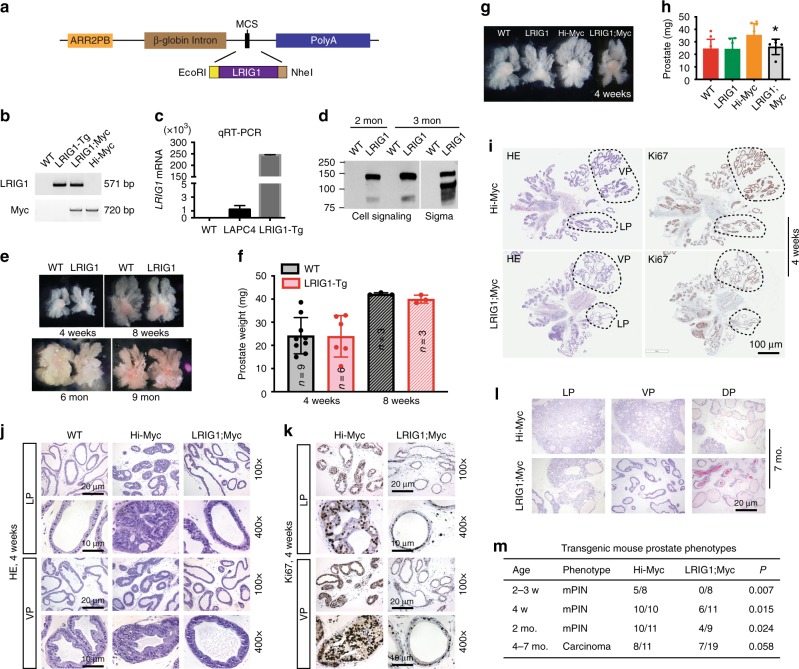
**Transgenic expression of LRIG1 inhibits Myc-driven tumorigenesis.** **a** Schematic of the *ARR2PB*-LRIG1 construct. Human LRIG1 cDNA (3,282 bp) was cloned into EcoRI/NheI sites of pPB197 vector. **b** PCR genotyping of transgenes LRIG1 (top) or c-Myc (below) using genomic DNA from mouse tails. **c** qPCR analysis of human *LRIG1* mRNA in prostates from both WT and LRIG1-Tg mice. LAPC4 cell RNA was used as a positive control. Results are representative of three experiments. **d** WB of human LRIG1 protein expression in the prostates of 2- or 3-month-old animals using two different human-specific LRIG1 antibodies. Note that Sigma mAb detected two bands at 143 kDa and ~110 kDa whereas Cell Signaling pAb detected mainly the 143 kDa band. **e** Representative whole-mount images of the prostates from WT and LRIG1-Tg mice at 4 and 8 weeks, and 6 and 9 months (*n* = 3–9 for various age groups). **f** Prostate weights of WT and LRIG1-Tg mice at 4 and 8 weeks (the number of animals analyzed are indicated in the bars). Note that at 8 weeks, the LRIG1-Tg prostates were slightly smaller than WT prostates (*P* = 0.0127; Student’s *t*-test). **g**, **h** The LRIG1;Myc prostate is smaller than Hi-Myc prostate. Shown are representative images of microdissected prostates (**g**) and average weight of prostate lobes (**h**) from the indicated genotypes at 4 weeks old (bars represent mean ± S.D and *n* = 9, 6, 8, and 6 for the four genotypes). In **h**, the *P*-value (*P < 0.05) was determined by Student’s *t*-test. **i** Aperio Scanscope images of HE (left) and Ki67 (right) stained sections of a pair of Hi-Myc and LRIG1;Myc prostates. Scale bar, 100 μm. **j**, **k** Representative HE (**h**) and Ki67 (**i**) images (original magnifications and scale bars indicated) in the LP and VP sections of indicated genotypes. **l** Representative HE images of prostates lobes in 7-month-old Hi-Myc and dTg mice. Scale bar, 20 μm. **m** Summary of prostate pathologies in the indicated genetic mouse models. Differences in incidence between the Hi-Myc and LRIG1;Myc phenotypes were analyzed by *χ*^2^ test. *Source data for Fig. 4c, d, f, h are provided as a Source Data file.

We further tested the tumor-suppressive functions of LRIG1 in genetic mouse models (Fig. 4; Supplementary Figs. 10–12). As LRIG1 is overexpressed in human PCa, we first asked whether transgenic (Tg) expression of LRIG1 might inhibit prostate tumor development in an autochthonous mouse model. To that end, we established an *ARR2PB*-LRIG1 Tg mouse model by overexpressing human LRIG1 cDNA in the mouse prostate using the *ARR2PB* promoter (Fig. 4a; Methods). As expected, the transgene LRIG1 was expressed only in the Tg mouse prostate with the ventral prostate (VP) expressing the highest level (Fig. 4b–d; Supplementary Fig. 10a). The LRIG1 Tg prostates were slightly smaller than the wild-type (WT) prostates at 8 weeks (Fig. 4e, f) but otherwise the two exhibited similar histological and morphological features (Supplementary Fig. 10b).

We subsequently crossed *ARR2PB*-LRIG1 Tg mice with the adenocarcinoma-prone Hi-Myc mice^52^ to establish the LRIG1;Myc double transgenic (dTg) mice (Fig. 4b). Why the Hi-Myc model? FIRST, Myc is overexpressed in the majority (~82%) of early PCa and precursor lesions called PIN (Prostate Intraepithelial Neoplasia)^53^ and represents a critical oncogenic driver of PCa development. SECOND, c-Myc alone is sufficient to immortalize normal human prostate (basal) epithelial cells^54^ and, in a gene dosage-dependent manner, to induce PIN^55^ and PCa^52^ in the mouse prostate. THIRD, c-Myc can cooperate with *Pten* mutation^56^ and also cross-regulates AR in a context-dependent manner during prostate tumorigenesis and development of castration resistance^57–62^. FOURTH, the Hi-Myc model is one of the few murine PCa models that present the spectrum of lesions, i.e., hyperplasia, PIN, adenocarcinomas, and local invasion that characterize human PCa^52^. FINALLY, there have been some reports on a potential reciprocal regulatory relationship between Lrig1 and c-Myc in mouse epidermis^29,30^.

In Hi-Myc prostates, the precursor lesion, mPIN^63^, could be observed in a fraction of animals as early as ~2 weeks and in most animals in 1–2 months^52^. By 6 months, virtually all Hi-Myc mice develop invasive adenocarcinoma^52^. Comparison of Hi-Myc and dTg prostates at different ages revealed that LRIG1 inhibited Hi-Myc mPIN and tumor development (Fig. 4g–m; Supplementary Fig. 11). At 4 weeks, the dTg prostates were slightly smaller than the age-matched Hi-Myc prostates (Fig. 4g, h). WM microdissection of the prostate (Fig. 4i), combined with histological analysis (Fig. 4j), revealed less prominent mPIN phenotypes in dTg prostates, which was associated with reduced Ki67^+^ cells (Fig. 4k). LRIG1 expression inhibited mPIN by ~50% in 4–8-week-old animals (20/21 in Hi-Myc vs. 10/20 in dTg; *P* < 0.0001, *χ*^2^ test) (Fig. 4m). The tumor-suppressive effects of LRIG1 were still observed in 4–7-month-old dTg prostates although the effects became attenuated (Fig. 4l, m; Supplementary Fig. 11).

In pilot studies, transgenic expression of LRIG1 also inhibited aggressive TRAMP tumors induced by SV40 T/t expression in the mouse prostate^64,65^ (Supplementary Fig. 12). We analyzed a total of 6 TRAMP and 9 LRIG1;TRAMP dTg mice at 3 months. As illustrated in Supplementary Fig. 12a, b, in the 3 TRAMP prostates, two (21/10 and 21/1) showed tumors (black circles) whereas one (21/N) looked largely normal except for mild to moderate hyperplasia (white circle). In contrast, the 2 LRIG1;TRAMP prostates (22/N and 21/3) presented normal features. In total, 4 out of the 6 TRAMP prostates at 3 months showed apparent tumors (67%) whereas only 1 out of 9 (11%) LRIG1;TRAMP prostates had tumors (*P* < 0.0001; *χ*^2^ test). As observed earlier^65^, the TRAMP tumors at 3 months showed highly pleiomorphic features; for example, tumor in animal 21/10 showed typical neuroendocrine (NE) characteristics and the large tumor in animal 21/1 presented highly variegated morphological and histological features (Supplementary Fig. 12c). In contrast, the 21/N TRAMP prostate as well as most of the 3-month-old LRIG1;TRAMP prostates looked largely benign with mild hyperplasia (for 21/N) and micro-tumor foci (Supplementary Fig. 12c, right, circle). Consequently, prostate weights in 3-month-old LRIG1;TRAMP dTg mice were smaller than in TRAMP mice (Supplementary Fig. 12d). LRIG1 inhibition of TRAMP tumors was associated with reduced tumor cell proliferation (Supplementary Fig. 12e).

### LRIG1 inhibits castration-resistant PCa

**Fig. 5:**
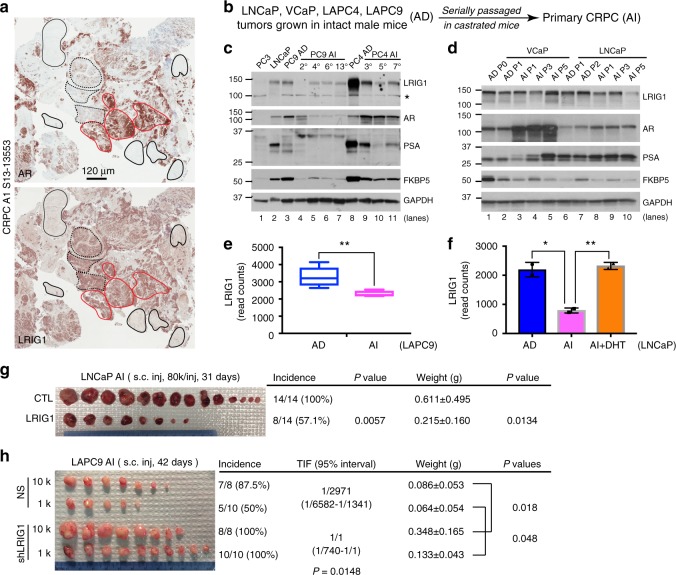
**Expression and tumor-suppressive functions of LRIG1 in CRPC.** **a** Heterogeneous LRIG1 expression and discordant AR/LRIG1 expression patterns in CRPC. Contiguous WM sections from a CRPC (case# indicated on left) were stained for (top) AR (mouse mAb from Santa Cruz clone 441; 1:100 dilution)) and (bottom) LRIG1 (Sigma, 1:100 dilution). AR^+/hi^/LRIG1^hi^, AR^−/lo^/LRIG1^lo^, and AR^−/lo^/LRIG1^hi^ areas are marked by solid red, solid black, and dashed black circles, respectively (note that the AR IHC image represented part of the image in Fig. 1c of ref. ^51^). **b** Experimental scheme to generate castration-resistant (androgen-independent [AI]) xenograft tumors from the corresponding androgen-dependent (AD; androgen-sensitive) parent tumors (see Methods). **c**, **d** Reduced but persistent LRIG1 expression in experimental CRPC. Whole lysate of AD and AI tumors at the indicated passages (P) was used in WB for molecules indicated. The asterick in **c** represents a non-specific band detected by Sigma anti-LRIG1 antibody. **e**, **f** Reduced *LRIG1* mRNA levels in LAPC9 (**e**) and LNCaP (**f**) AI tumors. Presented are the raw read counts of *LRIG1* mRNA fragments in RNA-seq of AD/AI tumors (*n* = 5 each; ref. ^51^). For the LNCaP model, a condition of “AI + DHT” was added. *P < 0.05; **P < 0.01 (unpaired multiple *t*-test in GraphPad and statistical significance determined using the Holm-Sidak method). The centerlines in box plots **e** show the mean values, box edges are 25th and 75th percentiles and whiskers represent minimum and maximum values. **g** LRIG1 overexpression in LNCaP AI cells inhibits CRPC regeneration. LNCaP cells were freshly purified out from AI tumors and infected with control (CTL) or pLVX-LRIG1 (LRIG1) lentiviral vectors (MOI 5, 48 h), and subcutaneously (s.c) injected in castrated NSG mice. Tumor incidence, tumor weight, and *P* values are indicated. **h** Knocking down endogenous LRIG1 in LAPC9 AI cells promotes CRPC regeneration. LAPC9 AI cells were purified out from xenografts and infected with control (NS) or shLRIG1 vectors (MOI 10, 12 h), and s.c injected (at two cell doses) in castrated male NOD/SCID mice. Tumor incidence, TIF (tumor-initiating frequency), tumor weight, and *P* values are indicated. *Source data for Fig. 5c, d, g, h are provided as a Source Data file.

Preceding experiments demonstrate tumor-suppressive functions of LRIG1 in both human xenograft and murine genetic PCa models. Next, we investigated the expression and function of LRIG1 in treatment-failed PCa and castration-resistant PCa (CRPC; Fig. 5; Supplementary Fig. 13). Consistent with earlier observations (Fig. 1a–c), *LRIG1* mRNA in TCGA PRAD dataset was increased in early-stage Gleason 6 tumors compared with normal tissues (Supplementary Fig. 13a). Interestingly, however, the *LRIG1* mRNA levels showed a decreasing trend (*P* = 0.00547, Jonckheere-Terpstra test) with increasing tumor grade, i.e., from GS (Gleason Score) 6 to GS9 tumors (Supplementary Fig. 13a). Notably, *LRIG1* mRNA expression persisted in the 66 treated PCa in TCGA (Supplementary Fig. 13a). At the protein level, unlike relatively homogeneous expression of LRIG1 in untreated tumors (Fig. 1d), IHC analysis in 26 (20 in a TMA and 6 WM) patient CRPC specimens^49,51^ revealed heterogeneous LRIG1 expression, as well as discordant AR and LRIG1 expression patterns (Fig. 5a; Supplementary Fig. 13b–d). In WM CRPC slides, both concordant, AR^+^/LRIG1^+^ (Fig. 5a; red solid circles) and AR^−/lo^/LRIG1^lo^ (Fig. 5a; black solid circles), as well as discordant, AR^−/lo^/LRIG1^+^ (Fig. 5a; black dashed circles; Supplementary Fig. 13d), areas were observed. Consistent with our previous observations^51^, CRPC was enriched in AR^−/lo^ PCa cells, which, strikingly, were still mostly LRIG1^+/hi^ (Supplementary Fig. 13d). Of note, we performed AR IHC using N-ter antibodies (441 and N20; Supplementary Table 1), which would capture both full-length AR and all C-ter truncated variants.

To determine LRIG1 functions in CRPC, we serially transplanted parental AD LNCaP, VCaP, LAPC4, and LAPC9 tumors in castrated immunodeficient mice to establish castration-resistant (androgen-independent; AI) tumors^45,46,49,51^ (Fig. 5b; Methods). Consistent with previous observations^51^, Western blotting analysis demonstrated that serially passaged LNCaP, VCaP, and LAPC4 AI tumors showed increasing levels of AR while the LAPC9 AI tumors showed decreasing AR (Fig. 5c, d). However, in all AI models, the canonical AR targets FKBP5 and/or PSA gradually decreased, so did LRIG1 (Fig. 5c, d). The *LRIG1* mRNA levels in LAPC9 and LNCaP AI tumors also decreased based on RNA-seq analysis (Fig. 5e, f). Overexpression of LRIG1 in LNCaP AI cells exhibited inhibitory effects on both tumor incidence and growth (Fig. 5g) whereas knocking down endogenous LRIG1 in LAPC9 AI cells promoted both tumor regeneration and growth in a limiting-dilution tumor assay (Fig. 5h). In addition, knocking down LRIG1 in LAPC4 and LAPC9 AI cells enhanced their sphere formation capabilities (Supplementary Fig. 13e, f). Together, these results suggest that LRIG1 also inhibits CRPC.

### LRIG1 inhibits pre-established AR^−^ PCa xenografts

**Fig. 6:**
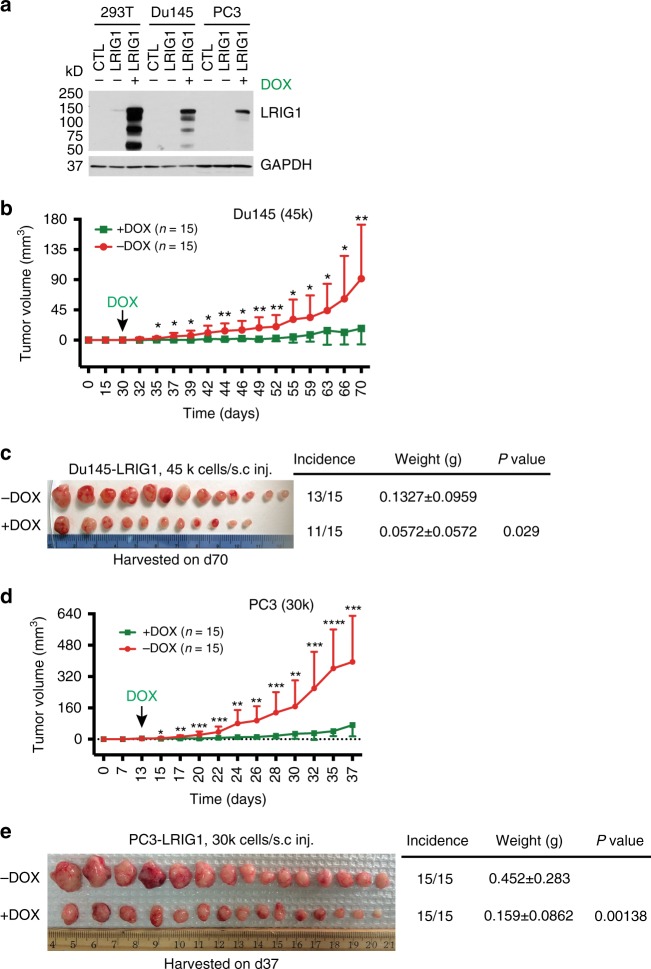
**Inducible LRIG1 expression inhibits pre-established AR**^−^ **PCa xenografts.** **a** Establishing doxycycline (DOX) inducible LRIG1-expressing PCa cell models. The AR^-^ Du145 and PC3 cells (293T, control cells) were infected with the pLVX-TetOne-LRIG1-Puro lentivirus (MOI 10; Supplementary Fig. 5d) for 72 h followed by puromycin selection (~2 weeks). Cell lysate prepared from cells in the absence or presence of DOX (0.1 μg/ml; 48 h) were used in Western blotting analysis of LRIG1 (GAPDH was used as control). **b**, **c** LRIG1 induction inhibits growth of pre-established Du145 xenograft tumors. Du145 cells were implanted subcutaneously (s.c; 45,000 cells/injection) in NOD/SCID mice and, 30 days later, DOX was added to the food (2 mg/kg body weight; arrow) of one group of animals (*n* = 15 for each group). Tumor volume was measured (**b**; *P < 0.05, **P < 0.01, two-tailed unpaired Student’s *t*-test). **c** Animals were terminated on day 70 post implantation and shown are tumor images, tumor incidence, endpoint tumor weights (mean ± SD), and the *P* value (Student’s *t*-test). **d**, **e** LRIG1 induction inhibits growth of pre-established PC3 xenograft tumors. PC3 cells were implanted subcutaneously (s.c; 30,000 cells/injection) in NOD/SCID mice and, 13 days later, DOX was added to the food (2 mg/kg body weight; arrow) of one group of animals (*n* = 15 for each group). Shown in **d** are tumor growth curves and arrows indicate the time (day) when DOX was administered (*P < 0.05; **P < 0.01; ***P < 0.001; Student’s *t*-test). **e** Tumor weight and image. *P*-value was determined using two-tailed unpaired Student’s *t*-test. *Source data for Fig. 6a–e are provided as a Source Data file.

Consistent inhibitory effects of LRIG1 on both AD and AI (castration-resistant) PCa xenografts as well as in two genetic prostate tumor models prompted us to ask whether LRIG1 might possess therapeutic potential. To this end, we established doxycycline (DOX) responsive LRIG1-overexpressing system (Supplementary Fig. 5d) in AR^−^/LRIG1^−/lo^ Du145 and PC3 cells (Fig. 6a). About 1 month after establishing the Du145 xenografts, DOX induction of LRIG1 inhibited the growth rate of xenografts and also reduced sizes of the endpoint tumors (Fig. 6b, c). Of note, the  LRIG1 expression levels in endpoint Du145 TetOne-LRIG1-Puro tumors were within the range of the endogenous LRIG1 levels in AD LNCaP, VCaP, and LAPC9 cells and/or xenografts (Supplementary Fig. 13g). Similarly, ~2 weeks following establishing PC3 xenografts, LRIG1 induction inhibited both tumor growth rate and weights (Fig. 6d, e). These results suggest that induction of LRIG1 expression inhibits pre-established AR^−^, androgen-insensitive PCa in vivo.

### LRIG1 is directly regulated by AR

**Fig. 7:**
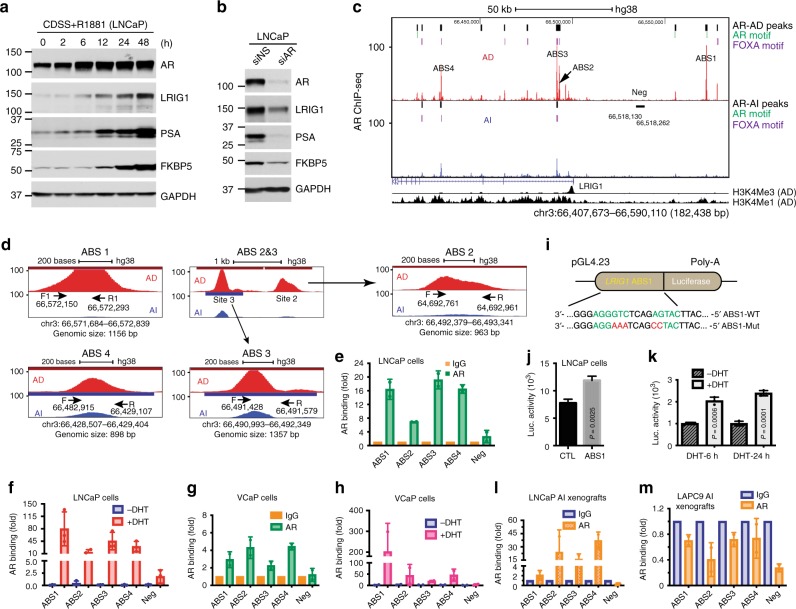
**LRIG1 is directly regulated by AR in PCa cells.** **a** R1881-induced AR is accompanied by increased LRIG1 and other AR targets. LNCaP cells cultured in CDSS (48 h) were treated with R1881 (0.1 nM) for the intervals indicated. **b** AR knockdown reduces LRIG1 and AR targets. LNCaP cells were treated by AR-targeting siRNAs (10 nM; 48 h), and whole cell lysate used in WB. **c** AR binds to multiple ABS in *LRIG1* genomic locus. Shown on top are 4 major potential ABS in AD LNCaP cells identified by AR ChIP-seq (Neg: genomic region used as negative control in ChIP-qPCR). Shown on right are AR peaks (AD, top; AI, bottom) and canonical AR and FOXA motifs. Shown below are H3K4me3 and H3K4me1 peaks in LNCaP AD cells. **d** Zoom-in presentation of potential AR binding sites (ABS1-4; sizes indicated below) in the *LRIG1* genomic region. For each ABS, the primers (F, forward primer; R, reverse primer) used for ChIP-qPCR were indicated with arrows. **e**, **f** ChIP-qPCR analysis of AR binding to ABS1 – 4 in regular (**e**) or DHT-stimulated (**f**) LNCaP cells. AR binding to ‘Neg’ region was used as control and ChIP results were normalized to IgG. Bars represent the mean ± S.D (*n* = 3). **g**, **h** ChIP-qPCR analysis of AR binding to LRIG1 genomic region in VCaP cells. Shown are ChIP-qPCR results of AR binding to ABS1-4 in regular VCaP (**g**; normalized to IgG) or VCaP cells stimulated with DHT (10 nM, 2 h; normalized to -DHT) (**h**). Bars represent the mean ± S.D (*n* = 3). **i** Schematic of *LRIG1*-ABS1 pREPORT luciferase constructs. A wild-type (WT) or mutated (Mut) *LRIG1*-ABS1 fragment (~100 bp) that harbors an ARE (AR-Responsive Element) was used to drive luciferase expression. **j**, **k** Luciferase assays of ABS1 in LNCaP cells (**j**) and responsiveness of ABS1-luc to DHT (**k**) (mean ± S.D; *n* = 3). *P*-values (Student’s *t*-test) are indicated. **l**, **m** ChIP-qPCR analysis of AR binding to LRIG1 ABS1-4 in LNCaP (**l**) and LAPC9 (**m**) AI xenografts. All results were normalized to IgG (mean ± S.D; *n* = 3). *Source data for Fig. 7 (representative gel images) are provided as a Source Data file.

Earlier studies from others showed that LRIG1 appears to be an androgen-responsive gene^44,66^. We have also observed that: (1) LRIG1 was preferentially expressed in AR^+^ PCa cell types (Supplementary Fig. 6); (2) LRIG1 was generally decreased in experimental CRPC models (Fig. 5c–f) and re-administration of testosterone to mice bearing LNCaP AI tumors restored LRIG1 mRNA expression (Fig. 5f); (3) LRIG1 showed changes in CRPC models similar to canonical AR targets PSA and FKBP5 (Fig. 5c, d); (4) both *LRIG1* (Fig. 1c) and *AR* (Supplementary Fig. 14a) mRNA levels were increased in PCa compared to matched normal prostate tissue and the two positively correlated with each other, especially in low-grade (Gleason 6–7) tumors (Supplementary Fig. 14b, c); (5) in 7 PCa patients treated with ADT (GSE48403)^67^, post-treatment tumors expressed significantly reduced mRNA levels of *LRIG1* (and *PSA*) compared to the pre-ADT tumors from the same patients (Supplementary Fig. 14d); and (6) AR activation by synthetic androgen R1881 in AR^+^ PCa cells such as LNCaP (Fig. 7a) and VCaP (Supplementary Fig. 14e) cells induced PSA and FKBP5 as well as LRIG1 whereas knocking down endogenous AR reduced all 3 proteins (Fig. 7b; Supplementary Fig. 14f). These observations raise the possibility that LRIG1 may be directly regulated by AR.

To test this possibility, we analyzed the AR chromatin immunoprecipitation (ChIP)-seq data in AD LNCaP cells (GEO#: GSM699631) and observed multiple potential AR-binding sites (ABS) or peaks across the ~182 kb *LRIG1* genomic region, some of which harbored AR and/or FOXA motifs (Fig. 7c, d; Supplementary Fig. 14g, h). We focused on 4 major AR-binding peaks labeled as ABS1 – 4, which were 1156, 963, 1357, and 898 bp, respectively (Fig. 7c, d). ABS1 was located >50 kb upstream of the transcription start site (TSS) of the *LRIG1* gene, and ABS2 and ABS3 were neighboring each other and located close to the TSS/promoter region that coincided with the active histone modification mark H3K4me3 whereas ABS4 was localized to the intronic region characterized with the enhancer histone modification mark H3K4me1 (Fig. 7c). Only ABS1 contained a canonical AR-binding motif whereas ABS2-ABS4 harbored FOXA motif in AD LNCaP cells (Fig. 7c; Supplementary Fig. 14g, h). ChIP-qPCR analysis in regular serum-cultured (AD) LNCaP and VCaP cells revealed various degrees of AR binding to all 4 ABS (Fig. 7e and g), and the binding was enhanced by DHT (dihydrotestosterone) in both cell types (Fig. 7f and h). Interestingly, all 4 ABS in AD LNCaP cells were associated with the histone modification H3K27ac enhancer mark (Supplementary Fig. 14i), which is associated with active gene transcription. Luciferase reporter assays using an ABS1 fragment (~100 bp) harboring the AR-binding motif (Fig. 7i) revealed significant luciferase activity in transfected LNCaP cells (Fig. 7j), and this baseline luciferase activity was further stimulated by DHT (Fig. 7k) but eliminated by mutating the AR-binding sequence (Fig. 7i; Supplementary Fig. 14j). Together, these data support that AR directly regulates LRIG1 in untreated AD PCa cells.

We subsequently studied whether AR might still transcriptionally regulate LRIG1 expression in CRPC cells. As presented earlier, 3 CRPC models (LAPC4, VCaP, and LNCaP) showed increasing levels of AR during castration while the LAPC9 model displayed reducing AR although all 4 AI models showed reduced LRIG1 protein, especially at late passages (Fig. 5c, d). Consistently, in AR^−/lo^ LAPC9 AI tumors, the *LRIG1* mRNA levels decreased as measured by RNA-seq (Fig. 5e) and quantitative RT-PCR (qRT-PCR; Supplementary Fig. 14k) analyses. Surprisingly, in AR^+/hi^ LNCaP AI tumors, the *LRIG1* mRNA levels also decreased (Fig. 5f), suggesting that in such AI tumors/clones, AR may have shifted from regulating conventional pro-differentiation targets such as PSA and LRIG1 to other molecular targets^68,69^. In support, in AI LNCaP cells (GEO#: GSM699630), the ABS1 peak virtually disappeared and the ABS2 – ABS4 peaks significantly reduced (Fig. 7c). This observation suggests that the ABS1 may represent the major *cis* element in the *LRIG1* genomic region through which AR regulates LRIG1 transcription, which would be consistent with only ABS1 harboring a canonical AR-binding motif (Fig. 7c). On the other hand, as both patient CRPC (Fig. 5a; Supplementary Fig. 13b–d) and experimental CRPC models (Fig. 5c–f) clearly expressed significant levels of LRIG1, we suspected that residual AR binding to ABS2-ABS4 (Fig. 7c) might be involved, at least partially, in mediating LRIG1 transcription in AI PCa cells. In support, ChIP-qPCR analyses revealed both AR binding to (Fig. 7l), and H3K27ac enhancer association with (Supplementary Fig. 14l), the ABS2-4 (but not ABS1) of *LRIG1* in LNCaP AI xenograft cells. Interestingly, unlike AR binding profiles in AD LNCaP cells (Fig. 7e, f), AR primarily bound to the ABS2 and ABS4 with negligible binding to ABS1 in AD LAPC9 cells (Supplementary Fig. 14m), suggesting cell type-dependent AR regulation of LRIG1 in AD PCa cells. In LAPC9 AI tumors, there was no AR binding to any of the 4 ABS (Fig. 7m), suggesting that LRIG1 expression in AR^−/lo^ LAPC9 cells is likely regulated by other mechanisms.

### Inter-relationship between LRIG1, ERBBs, and AR in PCa

**Fig. 8:**
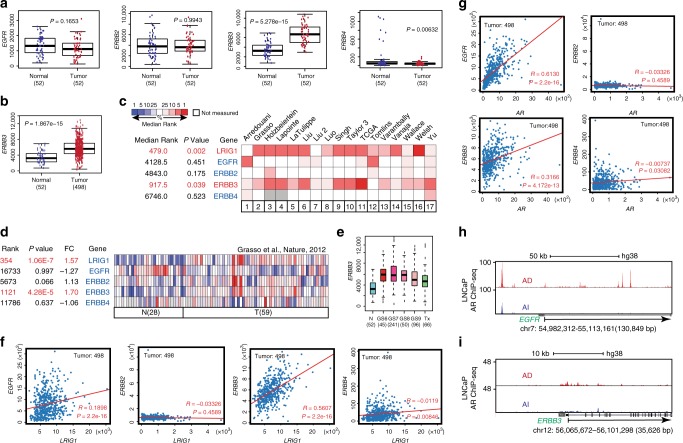
**Interrelationship between LRIG1, ERBBs and AR in PCa.** **a** Increased *ERBB3* and low expression of *ERBB4* mRNA levels in PCa. Shown are box plots of the mRNA levels (normalized RSEM) of 4 ERBBs in 52 matched normal vs. tumors in TCGA PRAD dataset. *P*-values are indicated (two-tailed unpaired Student’s *t*-test). **b** Increased *ERBB3* mRNA in 498 prostate tumors compared with 52 normal tissues in TCGA PRAD dataset. *P*-value was calculated using two-tailed unpaired Student’s *t*-test. **c**, **d** Co-upregulation of *LRIG1* and *ERBB3* mRNA levels in 17 Oncomine PCa datasets (**c**) and Grasso dataset (**d**). **e** The *ERBB3* mRNA expression pattern resembles that of *LRIG1* in TCGA PRAD dataset (see Supplementary Fig. 13a). Note that *ERBB3* mRNA levels are significantly higher in GS6 tumors than in normal (N) tissues (*P* < 0.0001; Student’s *t*-test) but then decreased with increasing Gleason grade (*P* < 0.001; Jonckheere-Terpstra test). **f** Only *ERBB3* mRNA levels correlate with *LRIG1* mRNA levels in TCGA PRAD dataset, as determined by linear regression analysis (R and *P* values indicated). **g** Positive correlations between *AR* and *EGFR*, and, less significantly, between *AR* and *ERBB3* mRNA levels. mRNA levels of *AR* and 4 *ERBB* genes were downloaded from TCGA 498 prostate tumors and used to construct Pearson correlation linear regression plots (R and *P* values indicated). **h** Potential AR binding to *EGFR* gene. Shown is the AR ChIP-seq binding profile in LNCaP AD (top; GSM699631) vs. AI (bottom; GSM699630) cells. Note that *EGFR* gene has a very long intron 1 (>100 kb) between the first and second exons and therefore, only AR-binding profile in the first exon was shown. Several prominent AR binding peaks are observed in the first exon of the *EGFR* gene in AD cells that are all lost in AI cells. **i** Potential AR binding to *ERBB3* gene. AR binding peaks were extracted from the same AR ChIP-seq data in AD (top) and AI (bottom) LNCaP cells shown above. Also see Supplementary Fig. 15f. For box plots in **a**, **b**, and **e**, the centerlines represent the medians, box edges 25th and 75th percentiles, and whiskers the maximum and minimum values.

**Fig. 9:**
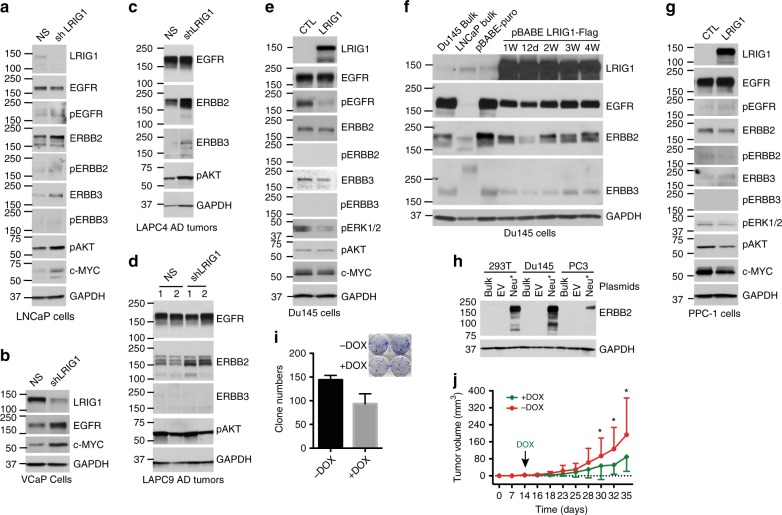
**LRIG1 inhibits ERBBs in PCa cells and Neu-promoted PCa growth.** **a**–**d** Knocking down endogenous LRIG1 in AR^+^ PCa cells upregulates (p)ERBB members in a cell type-dependent manner. Shown are WB of the molecules indicated (80 μg WCL/lane). For a-b, cells were infected with shLRIG1 (or NS control) lentiviral vectors (MOI 10) for 72 h. For **c**, **d** 1 pair of endpoint LAPC4 tumors (**c**; see Fig. 3b) and 2 pairs of endpoint LAPC9 AD tumors (**d**; see Fig. 3c) were used in WB analysis of the molecules indicated. **e–g** Overexpressing LRIG1 in AR^-^ Du145 (**e**, **f**) and PPC-1 (**g**) cells downregulates (p)ERBB members. Shown are WB of the molecules indicated (80 μg protein/lane). For **e** and **g**, cultured cells were infected with LRIG1 (or CTL) lentiviral vectors (MOI 10) for 72 h. For **f**, samples in Supplementary Fig. 8c were used in WB analysis of the molecules indicated. **h**–**j** LRIG1 inhibits Neu*-driven PCa growth. **h** WB showing Neu* expression in 293T, Du145 and PC3 cells (20 μg whole cell lysate/lane) that stably expressed Neu* and inducible LRIG1 (upon puromycin selection) using an anti-human ERBB2 antibody (note that PC3 and Du145 cells expressed relatively low levels of endogenous ERBB2 (see Supplementary Fig. 6b) and the film was exposed for a short period of time to highlight the overexpressed Neu*). EV, empty vector. **i** LRIG1 expression inhibits clonogenicity of PC3-Neu* cells. PC3 cells were infected with pLVX-Neu* lentivirus (Supplementary Fig. 5e; MOI 10) together with the lentiviral vector encoding DOX-inducible LRIG1 (Supplementary Fig. 5d). Cells were plated (200 cells/well) in quadruplicate in the absence or presence of DOX (100 ng/ml) and clones were enumerated 2 weeks later. Presented are the clone numbers in the two groups (mean ± S.D; *n* = 3 independent experiments). Shown in the inset is a representative micrograph. **j** DOX-induced LRIG1 expression inhibited Neu*-driven PC3 tumor growth. PC3 Neu*/LRIG1-puro cells were subcutaneously implanted in male NOD/SCID mice (15 K cells/injection; *n* = 15 mice/group). Tumor volume was measured starting from 2 weeks post implantation. *P < 0.05 (Student’s *t*-test). *Source data for Fig. 9 (representative gel images) are provided as a Source Data file.

**Fig. 10:**
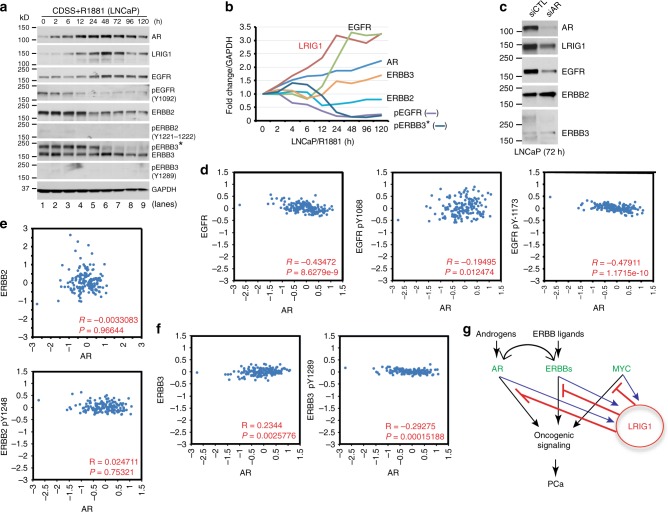
**AR induces LRIG1 to curb ERBB signaling: a model.** **a**, **b** R1881-induced AR upregulation is accompanied by increased LRIG1 and dynamic downregulation of (p)ERBB members in LNCaP cells. LNCaP cells cultured in CDSS (48 h) were treated with R1881 (0.1 nM) for up to 120 h (R1881 was replenished every 24 h) and whole cell lysate (80 μg/lane) was used in WB for the molecules indicated. Protein bands were scanned by densitometry and shown in **b** is the quantification of average (*n* = 2) protein levels of AR, LRIG1, and (p)ERBB members normalized to GAPDH levels at time 0 (1.0). Note that pERBB2 (Y1221-1222) and pERBB3 (Y1289) bands were too faint to scan. pERBB3* was the (putatively) phosphorylated ERBB3 band detected by the mouse mAb against ERBB3 (Millipore 05-390; see Supplementary Table 1). As can be seen, AR induction was accompanied by increased protein levels of AR, LRIG1, EGFR, ERBB3 but decreased levels of pEGFR, ERBB2, and pERBB3. **c** AR knockdown reduces LRIG1 and EGFR in LNCaP cells. LNCaP cells were treated by AR-targeting siRNAs (10 nM), and whole cell lysate used in WB for the molecules indicated. The results are representative of 2 independent experiments. **d**–**f** Potential relationship between AR and (p)ERBB protein levels in the human PCa TCPA database (see Text and Methods). The correlation coefficients (R values) and P-values were determined by linear regression analyses. **g** A model depicting that LRIG1 is feedback induced by three major signaling pathways in PCa, i.e., AR, c-MYC, and ERBBs, to neutralize/antagonize their oncogenic activities. The model also depicts cross-regulatory relationship between AR and ERBBs. *Source data for Fig. 10 (representative gel images) are provided as a Source Data file.

The above experiments demonstrate that LRIG1 is directly regulated by AR. As LRIG1 is induced by EGF and functions in a feedback loop to dampen the EGF/EGFR and other ERBB and RTK signaling^2,15–21^, and also because ERBB and AR signaling cross-regulates each other and PCa development and castration resistance^70–73^, we explored the inter-relationship between AR, LRIG1 and ERBBs in PCa and potential involvement of ERBB members in LRIG1-mediated inhibition of PCa (Figs. 8–10; Supplementary Fig. 15). Interrogation of mRNA levels of the 4 ERBB members in TCGA PRAD dataset revealed high levels of *ERBB1-ERBB3* with minimal expression of *ERBB4* (Fig. 8a). Interestingly, *ERBB3* mRNA levels were significantly increased in prostate tumors compared with matched normal tissues (Fig. 8a, b). Examination of 17 Oncomine datasets also revealed significant and generally increased levels of ERBB3 expression in PCa (Fig. 8c, d; Supplementary Fig. 15a–e). In several Oncomine datasets, EGFR and/or ERBB2 mRNA levels also increased in prostate tumors compared to normal tissues (Supplementary Fig. 15d, e). Intriguingly, the *ERBB3* mRNA expression pattern in prostate tumors of different Gleason grade was very similar to that of *LRIG1* (Fig. 8e; compare with Supplementary Fig. 13a) and, in fact, *LRIG1* mRNA levels correlated with those of *ERBB3* but not of other *ERBB* members (Fig. 8f). Since AR directly regulates LRIG1, we explored the potential relationship between AR and ERBB members at the mRNA levels, and observed a strong correlation between *AR* and *EGFR* and a modest correlation between *AR* and *ERBB3* but no correlation between *AR* and *ERBB2* and *ERBB4* (Fig. 8g). Interrogation of the AR ChIP-seq data in LNCaP AD/AI cells revealed multiple AR binding peaks in the intron 1 of *EGFR* gene in AD cells, most of which disappeared or significantly decreased in AI cells (Fig. 8h). In contrast, only low levels of AR binding was observed in the *ERBB3* coding region in LNCaP AD cells, several of which showed modest decreases in AI cells (Fig. 8i; Supplementary Fig. 15f). These bioinformatics analyses suggest that in prostate tumors, (1) ERBB3 mRNA levels are upregulated; (2) LRIG1 and ERBB3 mRNA levels correlate with each other; (3) AR and EGFR mRNA levels correlate strongly with each other and AR may directly regulate EGFR transcription; and (4) AR and ERBB3 mRNA levels modestly correlate with each other and AR may not significantly regulate ERBB3 transcription via direct binding.

### LRIG1 inhibits ERBBs and retards Neu-promoted PCa growth

We investigated alterations of ERBB proteins in LRIG1-mediated inhibition of PCa using the PCa cell and xenograft models described earlier, all of which expressed EGFR at various levels while only some expressed ERBB2 and ERBB3 (Supplementary Fig. 6b) with no appreciable ERBB4 in any of the models. Knocking down endogenous LRIG1 in 4 AR^+^ PCa cells/tumors (i.e., LNCaP, VCaP, LAPC4, and LAPC9) upregulated ERBBs in a model-dependent manner, exemplified by ERBB2 upregulation in 3 models (LNCaP, LAPC4, LAPC9), EGFR increase in VCaP, and ERBB3 increase in LAPC4 (Fig. 9a–d). In LNCaP and LAPC4 models, LRIG1 knockdown resulted in increased pAKT (Fig. 9a, c), a downstream signaling target of ERBB activation. Note that all these 4 AR^+^ PCa models generally expressed very low levels of phosphorylated ERBB (pERBB) proteins (Fig. 9a). In contrast, lentiviral-mediated transient LRIG1 overexpression in AR^−^ Du145 cells led to decreases in ERBB 1-3 and pEGFR (Fig. 9e), and retroviral-mediated stable LRIG1 expression in Du145 cells also resulted in decreased ERBB1-3 (Fig. 9f). On the other hand, LRIG1 expression in AR^−^ PPC-1 cells only modestly reduced ERBB2 leading to reduced pERK1/2 and pAKT levels (Fig. 9g).

The above results suggest that LRIG1 may suppress PCa, at least partly, through antagonizing specific ERBB members in different PCa cell types. To directly test this suggestion, we established PC3 and Du145 cells that expressed a mutant rat Neu (ErbB2) oncogene (Neu*)^74,75^ and also inducible LRIG1 (Fig. 9h). LRIG1 induction inhibited clonogenicity (Fig. 9i; *P* = 0.02, Student’s *t*-test) and tumor growth (Fig. 9j) of PC3-Neu* cells.

### Inverse correlation of (p)ERBBs and LRIG1 in human PCa

We treated LNCaP cells with R1881 for up to 120 h to further explore the dynamic relationship between LRIG1 and (p)ERBBs in the context of androgen/AR signaling. R1881 coordinately induced AR, LRIG1 and EGFR (Fig. 10a, b), and knocking down AR reduced not only LRIG1 but also EGFR (Fig. 10c). These latter observations are consistent with potential direct regulation of EGFR by AR^70^ (Fig. 8h). R1881/AR activation only slightly induced ERBB3 (Fig. 10a, b). Strikingly, accompanying LRIG1 induction in LNCaP cells, pEGFR(Y1092), ERBB2, and pERBB3 proteins were all decreased (Fig. 10a, b), suggesting the possibility that LRIG1 was induced by AR to antagonize (p)ERBBs and inhibit their signaling. In support of this conjecture, in human PCa samples, examination of TCPA (The Cancer Proteome Atlas, tcpaportal.org)^76^ data revealed a strong inverse correlation between AR and EGFR/pEGFR(pY1173), a moderate inverse correlation between AR and ERBB3/pERBB3 (pY1289), and no significant inverse correlation between AR and ERBB2/pERBB2(Y1248) (Fig. 10d-f).

### Evidence that LRIG1 inhibits c-Myc expression in PCa

Finally, since earlier studies revealed a reciprocal relationship between Lrig1 and c-Myc in the mouse epidermis^29,30^ and we observed LRIG1-mediated inhibition of Hi-Myc tumors (Fig. 4), we probed c-Myc protein levels in human PCa cells upon manipulation of LRIG1. Interestingly, knocking down LRIG1 in VCaP and LNCaP cells led to ~3-4 fold increase in c-Myc (Fig. 9a, b) whereas overexpression of LRIG1 led to ~2-fold reduction in c-Myc in Du145 and PPC-1 cells (Fig. 9e, g). These data suggest that endogenous LRIG1 in human PCa cells may antagonize c-Myc.

## Discussion

The present study combines cell, xenograft and genetic mouse models and human PCa specimens as well as bioinformatics analyses to investigate expression, regulation, functions and mechanisms of LRIG1 in PCa. Early studies have reported LRIG1 downregulation in skin, renal, bladder, cervix, and lung cancers and LRIG1 upregulation in carcinoid lung cancers, leukemia, and astrocytomas^18^. The only earlier study on LRIG1 in PCa revealed contrasting LRIG1 expression in two cohorts of PCa patients^44^. Interrogation of *LRIG1* mRNA levels in 31 human cancers in TCGA reveals that *LRIG1* is downregulated in bladder, cervical, colon/rectum, and thyroid cancers and melanoma but upregulated in esophageal carcinoma, low-grade glioma and GBM, thymoma, B-cell lymphoma, AML, and PCa, and the trend of *LRIG1* upregulation is also observed in several other cancers (Supplementary Fig. 16). Our own analysis indicates that *LRIG1* mRNA is upregulated in 1160 PCa samples (662 in Oncomine and 498 in TCGA; Fig. 1a–c) and LRIG1 protein is upregulated in 326 PCa samples (314 in TMAs and 12 WM specimens; Fig. 1e–g).

Despite its overexpression in human PCa, LRIG1 expression correlates with better patient survival, suggesting a tumor-suppressive function. Subsequent functional studies in multiple human xenograft and two mouse genetic models establish that LRIG1, which is rarely mutated in PCa, acts as a functional tumor suppressor in PCa (Figs. 2–5; Supplementary Figs. 7–12). The striking finding that knocking down endogenous LRIG1 in 3 AR^+^ PCa models promotes tumor regeneration/growth suggests that LRIG1 intrinsically represses tumorigenicity of AR^+^ PCa cells. In contrast, LRIG1 overexpression in AR^−^ PCa models inhibits pre-established PCa, thus implying a therapeutic potential of LRIG1,

One of the most significant findings of this study is that AR directly transactivates LRIG1 via binding to several ABS in *LRIG1* genomic locus in association with active histone marks including H3K4me3 (promoter), H3K4me1 (enhancer), and H3K27ac (enhancer). The LRIG1 expression pattern in AD/AI prostate tumors is reminiscent of LRIG1 response to estrogen stimulation and ER inhibitors in breast cancer^9,77,78^. Interestingly, LRIG1 expression in CRPC becomes heterogeneous and discordant with AR expression resulting in subpopulations of PCa cells with AR^+^/LRIG1^+^, AR^−/lo^/LRIG1^−/lo^, and AR^−/lo^/LRIG1^+^ phenotypes. Notably, in castrated (AI) PCa cells, although AR binding to ABS1 disappears, there remain low levels of AR binding to ABS2 – ABS4 potentially explaining the AR^+^/LRIG1^+^ phenotype. In AR^−/lo^/LRIG1^−/lo^ CRPC cells, loss of LRIG1 expression presumably results from lack of positive regulation by AR and/or epigenetic silencing such as promoter hypermethylation^79^. It will be very interesting to investigate how LRIG1 is expressed in AR^−/lo^ (i.e., AR^−/lo^/LRIG1^+^) PCa cells. As the AR^−/lo^ PCa such as LAPC9-CRPC is greatly enriched in cancer stem cells^46,47,49,51^ and frequently overexpresses stemness factors such as NANOG^69^, it is tempting to speculate that in such tumors/clones, stemness factors predominantly regulate LRIG1. Regardless, LRIG1 still exerts tumor-suppressive functions in CRPC (Fig. 5g, h), suggesting that in treatment-failed tumors, LRIG1, positively regulated by AR and, possibly, by stemness factors, still functions to curb tumor development driven by these oncogenic factors.

Another oncogenic factor that LRIG1 may antagonize is c-Myc, which is overexpressed in >80% of early lesions and represents a major PCa driver through both AR-related and AR-independent mechanisms^53–62^. We show that downregulation of endogenous LRIG1 results in increased c-Myc in some AR^+^, whereas overexpression of LRIG1 reduces c-Myc in some AR^−^, PCa cells. Significantly, transgenic LRIG1 expression suppresses Myc-driven prostate tumorigenesis (Fig. 4). A reciprocal relationship of Myc and Lrig1 has been reported in the mouse epidermis^29,30^ and work is ongoing to determine how LRIG1 might negatively regulate c-Myc in PCa cells.

Yet another mechanism whereby LRIG1 may inhibit prostate tumorigenesis is via antagonizing ERBB signaling^15–23,34,77,78^. Laederich et al.^16^ observed that in co-transfected 293 cells, LRIG1 forms a complex with each of the ERBB receptors independently of ligand binding, which shortens the half-lives of the receptors through ubiquitination. In PC3 cells, LRIG1 inhibits cell-cycle progression stimulated by EGF and neuregulin stimulation^16^. We find that although AR transcriptionally activates *EGFR* and PCa overexpress *ERBB3* mRNA, LRIG1 negatively regulates ERBB/pERBB proteins in PCa cells in a cell type-dependent manner and inducible LRIG1 expression retards Neu-driven PCa growth. The finding that both *LRIG1* and *ERBB3* mRNA levels are elevated and correlate with each other in PCa (Fig. 8) are interesting and intriguing, whose biological significance and underlying mechanisms await experimental explorations.

Altogether, our results suggest a novel conceptual paradigm, in which LRIG1 is feedback induced by, but functions to antagonize, several major PCa-driving oncogenic pathways including AR, c-Myc, and ERBBs (Fig. 10g). Androgens and AR signaling fuel early PCa growth but AR also induces LRIG1 to suppress AR^+^ PCa growth (Fig. 10g). As ERBB ligands and signaling also induce LRIG1^15–21^, our results suggest that both AR and ERBB signaling feedback induce the common inhibitor LRIG1 to curb their oncogenic activities (Fig. 10g). As AR and ERBBs^70–73^, and AR and Myc^53–62^, cross-regulate and reinforce each other to drive prostate oncogenesis and therapy resistance^80,81^, it is tempting to speculate that during PCa development and progression, the host, via producing androgens and ERBB ligands and activating AR and ERBB signaling, respectively, also upregulates LRIG1 in attempt to restrict unchecked oncogenic signaling and retard tumor growth (Fig. 10g). This speculation is partially supported by observations that, although *AR* and *ERBB3* (and in some cases, *EGFR*) mRNA levels are elevated in prostate tumors, at the protein level, there exists a strong inverse correlation between AR and EGFR/pEGFR(pY1173) and a moderate inverse correlation between AR and pERBB3(pY1289), presumably because AR also activates LRIG1 to post-translationally degrades (p)ERBB proteins.

Tissue homeostasis entails a delicate balance between proliferative signaling and anti-proliferative mechanisms, and tumor development ensues when oncogenic signaling overrides tumor-suppressive mechanisms. Rampant hyper-proliferative signaling may cause replicative stress that further promotes tumor evolution and progression. Consequently, developing tumors (and the host) often evolve inhibitory mechanisms to mitigate unchecked oncogenic signaling^82^. The concept of a proliferative vs. anti-proliferative toggle-switch in relation to tumor development is best evidenced by simultaneous activation, in developing (incipient) tumors, of oncogenes such as Myc and Ras and overexpression of tumor suppressors such as cyclin-dependent kinase inhibitors p16, p21, and p27^83–85^. PCa development also involves activation of multiple oncogenic signaling pathways highlighted by AR, c-Myc, ERBBs, and PI3K/AKT^80,81^, but whether there exists a general feedback inhibitory mechanism to antagonize these oncogenic signals remains unclear. Our present study suggests that LRIG1 may well represent such a pleiotropic anti-mitogenic feedback inhibitor (Fig. 10g). Conceptually, this model may help explain persistent overexpression of LRIG1 in PCa and perhaps its continued efforts in neutralizing multiple oncogenic assaults resulting in the overall indolent nature of most prostate tumors. Correlation of LRIG1 expression with better patient survival and tumor-suppressive functions of LRIG1 suggest that this molecule may be of diagnostic and prognostic values in PCa. As LRIG1 inhibits CRPC and displays a therapeutic efficacy in established AR^−^ tumors, our results provide experimental rationales to develop novel LRIG1-based anti-PCa therapeutics.

## Methods

### Scientific premise and rigor of experiments

LRIG1 has been extensively studied and shown to be a tumor suppressor in several other tumor systems and a regulator of stem cell quiescence in the epidermis and small intestine. Studies of LRIG1 in PCa are very limited: A Pubmed search using the term “LRIG1 AND prostate cancer” turned up only 5 references with only one study specifically dedicated to LRIG1 in PCa, which reported conflicting expression patterns of LRIG1 in two cohorts of PCa patients^44^. Hence, the scientific premise of this project is to fill this critical gap of our knowledge by systematically investigating the expression, biological functions, molecular regulation, and mechanisms of action of LRIG1 in both untreated and treatment-failed (i.e., castration-resistant) PCa. All in vitro and in vivo studies were conducted with high scientific rigor. In vitro studies were performed with 3–5 repeat experiments and with triplicate or quadruplicate samples per condition in each experiment. All in vivo xenograft studies were carried out in male mice as we were studying PCa, and each experimental group had sufficient animals to achieve robust statistical power. When feasible and applicable, in vivo experiments were repeated using several different models.

### Cell line authentication and research ethics

This project did not involve Human Subjects research but involved the use of numerous PCa cell lines, xenograft, and genetic mouse models (below). All cell lines were regularly tested to be negative for mycoplasma contamination using the Agilent MycoSensor PCR Assay kit, and authenticated by our institutional CCSG Cell Line Characterization Core via short tandem repeat (STR) analysis. All animal-related (xenograft and genetic model) studies have been approved by the M.D Anderson Cancer Center (MDACC) IACUC (ACUF#00000923-RN00) or the Roswell Park Comprehensive Cancer Center (RPCCC) (animal protocol# 1331 M). All studies using archived human (tumor) specimens such as PCa and CRPC sections have been approved by the Institutional Review Board (IRB STUDY00000079).

### Cells, animals, and reagents

Du145, PC3, PPC-1, LNCaP, VCaP, RWPE-1, and 22Rv1 PCa cells were obtained from ATCC whereas IGR-1 (i.e., IGR-CaP1) cells were initially originated from a primary epithelial prostate cancer^86^. 22Rv1 cells, consistent with the original report^87^, expressed much lower levels of AR protein than LNCaP cells (see Supplementary Fig. 6c). HEK 293T packaging cells were purchased from Clontech. All cell lines were maintained in serum- and antibiotic-containing media as suggested by the providers. LAPC4 and LAPC9 (AD/AI) cells were maintained as xenograft tumors^45–47,49–51^. None of the cell and xenograft lines used in the present study was on the list of the 524 contaminated and misidentified cell lines reported by ICLAC (http://iclac.org/databases/cross-contaminations). Immunodeficient mice, NOD/SCID and NOD/SCID-IL2Rγ^−/−^ (i.e., NSG), initially purchased from the Jackson Laboratory, were produced mostly from our own breeding colonies. Most common laboratory reagents were obtained from Sigma-Aldrich (St. Louis, MO, USA) unless otherwise indicated.

### Immunohistochemistry and quantification

Formalin-fixed paraffin-embedded^46,49,51,69^ (FFPE) tissue sections (4 μm) and tissue microarrays (TMA) were de-paraffinized in xylene and hydrated in graded alcohols to water. Endogenous peroxidase activity was blocked with 3% H_2_O_2_ for 10 min followed by antigen retrieval in 10 mM Citrate Buffer (pH 6.0). After blocking with Biocare Blocking Reagent (Biocare), primary antibodies (Supplementary Table 1) were incubated at appropriate dilutions (generally 1:100–1,1000) for 1–2 h at room temperature. The mouse mAb to LRIG1 (1:100) dilution was most frequently used in IHC studies. Slides were washed in phosphate-buffered saline (PBS) twice (5 min each time) and then incubated in biotinylated anti-rabbit or mouse IgG (Vector Laboratories) at a 1:500 dilution for 30 min at room temperature, followed by streptavidin-conjugated horseradish peroxidase (BioGenex Laboratories Inc., San Ramon, CA) and DAB (BioGenex Laboratories Inc.) development. For semi-quantitative measurement of LRIG1 staining intensity, IHC-stained TMA cores or PCa slides were evaluated^40^ on a four-graded scale: 0 for no or very faint staining, and 1 for weak, 2 for strong, and 3 for intense staining. When the slide/core showed variable staining intensity, the higher staining score was adopted for the whole section. The mean and median staining scores for the same tumor or nonmalignant tissue from each patient were calculated.

### Immunofluorescence staining

For IF staining of PCa cells, 10–30 K cells were seeded on autoclaved glass cover slips, which were put in 12-well plate overnight and then fixed with 4% paraformaldehyde (PFA) solution for 10 min followed by permeabilization with 0.1% Triton for 5–10 min at room temperature. Cells were blocked using background sniper (Biocare Medical, #BS966H) for 15 min at room temperature followed by incubation with primary antibodies (Supplementary Table 1; the mouse mAb against LRIG1 was used at 1:100) for 2 h and appropriate conjugated secondary antibodies and DAPI counterstaining for 1 h at room temperature. Coverslips were mounted on slides in mounting medium (Vector Laboratories, Cat. #H-1400) and images taken on an Olympus Inverted epifluorescence microscope.

For IF staining of paraffin-embedded tissue, sections (4 μm) were de-waxed in 60 °C incubator for 10–15 min, and deparaffinized and dehydrated through Xylene for 30 min, then hydrated through serial decreasing concentrations of ethanol (95%, 90%, 85%, 70%, and 50%, 3 min twice at each percentage) washes followed by antigen retrieval in boiling target retrieval solution (S1699, Dako) for 40 min. Slides were blocked by background sniper and incubated with primary antibodies at 4 °C overnight, followed by incubation with secondary antibodies and DAPI. For IF staining of OCT embedded tissues, sections were air dried at room temperature for 5 min and fixed in 4% PFA for 15 min at room temperature, and then were blocked and incubated with primary and secondary antibodies as in staining of FFPE sections.

### Tissue microarrays and whole-mount CRPC sections

Three TMAs from PCa patient samples (designated TMA 75, TMA 115 and TMA 124; see Fig. 1f and Supplementary Fig. 4a–e), one CRPC TMA from CRPC patients (*n* = 40 cores with 2 cores per patient) and six whole-mount (WM) CRPC patient slides (Fig. 5a, Supplementary Fig. 13d) were used in this study. TMA 75 (*n* = 300 cores) was created from 75 patients with matched tumor and normal tissues (2 normal and 2 tumor cores per patient); and TMA 115 (*n* = 690 cores) and TMA 124 (*n* = 744 cores) were similarly created from 115 and 124 patients, respectively, with matched tumor and normal tissue (3 tumor and 3 tumor cores per patient). All TMAs and CRPC WM slides were kindly provided by Dr. Jiaoti Huang (Duke University). FFPE sections were cut from these samples and used for IHC of LRIG1 and AR. Relevant information on TMAs and on primary patient tumor (HPCa) samples used in this study was summarized in Supplementary Table 2.

### Aperio Scanscope analysis and quantification

HE or IHC-stained glass slides containing sections of patient tumor, TMA and WM sections were scanned via an Aperio ScanScope imaging platform (Aperio Technologies, Vista, CA) with trainable GENIE morphometric software that permits morphometric quantification of scanned images^46,47,50,51^.

### Plasmids, vectors, viral production, and cell infection

The major plasmids and vectors used in this study are summarized in Supplementary Fig. 5. Briefly, the pLVX-IRES-zsGreen (Cat. #632187) and pLVX-TetOne-puro (Cat. #631849) vectors were purchased from Clontech while the GIPZ-shRNA-encoding lentiviral vectors were bought from GE Dharmacon. For construction of LRIG1 overexpressing lentiviral vectors, full-length human LRIG1 cDNA was cloned from pBABE-LRIG1-Puro (courtesy of Dr. Yosef Yarden, Bar llan University, Israel)^15^, and inserted into the multiple cloning site of pLVX-IRES-zsGreen or pLVX-TetOne-puro to generate the pLVX-LRIG1 or pLVX-LRIG1-Puro lentiviral vectors. For rat-Neu* overexpression vector, full-length rat Neu* sequence form pCMV-ratNeu* (provided by Dr. Argiris Efstratiadis)^75^, was subcloned into the multiple cloning site of pLVX-IRES-zsGreen vector. Lenti-X HTX packaging system (Clontech, Cat. #631251) was used for pLVX constructs and Lenti-X Packaging Single Shots (Clontech, Cat. #631275) was used for pLVX-TetOne-Puro inducible vectors whereas Trans-Lentiviral shRNA Packaging Kit (GE Dharmacon, Cat #TLP5912) was used for GIPZ-shRNA packaging.

For experiments, we produced retroviruses and lentiviruses^45–47,49–51^ in 293T packaging cells (Clontech), and the titer was estimated by GFP positivity. PCa cells were infected at a multiplicity of infection (MOI) of 5–10 for 48–72 h at 37 °C in the presence of 8 μg/ml polybrene. The overexpressing and knockdown effects on target molecules were assessed by qRT-PCR and/or western blotting.

### Clonal, clonogenic, and sphere-formation assays

For clonal assays, cells were seeded at low density (100–300 cells/well) in a 6-well plate and allowed to grow until visible clones appeared, and holoclones were counted within 2 weeks. For clonogenic assays, 500–1000 cells were mixed with Matrigel (BD Bioscience) at 1:1 ratio, and colonies were enumerated in ~2 weeks. For sphere-formation assays, 1000–2000 single cells were plated in ultra-low attachment (ULA) plates, and spheres were counted within 1–2 weeks. For both clonogenic and sphere-formation assays, we used the prostate epithelial basal medium (PrEBM) supplemented with B27 (Invitrogen), 20 ng/ml EGF, 20 ng/ml bFGF, and 4 μg/ml insulin. For these assays, 3–6 wells were plated for each cell density and the experiment was repeated 2–3 times.

### Cell viability and BrdU incorporation assays

For cell viability determination, 500 cells were plated in triplicate in 6-well plates. After 1–5 days, viable cell numbers were counted via trypan blue exclusion assays. For BrdU incorporation assays, cells plated on coverslips the day before were pulsed for 4 h with 10 μM BrdU (B5002, Sigma), fixed in 4% paraformaldehyde and incubated with mouse anti-human BrdU (B2531, Sigma) antibody at 4 °C overnight. After thorough washing, coverslips were incubated at room temperature for 1 h with Alexa Flour 594-conjugated goat anti-mouse IgG (1:500). Coverslips were then counterstained with DAPI (1:1000) and mounted with 10 μl Gold Antifade Reagent (936590, Prolong). Images were acquired under microscope (Nikon, Eclipse E800). A minimum of 1000 cells was counted for each condition.

### Xenograft tumor and therapeutic experiments

Cultured PCa cells (e.g., Du145), or tumor cells freshly purified out from xenografts (e.g., LAPC9) were mixed, at different numbers, with Matrigel at 1:1 ratio and injected subcutaneously (s.c) into the flanks of NOD/SCID or NSG mice depending on the model^45–47^. For Doxycycline (DOX) controlled Tet system (pLVX-TetOne-LRIG1-Puro; Supplementary Fig. 5d), mice were randomly divided into 2 groups after the tumors became palpable. DOX was delivered in standard chow food (200 mg/kg diet) in the experimental group till killed. Tumor volumes were measured 3 times per week. At the endpoint, tumors were harvested and various parameters were recorded, including tumor images, tumor weight, incidence, latency, and tumor-initiating frequency (TIF) (http://bioinf.wehi.edu.au/software/elda/)^45–47^.

### Establishing matched AD and AI PCa xenografts

We have recently detailed the establishment of the matched AD/AI pairs of human PCa xenografts in immunodeficient mice^51^. Briefly, PCa models (LNCaP, VCaP, LAPC4, and LAPC9) were routinely maintained in hormone-intact immunodeficient mice as AD xenograft tumors. To establish the AI lines, parental AD tumor cells were purified and mixed with Matrigel, then injected subcutaneously and serially passaged in surgically castrated male mice. The LAPC4 and LAPC9 xenograft lines were maintained and passaged in NOD/SCID mice, whereas LNCaP and VCaP xenograft lines were passaged in NSG mice.

### Purification of human PCa cells from xenograft tumors

Xenograft tumors were harvested and chopped into small pieces (~1 mm^3^), washed by phosphate-buffered saline (PBS) and digested with 1x Accumax (Innovative Cell Technologies) for 30 min at room temperature at rotating conditions. Single cell suspensions were then prepared via filtering through a pre-wetted 40-μm strainer followed by Histopague-1077 (Sigma) gradient purification to deplete debris and dead cells. Finally, the cell mixture was incubated with a biotinylated mouse H-2K[d] antibody (SF1-11, BD Biosciences) for 30 min at 4 °C to remove mouse stromal cells via MACS (Miltenyi Biotec).

### Generation and crossing of *ARR2PB*-LRIG1 transgenic mice

The ~3.1-kb human LRIG1 cDNA fragment was subcloned into the multiple cloning site of pPB.197 vector that contains the *ARR2PB* promoter, and the construct (*ARR2PB*-LRIG1) was used to generate *ARR2PB*-LRIG1 transgenic mice with FVB background (as previously described^88,89^) at the Transgenic Core at Science Park, Departmental of Epigenetics and Molecular Carcinogenesis, the M.D Anderson Cancer Center. For genotyping, mouse tail snips were collected and lysed in the solution containing 100 mM Tris-HCl (pH 8.0), 200 mM NaCl, 5 mM EDTA, 0.5% SDS, and 0.2 mg/ml proteinase K at 55 °C overnight. Two founder lines of LRIG1 transgenic mice (Tg1 and Tg2) were identified by PCR genotyping and western blotting. LRIG1 Tg animals were crossed with Hi-Myc or TRAMP animals to produce LRIG1;Myc or LRIG1;TRAMP double transgenic (dTg) animals. Hi-Myc animals were initially obtained from Dr. J DiGiovanni lab. TRAMP mice were purchased from the RPCCC animal facility. Genotyping primers for LRIG1 Tg, Hi-Myc, and TRAMP animals were listed in the Supplementary Table 3. All procedures involving the usage of transgenic mice were approved by the MDACC and RPCCC Institutional Animal Care and Use Committee (IACUC).

### Prostate isolation and microdissection

In brief, after killing mice, the prostates were surgically removed along with the urogenital tract, then immediately placed in ice-cold PBS. Microdissection was processed under a dissection microscope to remove fat and connective tissues. The isolated whole-mount prostates were photographed by Nikon digital camera (DXM1200F), and then put into O.C.T. compound (Tissue-Tek, Cat. # 4583) or 10% formalin for further histological analysis.

### RNA isolation and quantitative RT-PCR analysis

Total RNA was isolated from cells, mouse prostates or human xenograft tumors using RNeasy mini kit (Qiagen) following the manufacturer’s protocol. For qRT-PCR, first-strand cDNA synthesis from total RNA was carried out using SuperScript^®^III Frist-Strand synthesis kit (Life Technology), and the resulting cDNA was then incubated with iTaq Universal SYBR Green Supermix (BIO-RAD) and the respective mRNA levels were analyzed by qRT-PCR in ABI Prism 7900HT Real-Time PCR detection system by normalizing to human GAPDH or mouse Gapdh. qRT-PCR primers were listed in Supplementary Table 3.

### Western blotting and quantitative Wes immunoassays

Whole cell lysates (WCL) from cells or tumor tissues were prepared in complete RIPA buffer (150 mM NaCl, 50 mM Tris-HCl, pH 7.5, 10 mM EDTA, 1% Nonidet P-40, 0.5% sodium deoxycholate, 0.5% Triton X-100) containing protease inhibitor mixture, and the protein concentrations were measured via MicroBCA kit (Pierce). In all, 40–80 µg proteins were analyzed by 4–12.5% SDS-PAGE and gels were transferred to the immobilon-P transfer membrane (Millipore). The membrane was blocked with 5% non-fat milk in TBST (10 mM Tris-HCl, 150 mM NaCl, and 0.1% Tween-20) for 1 h at RT, and then incubated with primary antibody (Supplementary Table 1) overnight at 4 °C. Most primary antibodies used in western blot were diluted at 1:1000. Membranes were washed three times (10 min/time) with TBST buffer, followed by incubation with respective secondary antibodies at room temperature for 1 h. Finally, western blotting (WB) was performed with ECL Plus WB detection reagent (PerkinElmer). In some experiments, protein bands were scanned by densitometry and levels normalized to GAPDH, and band intensities quantified by Image J.

In some experiments (e.g., Supplementary Figs. 6c,  7a,  9h, and Supplementary Fig. 13g), WCL was analyzed for protein levels using the Wes system (www.proteinsimple.com), in which size-based Simple Western Wes immunoassays take place in capillaries, separated by size as they migrate through stacking and separation matrix. The separated proteins are immobilized to the capillary wall via a proprietary, photoactivated capture chemistry. Target proteins are identified using a primary antibody and immunoprobed using an HRP-conjugated secondary antibody and chemiluminescent substrate. The resulting chemiluminescent signal is displayed as traditional virtual blot-like image and electropherogram. Quantitative results such as molecular weight, signal intensity (area), % area, and signal-to-noise for each immunodetected protein are presented in the results table automatically.

### siRNA transfection experiments

To validate the effects of lentiviral-mediated LRIG1 knockdown, we obtained the ON-TARGETplus Human LRIG1-targeting siRNA (26018) from Thermo Scientific Dharmacon, as both a set of 4 individual siRNAs (cat. # LQ-013940-00-0002: 5′-GAACAGGAUUCGGCU GAUA-3′; 5′-UGUAAGAGCAUUCAAGCUA-3′; 5′-GGCAAGGACAUCCGGUUUA-3′; and 5′-CGAGAUUU CGGGCACAAUA-3′) and the SMARTpool (cat. # L-013940-00-0005) containing the mixture of 4 individual siRNAs. A ON-TARGETplus Non-targeting (NT) pool of siRNAs (cat. # D-001810-10-20) was also purchased as control, consisting of 4 individual siRNAs (targeting sequences: 5′-UGGU UUACAUGUCGUCUAA-3′; 5′-UGGUUUACAUGUUGUGUGA-3′; 5′-UGGUUUACAUGUUUUCUGA-3′; and 5′-UGGUUUACAUGUUUUCCUA-3′). LNCaP cells were plated in 6-well plates (2 × 10^5^ cells/well) in duplicate in RPMI-10% FBS. On the following day, cells were transfected of either siRNA SMARTpool or individual oligos (10 nM final concentrations) using Lipofectamine RNAiMAX^®^ transfection reagent (Invitrogen) according to the manufacturer’s protocol. For viability (cell growth) assays, LNCaP cells were plated in 24-well plates (5,000 cells/well) in triplicate in RPMI 10% FBS. Following the transfection with siRNA, live cell numbers were quantified by Trypan blue assays on daily basis for up to 8 days. For clonal assays, LNCaP cells were plated in 6-well plates (50,000 cells/well) in triplicate. Following siRNA transfection, the number of clones was determined on day 8.

In another set of experiments, PCa cells (LNCaP or VCaP) were cultured in 6-well plates in RPMI or DMEM with 10% FBS. On the following day, cells were transfected with 10 nM AR-targeting siRNA pool (Thermo Scientific Dharmacon, ON-Targetplus SMARTpool, human AR, L-003400-00) or the control nontargeting siRNA (siCtrl) using Lipofectamine RNAiMAX transfection reagent (Invitrogen) at 4 μl/well according to the manufacturer’s protocol. Cell pellets were collected after 48 or 72 h.

### R1881 and DHT treatment

LNCaP or VCaP cells were suspended in phenol red-free medium and 10% CDSS (charcoal dextran-stripped serum). After 48 h, cells were treated with 0.1 nM R1881 or EtOH for 2–120 h before harvest for qRT-PCR and western blot analyses. Medium with fresh R1881 was replenished every 48 h. For DHT treatment, LNCaP cells were suspended in phenol red-free medium and 10% CDSS. After 48 h, cells were treated with 0–1000 nM DHT or EtOH for 48–72 h before harvest for qRT-PCR, Western blot, and IF analyses. Medium with fresh DHT was replenished every 48 h.

### Chromatin immunoprecipitation and ChIP-qPCR

Public ChIP-Seq data were downloaded from GEO (Gene Expression Omnibus) website (http://www.ncbi.nlm.nih.gov/geo/). For AR ChIP-Seq in LNCaP cells (AD: GEO# GSM699631, and AI: GEO# GSM699630), raw sequences were downloaded and mapped to hg38 using bowtie. UCSC genome browser (http://genome.ucsc.edu/) was used for AR peak calling and AR and FOXA motif discovery. For de novo motif analysis, peaks were called via MACS using a stringent *p*-value cutoff.

ChIP assay was performed using chromatin purified from LNCaP and VCaP cultured cells, or LAPC9 and LNCaP AD/AI xenograft cells. Cells were crosslinked with 1% formaldehyde for 10 min at room temperature, and quenched with 125 mM glycine for 5 min with gentle rocking. Cells were then washed twice in PBS. In all, 5 × 10^6^ cells were collected in 1 ml Farnham lysis buffer (5 mM PIPES pH 8.0/85 mM KCl/0.5% NP-40) supplemented with protease inhibitors (1 μM protease inhibitor cocktail and 1 mM PMSF), and centrifuged at 4000 × *g* for 5 min at 4 °C. Cell pellets were washed with 10 ml of lysis buffer, followed by centrifugation. Resulting nuclear pellets were re-suspended in 200 μl of SDS lysis buffer with protease inhibitors. Chromatin was sonicated until the DNA fragments were in the range of 200–500 bp, following the manufacturer’s protocol of ChIP Assay Kit (Cat. #17-295, Millipore). In each ChIP reaction, 2 μg of primary antibodies (anti-AR Ab74272 and anti-H3K27ac Ab4729) or corresponding ChIP grade control IgG were used. DNA was purified following the manufacturer’s instruction of the Qiagen PCR purification kit and eluted in 50 μl of H_2_O, and 1 μl of the eluted DNA used for ChIP-qPCR.

### Dual-luciferase assays and mutagenesis assays

The fragment containing the predicted ABS1 (100 bp) for AR in the *LRIG1* genomic region was amplified by PCR from genomic DNA isolated from LNCaP cells. The PCR-derived ABS1 fragment was cloned upstream of the firefly luciferase gene in the pGL4.23-REPORT vector (Promega, E841A) to obtain LRIG1-ABS1-WT. To construct mutant vectors, putative AR binding site in LRIG1 ABS1 was mutated using Quick-Change Site-Directed Mutagenesis Kit (Stratagene). The insert was sequenced to verify the mutation. Primers used in these experiments were presented in Supplementary Table 3.

For luciferase assays, LNCaP cells were plated in 12-well plates and cultured in phenol red-free RPMI media with 10% FBS or CDSS for 48 h. Cells were then transfected with 5 μg pREPORTER or vectors containing wild-type or mutant LRIG1 ABS1, together with 20 ng pRenilla expressing vector (transfection control) using 4 μl Lipofectamine 2000 transfection reagent (Invitrogen). In total, 48 h later, cells in each well were treated with 100 nM DHT for 6 h or 24 h, and the luciferase activities were then measured using Dual Luciferase Reporter Assay Kit (Promega) on a Gen-Probe chemiluminometer.

### Bioinformatics analysis

Oncomine (www.oncomine.com; Compendia Bioscience) datasets of PCa were analyzed to determine *LRIG1*, *AR*, and *ERBB* family mRNA expression levels and compared with normal tissues and in tumors of different grade, and also to determine the co-expression relationship between *LRIG1*, *AR,* and *ERBB* family members. 17 PCa datasets containing *LRIG1* mRNA expression data and DNA copy number information was analyzed in detail for correlations with patient survival rate, *P* value, fold change, and gene rank values extracted. We also applied differentiation analysis to interrogate the gene expression in each sample. Concept analysis was also performed in some cases for a cohort of genes. For survival analysis, Kaplan–Meier survival plots were generated using the survival package in R. Detailed information for these analyses has been described^47^. In brief, we obtained individual normalized gene expression, survival time, and survival status from individual datasets in Oncomine. We ranked the samples according to the gene expression and performed survdiff to test the statistics p-value.

For TCGA data analysis, we obtained TCGA level-3 data from TCGA data portal (https://tcga-data.nci.nih.gov). Box plot was drawn by boxplot in R. We performed the *t*-test for normal and tumor tissue comparison and one-way ANOVA for examination of expression levels among different Gleason scores. To determine the linear relationship between *LRIG1, AR*, and *ERBB* family members, we calculated Pearson correlation coefficient for linear regression by lm function and drew the scatter plot with regression line by plot and abline in R. LRIG1 mRNA levels in normal human tissues (Supplementary Fig. 1a) were extracted from the GTEx (Genotype-Tissue Expression) data portal.

### The Cancer Proteome Atlas analysis

TCPA analysis was performed through the website (tcpaportal.org) developed by the Department of Systems Biology and Bioinformatics and Computational Biology at The University of Texas M.D Anderson Cancer Center. The data release used in our studies contained 8,167 tumor samples in total, mainly consisting of TCGA tumor tissue sample sets^76^.

### Statistical analysis

Unpaired two-tailed Student’s *t*-test was used to compare significance in cell numbers (viability), % of Ki-67^+^, cleaved Lamin^+^ and BrdU^+^ cells, cloning and sphere-forming efficiencies, tumor weights, knockdown efficiency, mRNA levels of multiple genes and other related parameters. We employed Fishers Exact Test and χ^2^ test to compare tumor incidence, and Log-Rank test to analyze the survival curves. Most results were presented as the mean ± S.D with a *P* value < 0.05 considered statistically significant.

### Reporting summary

Further information on research design is available in the Nature Research Reporting Summary linked to this article.

## Supplementary information

Supplementary Information

Reporting Summary

## Data Availability

All relevant data are available from the authors. This project did not involve new in-house RNA-seq experiments or data; therefore, there are no mandated accession codes. However, we extensively exploited publicly available cDNA microarray datasets in Oncomine and RNA-seq datasets in TCGA and GTEx (see Methods). In addition, two datasets (GSM699631 and GSM699630) were used to determine AR binding to the *LRIG1* locus in AD and AI LNCaP cells, respectively (Fig. 7), and the Rajan dataset (GSE48403; ref. ^67^) was used to compare the *LRIG1* mRNA levels in 7 PCa patients before and after ADT treatment (Supplementary Fig. 14d). The source data underlying Figs. 1f, g; 2a, b, d, e; 3a, b, c, e, f;  4c, d, f, h; 5c, d, g, h; 6a–e; 7 (gel images); 9 (gel images); and 10 (gel images); Supplementary Figs. 6a–c; 7a–i; 8;  9a–i; 10; 12d, e; 13e–g; and 14 have been provided as a Source Data file. There are no restrictions to data availability.

## Source Data

Source Data

## Code availability

Three custom codes, developed for generating box plots from the TCGA PRAD dataset (Fig. 1c) and conducting patient survival analysis (Fig. 1d; Supplementary Fig. 3b), were provided in the online resources associate with this project. There is no restriction to access.
