# Author Correction: LRIG1 is a pleiotropic androgen receptor-regulated feedback tumor suppressor in prostate cancer

**DOI:** 10.1038/s41467-020-16615-9

**Published:** 2020-06-04

**Authors:** Qiuhui Li, Bigang Liu, Hsueh-Ping Chao, Yibing Ji, Yue Lu, Rashid Mehmood, Collene Jeter, Taiping Chen, John R. Moore, Wenqian Li, Can Liu, Kiera Rycaj, Amanda Tracz, Jason Kirk, Tammy Calhoun-Davis, Jie Xiong, Qu Deng, Jiaoti Huang, Barbara A. Foster, Abhiram Gokhale, Xin Chen, Dean G. Tang

**Affiliations:** 1 Department of Pharmacology and Therapeutics, Roswell Park Comprehensive Cancer Center, Buffalo, NY 14263 USA; 2 State Key Laboratory Breeding Base of Basic Science of Stomatology (Hubei-MOST) and Key Laboratory for Oral Biomedicine of Ministry of Education (KLOBM), School and Hospital of Stomatology, Wuhan University, 430079 Wuhan, China; 3 Department of Epigenetics and Molecular Carcinogenesis, University of Texas M.D. Anderson Cancer Center, Science Park, Smithville, TX 78957 USA; 4 Department of Pathology, Duke University of School of Medicine, Durham, NC 27710 USA; 5 Department of Oncology, Tongji Hospital, Tongji Medical School, Huazhong University of Science and Technology (HUST), 430030 Wuhan, China; 6 Cancer Stem Cell Institute, Research Center for Translational Medicine, East Hospital, Tongji University School of Medicine, 200120 Shanghai, China

**Keywords:** Cancer, Tumour-suppressor proteins, Urological cancer, Prostate cancer, Cell biology

Correction to: *Nature Communications* 10.1038/s41467-019-13532-4, published online 2 December 2019.

The original version of this Article contained an error in the third subheading of the “Results” section, which incorrectly read “Knocking down endogenous LRIG1 inhibits AR^+^LRIG1^+^ PCa”. The correct version should have been “promotes” in place of “inhibits”.

This has been corrected in both the PDF and HTML versions of the Article.

