# Supplementary material for: LRIG1 is a pleiotropic androgen receptor-regulated feedback tumor suppressor in prostate cancer
Source: Nat Commun. 2019 Dec 2;10:5494. doi: 10.1038/s41467-019-13532-4 (PMC6889295; doi:10.1038/s41467-019-13532-4)
Supplement: Supplementary file 1 — Supplementary Information [file 41467_2019_13532_MOESM1_ESM.pdf]

Supplementary Information:

LRIG1 is a pleiotropic androgen receptor-regulated feedback tumor suppressor in prostate cancer

Li Q et al.,

The Supplementary Information contains:

16 Supplementary Figures
3 Supplementary Tables

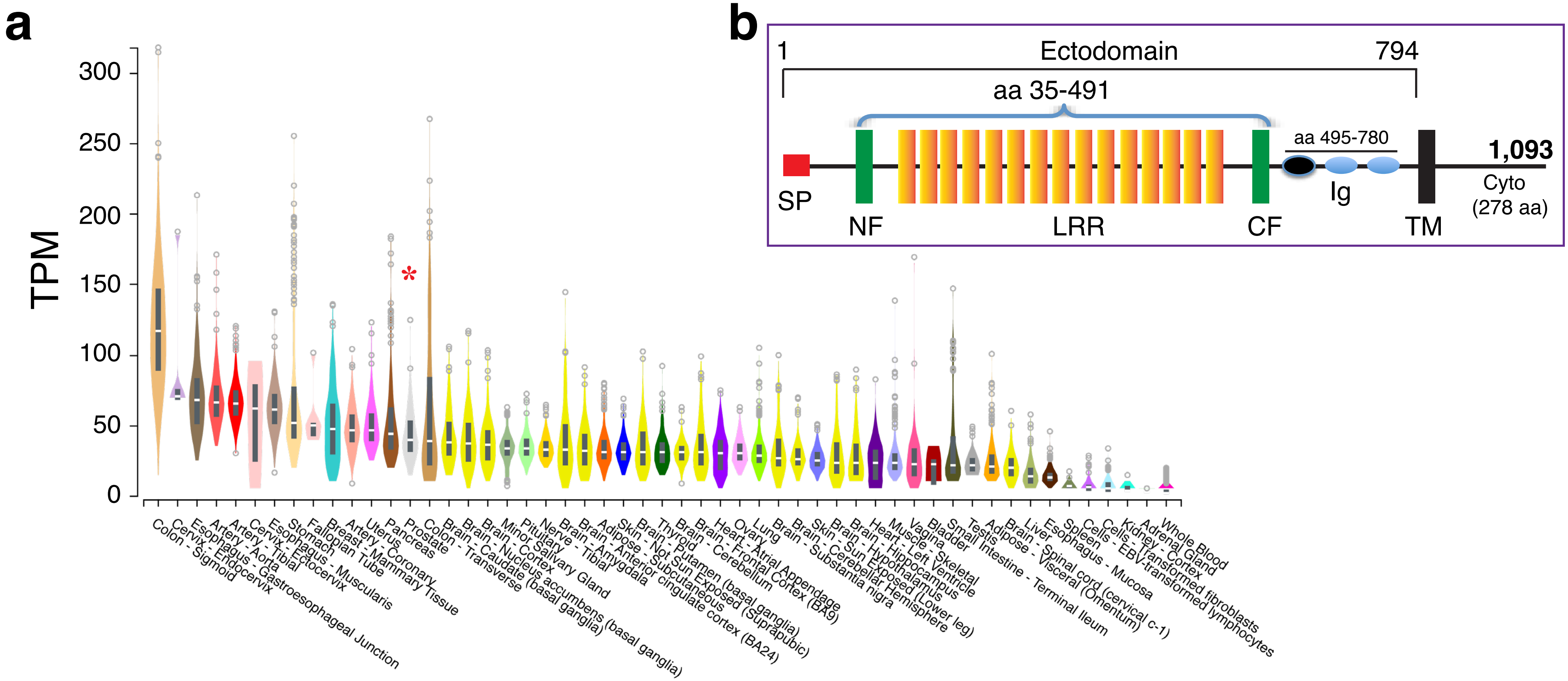

c

**Company (Cat. #)**	**Immunogen**	**Specificity**	**Applications**
**Abcam** Rb pAb (ab36707)	C-ter	H & M	WB/IHC
**Cell Signaling** Rb pAb (#12752)	C-ter	H	WB/IP
**R&D** goat pAb (AF3688)	Ala37-Thr794	M & H	flow/IF/WB [~140 & 70kDa]
**R&D** sheep pAb (AF7498)	Ala35-Ser779	H	flow/IHC
**Santa Cruz** goat pAb (sc-50076, P-16)	ECD of hLRIG1	M & H	IHC
**Sigma** mouse mAb (SAB4200445)	N-ter	H	IHC/IF/WB [143 and 110 kDa]

Supplementary Figure 1. ***LRIG1*** **mRNA expression in GTEx, LRIG1 protein domain structure and antibodies used in this study.**
(**a**) *LRIG1* mRNA levels (TPM; Transcript per Million) in more than 50 human tissues retrieved from GTEx (ENSG00000144749.9). Tissues from different parts of the brain are labeled in yellow. The prostate is indicated with a red asterisk.
(**b**) Protein domain structure of LRIG1. LRIG1 is a transmembrane (TM) protein of 1,093 amino acids (aa) with a signal peptide (SP), cysteine N-flanking domain (NF), 15 leucine-rich repeats (LRR), cysteine C-flanking domain (CF), 3 Ig-like domains (blue, C2-type), a TM domain, and cytoplasmic tail. The LRRs and ECD are involved in EGFR (ERBB) binding. The mature protein (after SP removal) is 1,059 aa and the ectodomain(ECD) contains aa 1-794. Human LRIG1 and mouse Lrig1 are most similar (~90%) over aa 35-779.
(**c**) Anti-LRIG1 antibodies used in this study. The first 2 antibodies were raised against C-ter cytoplasmic region and the other 4 antibodies were all against regions in the N-ter extracellular domain. Polyclonal antibodies (pAb) against the N-ter (aa 1-151) have been reported to detect LRIG1 as 143-kDa and 134-kDa protein species, and the 143 kDa band could be cleaved into ~110-kDa and 32-kDa fragments. Also, ECD can undergo proteolysis generating ~100-110 and 55-60 kDa soluble fragments. In this study, the Sigma mouse monolconal antibody (mAb) was most frequently used in our IHC and WB analysis. H, human; M, mouse.

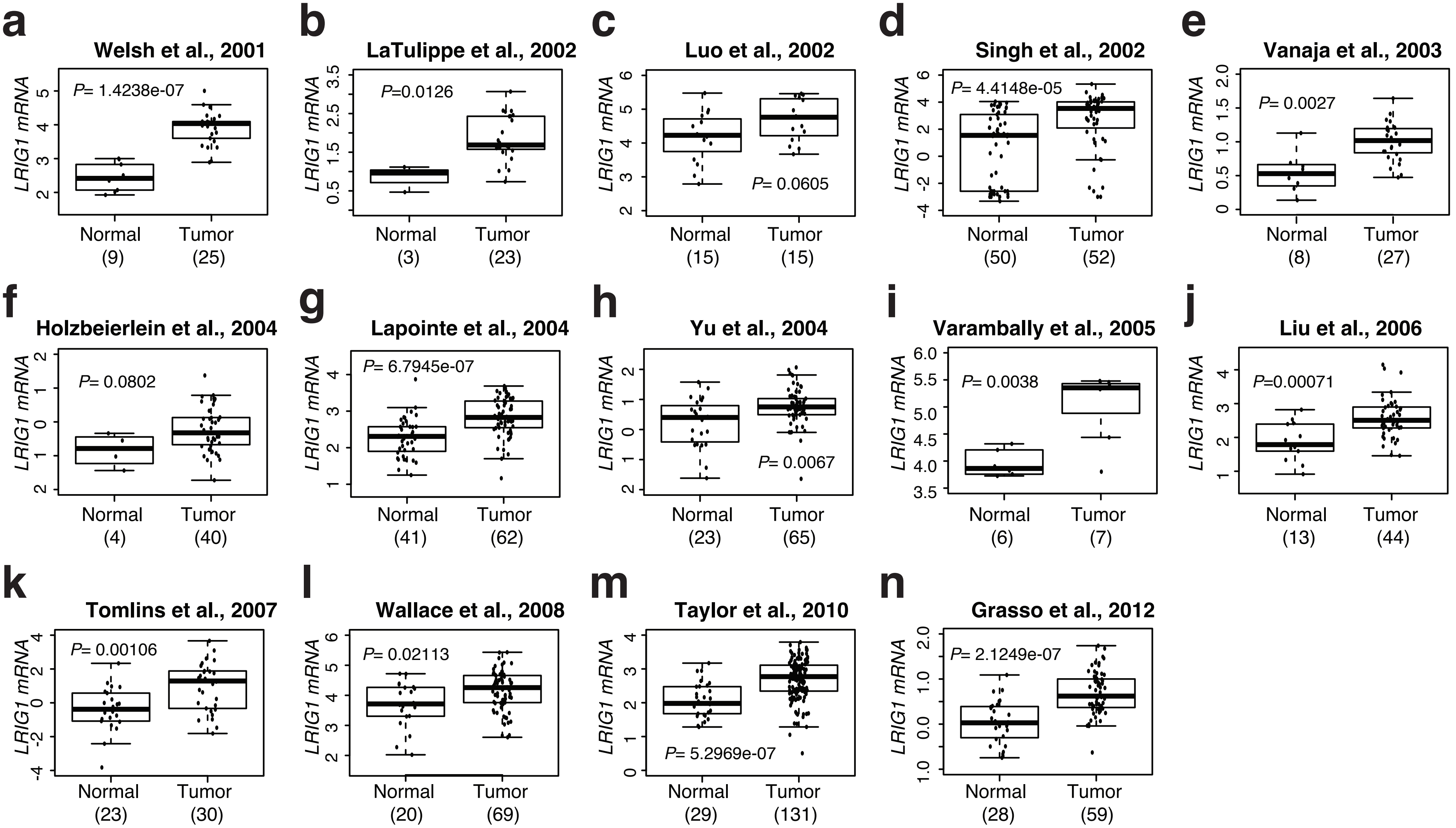

Supplementary Figure 2. LRIG1 *mRNA* is up-regulated in PCa vs. normal prostate tissues.
LRIG1 mRNA levels are up-regulated in prostate tumor tissues compared with the matched normal tissues in the 14 Oncomine datasets (panels a - n) listed chronologically (2001 to 2012). Relative *LRIG1* mRNA levels in tumors vs. normal prostate tissues (n indicated below) are presented in box plots, in which the center lines show the median values, box edges are the first and third quartiles, and the whiskers represent the maximum and mimimum values. Y-axis values represent the normalized RSEM (RNA-Seq by Expectation Maximization). The *P*-values are determined by two-tailed paried Student's *t*-test.

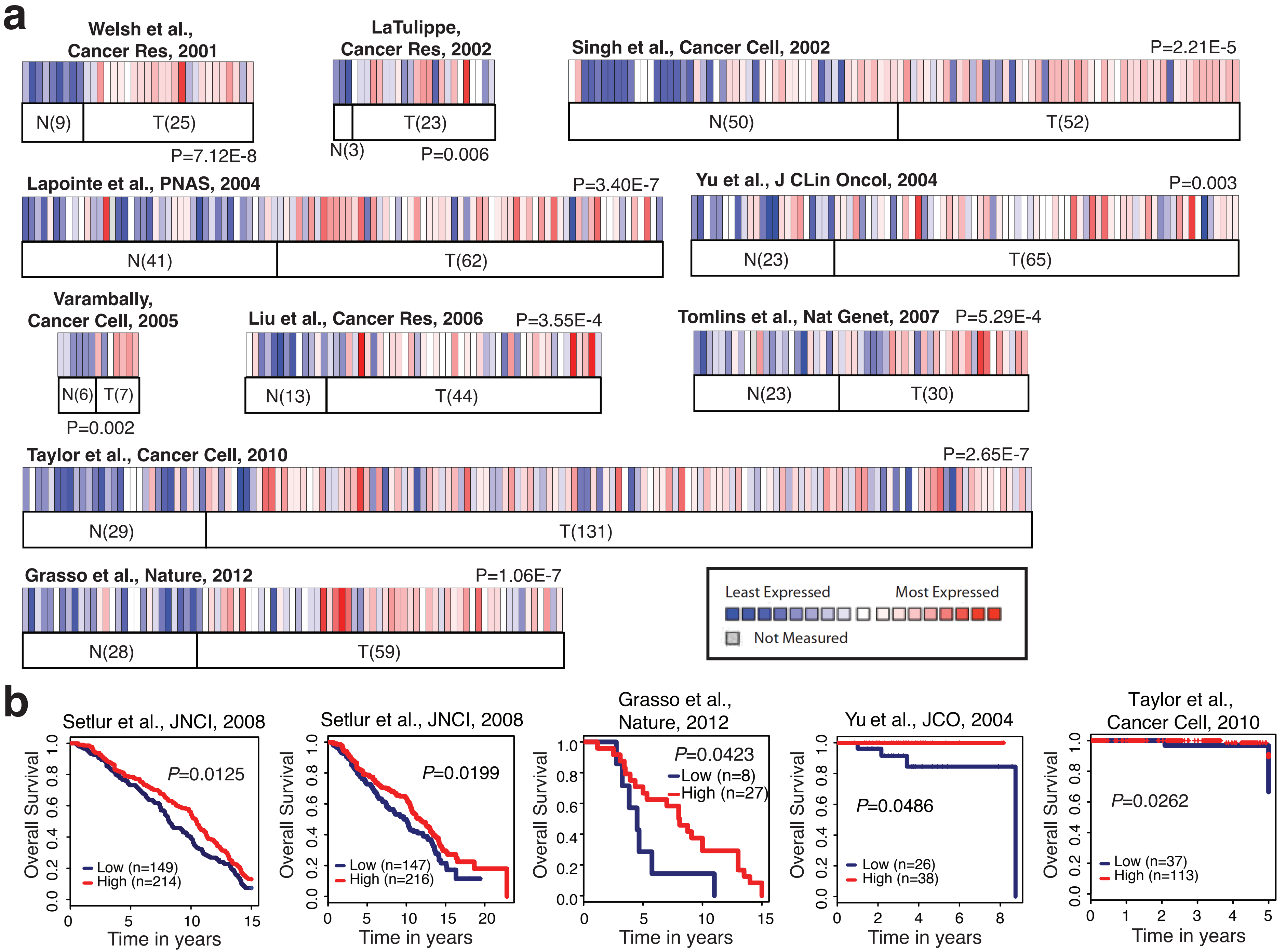

Supplementary Figure 3. *LRIG1* mRNA expression is upregulated in prostate tumors and correlates with better patient survival. (**a**) Heat map of *LRIG1* mRNA levels in patient prostate tumors (T) compared to the matched normal/benign (N) tissues in 10 representative Oncomine datasets. Patient numbers and *P* value are indicated. (**b**) High *LRIG1* mRNA levels correlate with better patient overall survival (OS). Shown are Kaplan-Meier survival curves of patients from 4 Oncomine datasets. For Setlur dataset, we presented OS of 10 (Fig. 1g) and 15 and 20 years (left 2 panels).

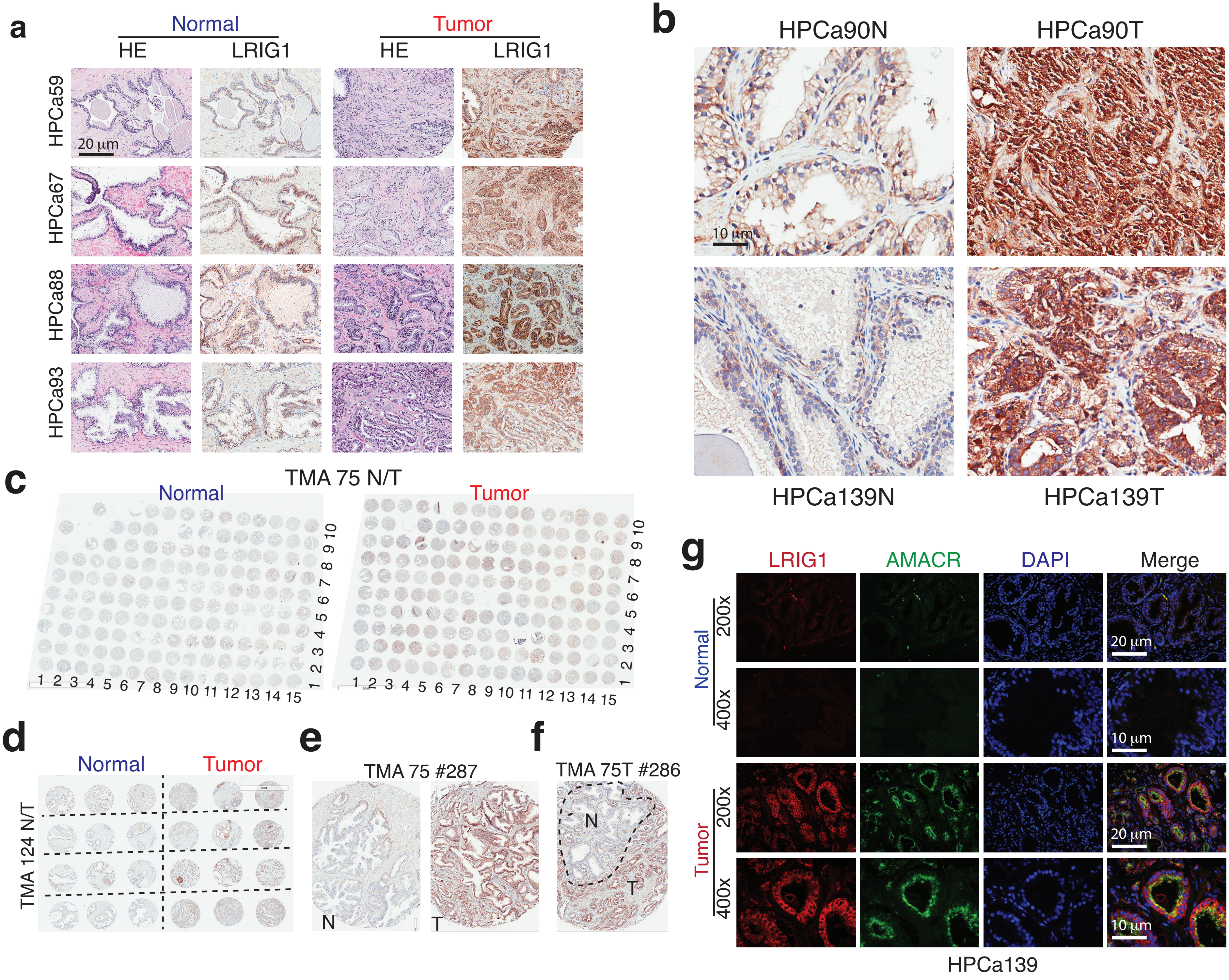

Supplementary Figure 4

Supplementary Figure 4. LRIG1 protein is up-regulated in untreated human PCa.

(**a**) Representative HE and LRIG1 IHC images in matched WM normal/benign and tumor areas in 4 pairs of HPCa samples (i.e., HPCa59, 67, 88 and 93). Bar = 20 μm for all panels and the original magnifications for all images were 200X.

(**b**) Zoom-in images of LRIG1 IHC staining in HPCa90 and HPCa139 tumors (T) and corresponding uninvolved benign/normal (N) tissues. Note both elevated levels and prominent cytoplasmic distribution of LRIG1 in tumor samples (in comparison to low levels and membrane distribution of LRIG1 in normal tissues). Bar = 10 μm for all panels and the original magnifications for all images were 400X.

(**c-f**) Aperio Scanscope images of LRIG1 IHC staining in TMAs. (**c**) The entire TMA 75 N/T was stained for LRIG1 in 75 Gleason 7 tumors (right) and matched normal tissues (left). Each sample has 2 cores. (**d**) Four representative matched prostate normal (N; left) and tumor (T, right) tissues in the TMA 124 N/T were stained for LRIG1. Each sample has 3 cores. (**e**) LRIG1 IHC images of the normal (N) and tumor (T) cores in patient #287 from TMA 75 N/T were shown. (**f)** An LRIG1 IHC image in patient #286 form TMA 75 N/T was presented. Note much higher LRIG1 in T than N areas in the same patient sample.

(**g**) Representative immunofluorescent (IF) images of LRIG1 and AMACR (alpha-methylacyl-CoA racemase) staining in HPCa139. Tumor areas revealed by PCa biomarker AMACR (green) also expressed high levels of LRIG1 (red). Original magnifications are indicated on the left and scale cars on the right. LRIG1 IHC and IF in all experiments presented here were carried out using the Sigma mAb against LRIG1.

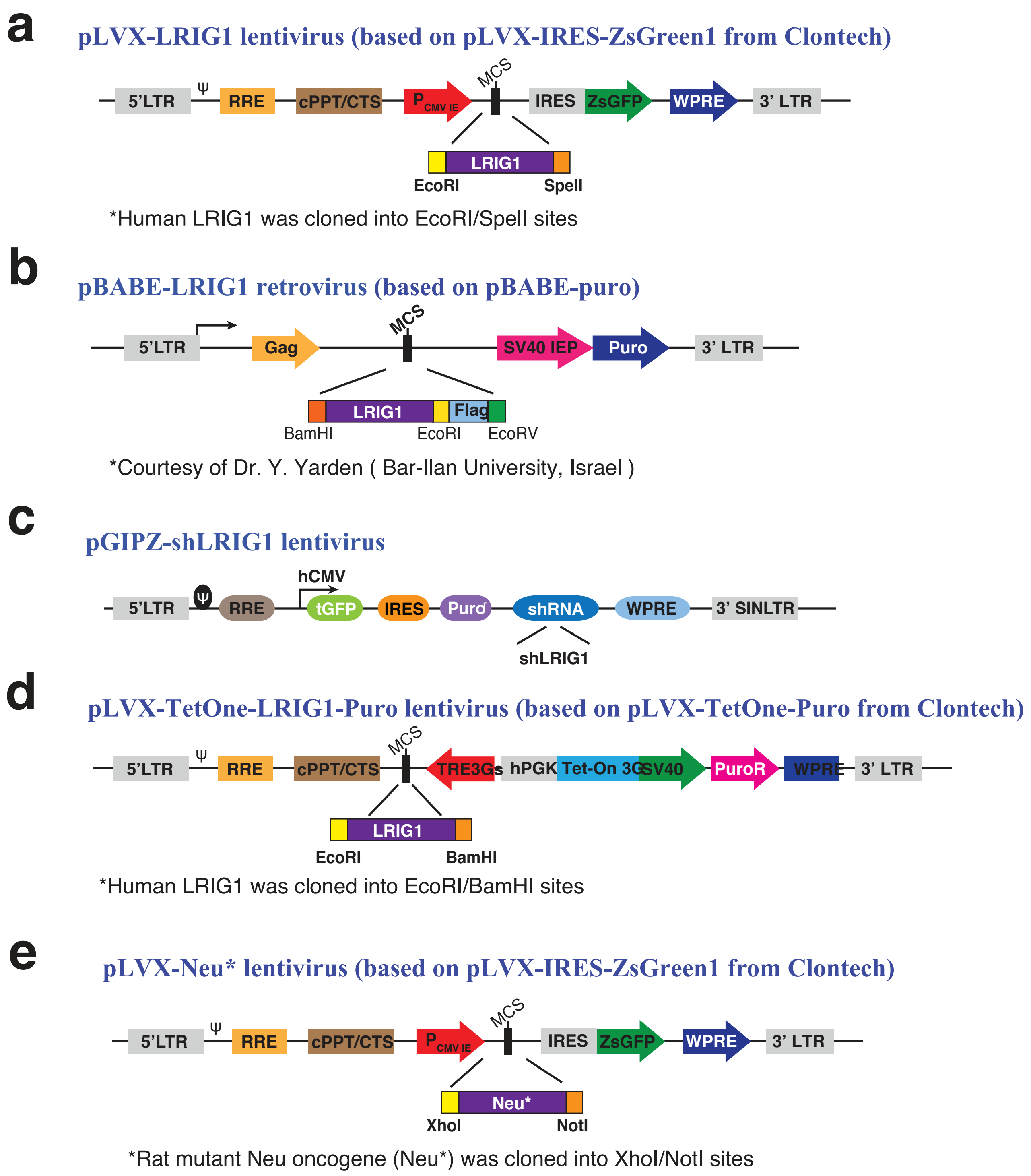

Supplementary Figure 5. Lentiviral and retroviral vectors used in this study.
(**a**) Lentiviral vector for overexpressing LRIG1. A 3,019 bp human LRIG1 cDNA was cloned into the EcoRI and SpeII sites of the Lenti-IRES-ZsGreen1 lentiviral expression vector (Clontech Laboratories).
(**b**) pBABE-LRIG1 retroviral vector.
(**c**) pGIPZ-shLRIG1 lentiviral vector (Open Biosystems).
(**d**) Inducible lentiviral vector for overexpressing LRIG1. The same 3,019-bp LRIG1 cDNA was cloned into the EcoRI and NheI sites of the pLVX-TetOne-Puro lentiviral vector (Clontech Laboratories).
(**e**) A mutated rat ErbB2 (Neu*) cDNA was cloned into the XhoI and NotI sites of the pLVX-IRES-zGreen1 lentiviral vector.

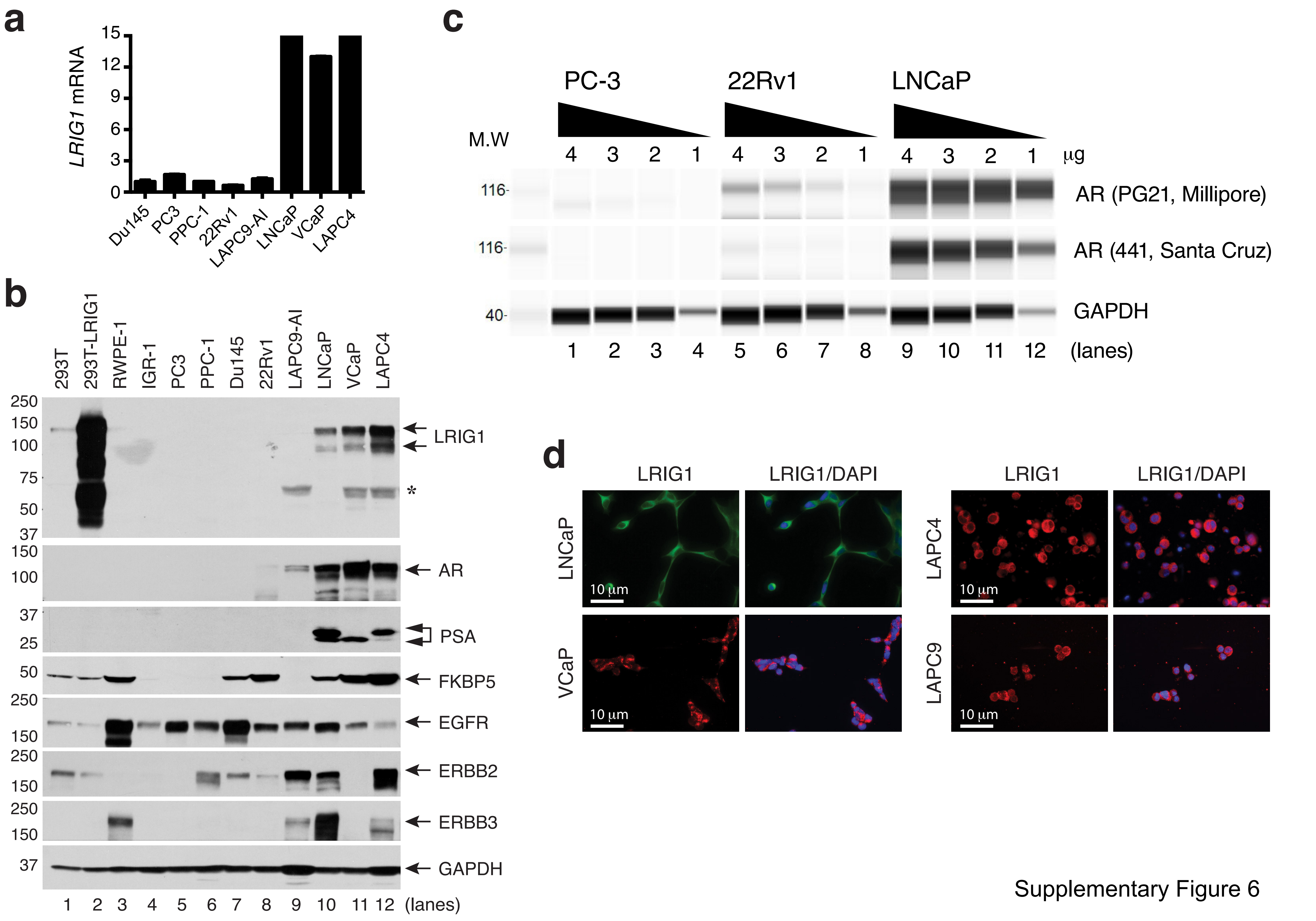

Supplementary Figure 6

Supplementary Figure 6. LRIG1 mRNA and protein expression in cultured PCa cells and xenografts

(**a**) qRT-PCR analysis of *LRIG1* mRNA levels in 8 PCa cell types indicated. LAPC4 cells were purified from androgen-dependent (AD) xenografts and LAPC9-AI (androgen-independent) were purified from AI xenograft tumors. The rest were cultured cells. The expression levels in each cell type were normalized to GAPDH and data was presented as relative to that in Du145 cells (which is 1). Bars represent the mean ± S.D from triplicate samples. Note that the LRIG1 mRNA levels were high in the 3 PCa cell types (i.e., LNCaP, VCaP, and LAPC4) that expressed high levels of AR protein (see below).

(**b**) Western blot (WB) analysis of LRIG1 protein using Sigma anti-LRIG1 mAb. RWPE-1 is an HPV18 immortalized non-tumorigenic prostate epithelial line (ATCC) and IGR-1 (i.e., IGR-CaP1) was initially derived from a primary prostate tumor (ref. 86). 293T cells infected with pBabe-LRIG1 (lane 2) was used as positive control. 60 µg/lane of protein/sample (except 293T-LRIG1; 10 µg) was used in WB of LRIG1 and other molecules indicated. Note that LRIG1 was detected, prominently, in the 3 PCa cells that expressed high levels of AR (mAb 441; Supplementary Table 1)and PSA proteins (lanes 10-12), as a major ~143 kDa band and a minor ~110 kDa band, the latter of which might represent the cleavage product of the 143 kDa band. Another ~60 kD band (*) was also observed, which might represent the cleaved ECD fragment (Supplementary Figure 1c). Two AI (castration-resistant) PCa cells, 22RV1 and LAPC9-AI, expressed much reduced AR and also negligible LRIG1 (lanes 8 and 9). Also note that 22Rv1 cells expressed low levels of AR (see below). Shown below are WB of 3 ERBB proteins (note that prostate and PCa cells do not express ERBB4).

(**c**) Detection of low levels of AR protein in 22Rv1 cells using ultra-sensitive Wes system (www.proteinsimple.com). Indicated amounts of whole cell lysates from the indicated cell types were loaded for AR detection using the two anti-AR antibodies (Supplementary Table 1). Note that the ~110 kD AR protein was readily detected in LNCaP cells, negative in PC3 cells, and expressed at low levels in 22Rv1 cells.

(**d**) LRIG1 immunofluorescence (IF) staining in cultured LNCaP and VCaP (left), and xenograft-derived LAPC4 (AD) and LAPC9 (AD) cells (right) using LRIG1 mAb (Sigma). Note that LAPC9-AD cells used here in IF, unlike LAPC9-AI cells (b, lane 9), express high levels of AR. LAPC4 and LAPC9 cells were purified from AD xenografts, cytospun onto glass slides and then used in IF staining. Typical cell surface staining of LRIG1 was observed in all 4 cells. IF staining in PC3 and Du145 cells, or with 2° Ab alone showed only background staining (not shown). Scale bars = 10 µm (original magnifications, 400X).

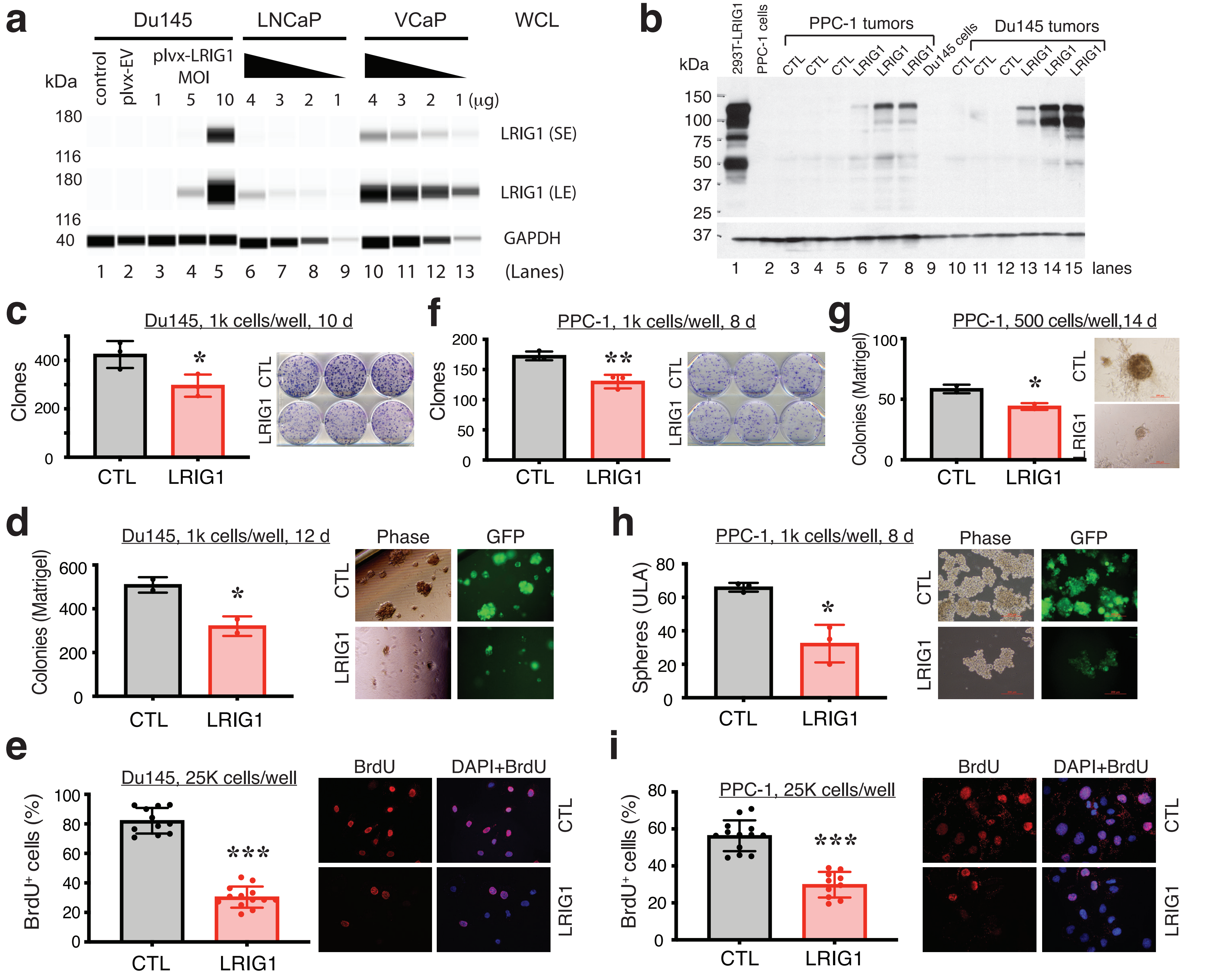

Supplementary Figure 7

Supplementary Figure 7. LRIG1 inhibits PCa cell clonal, clonogenic and sphere-forming properties and cell proliferation.

(a) Wes analysis of the levels of LRIG1 overexpression in Du145 cells. Du145 cells cultured in 12-well plates ($2x10_5$ cells/well) were infected with the pLVX-LRIG1 lentivirus at a MOI (Multiplicity of Infection) of 1, 5, and 10 (lanes 3-5) for 72 h (to determine the quantity of infectious virus, 10-fold dilutions of a virus stock were prepared, and 100 μl aliquots were gently put into 293T cell monolayers in 96-well plate (10,000 cells/well). After 72 h incubation, GFP_+ cells were counted under the KEYENCE microscope, and the MOI was calculated as GFP_+ cell number/the dilution). Du145 cells were also infected with the CTL empty pLVX vector at a MOI of 10 for 72 h (lane 2). Whole cell lysates (WCL) extracted from these infected Du145 cells as well as from the uninfected Du145 cells (lane 1) were loaded into the Wes capillaries (4 μg/lane) for LRIG1 detection, and compared with increasing amounts (1-4 μg/lane) of WCL from LNCaP (lanes 6-9) and VCaP (lanes 10-13) cells. Note that VCaP cells expressed higher levels of LRIG1 than LNCaP cells (consistent with earlier data; Supplementary Fig. 6b). Also note that Du145 cells infected with pLVX-LRIG1 at a MOI of 5 expressed LRIG1 at levels within the range of endogenous LRIG1 levels in both LNCaP and VCaP cells, but Du145 cells infected with pLVX-LRIG1 at a MOI of 10 expressed LRIG1 at levels above the range of endogenous LRIG1 levels in LNCaP and VCaP cells. In our pLVX-LRIG1 overexpression studies in $AR_{-/lo}$ PCa models, we generally used a MOI of 5-10. In this Wes experiment, the LRIG1 was detected using the Cell Signaling anti-LRIG1 antibody and both a short exposure (SE) and a long exposure (LE) were shown.

(b) Variable levels of LRIG1 in endpoint LRIG1-overexpressing PPC-1 and Du145 tumors. WCL prepared from 3 CTL and 3 LRIG1 Du145 (Fig. 2a) and PPC-1 (Fig. 2b) tumors, respectively, were used in regular WB analysis of LRIG1, together with cultured (uninfected) PPC-1 cells (lane 2), Du145 cells (lane 9), and 293T-LRIG1 cells (lane 1; positive control), using the Sigma mAb to LRIG1 ((60 μg/lane for all samples). GAPDH WB was used as loading control (below).

(**c-i**) LRIG1 inhibits PCa cell clonal, clonogenic and sphere-forming properties and cell proliferation. Du145 (c-e) and PPC-1 (f-i) cells were infected with pLVX-LRIG1 lentiviruses (LRIG1) or the control (CTL) empty lentivirus (MOI 10; 72 h), and plated for in vitro experiments indicated. In clonal assays, the infected Du145 (c) and PPC-1 (f) cells were plated in 6-well plates (1,000 cells/well). Clones were counted 14 days after plating.. In clonogenic assays in Matrigel, Du145 (d; 1,000 cells) and PPC-1 (g; 500 cells) were seeded in 12-well plates and colonies counted in 2 weeks. In sphere assays in ultra-low attachment (ULA) plates, PPC-1 cells (h, 1,000) infected as above were plated in 6-well ULA plates and cultured in serum-free medium. In BrdU incorporation assays, the infected Du145 (e) and PPC-1 (i), cells were plated and pulsed with BrdU for 4 h. Shown are representative images and quantification of BrdU-positive cells (at least 1,000 cells were counted per condition). All bar charts represent the mean ± SD (n=3). *P<0.05, **P<0.01, and ***P<0.001 when compared with the corresponding CTL groups (two-tailed unpaired Student's *t*-test).

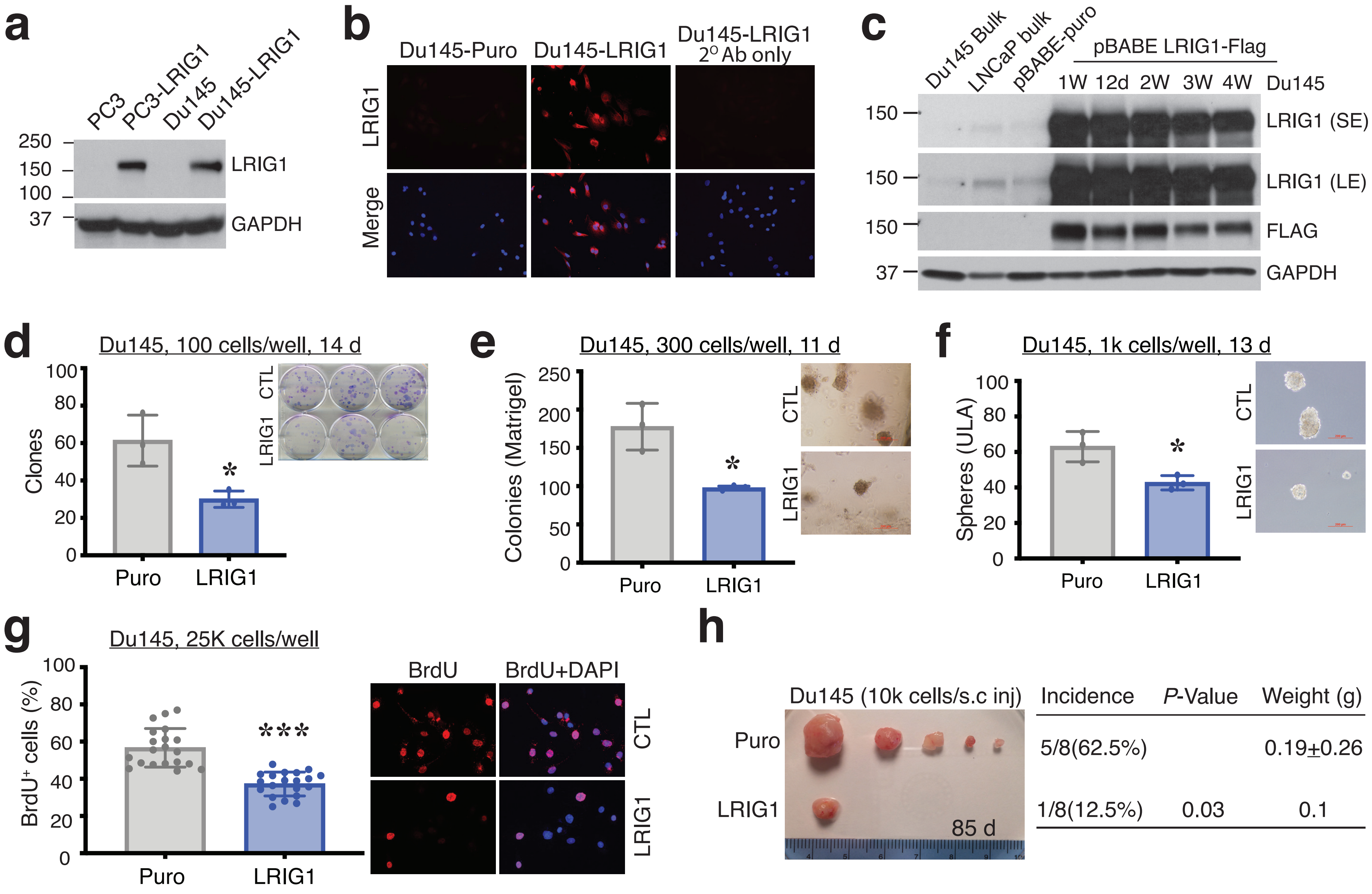

Supplementary Figure 8. Retrovival-mediated LRIG1 overexpression inhibits Du145 cell clonal, clonogenic, sphere-forming, and proliferative capacities *in vitro*, and tumor regeneration *in vivo*.

(**a-c**) Characterization of retrovival-mediated LRIG1 overexpression in PCa cells. Du145 and PC3 cells were infected with LRIG1 retroviral (pBABE-LRIG1-Flag) or control (pBABE-Puro) retroviral vectors for ~ 48-72 h at an MOI of 10. WB (a) and IF (b) were used to examine LRIG1 expression using the Sigma mAb. In (c), Du145 cells were infected with LRIG1 retroviral vectors, and selected by puromycin (1 μg/ml) for the time intervals indicated. Whole-cell lysates were extracted to determine LRIG1 levels by WB. An anti-Flag antibody was used to confirm LRIG1 expression. SE: short exposure; LE: long exposure.

(**d-h**) LRIG1 overexpression inhibits clonal (d), clonogenic (e), sphere-formation (f), and proliferative (g) capacities and tumor regeneration (h) of Du145 cells. Seeding densities and harvest time were indicated. In all graphs, bars represent the mean±SD (n=3). *$P<0.05$; ***$P<0.001$ when compared with the control (puro) groups (two-tailed unpaired Student's *t*-test).

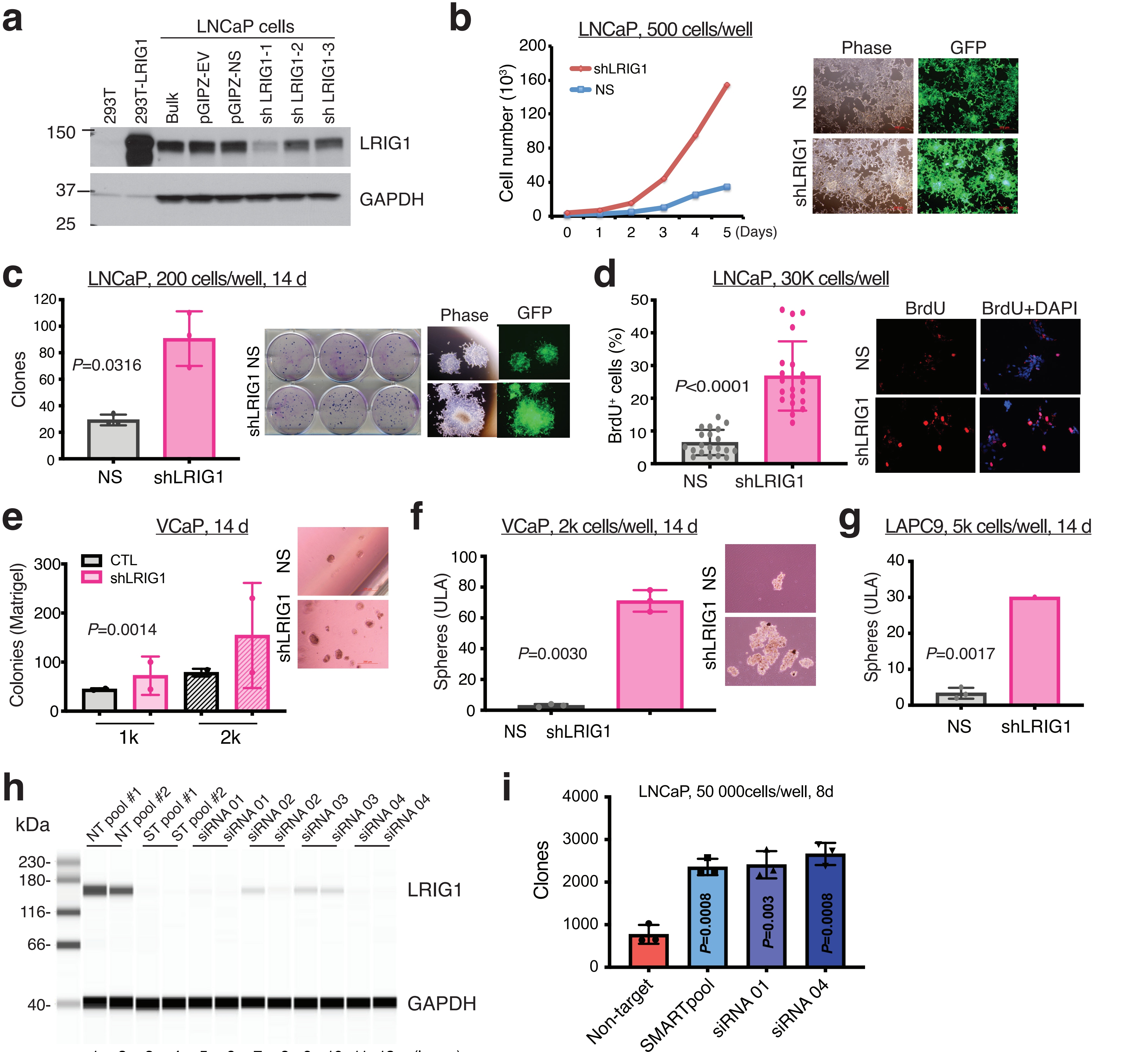

Supplementary Figure 9

Supplementary Figure 9. LRIG1 Knockdown promotes clonogenic, sphere-formation and proliferative capabilities in AR^+ PCa cells.

(**a-d**) Functional experiments with lentiviral-mediated LRIG1 knockdown (KD) in LNCaP cells. (**a**) WB analysis of KD efficiency by LRIG1 shRNA-encoding (pGIPZ-shLRIG1) lentiviral vectors. LNCaP cells were infected with 3 different pGIPZ-shLRIG1 vectors or with pGIPZ-EV (empty vector) or pGIPZ non-silencing (pGIPZ-NS; NS) control lentiviral vectors (MOI=10). shLRIG1 lentiviral vector clone-1 (i.e., shLRIG1-1) showed the strongest silencing effect and was used in all subsequent experiments. In cell growth (viability) assays (**b**), LNCaP cells infected with pGIPZ-NS or pGIZP-shLRIG1 (MOI=10) were plated (500k/well) in quadruplicate on day 0. On day 1, 2, 3, 4, and 5, cells were dissociated and counted by Trypan blue exclusion assays. Shown on the left is the growth curve based on the mean live cell numbers and on the right are GFP images on day 5. In clonal assays (**c**), infected LNCaP cells were plated in 6-well plates (500 cells/well) and clones were counted in 2 weeks. Representative GFP images were shown (right). For BrdU incorporation assays (**d**), shown on the left is the quantification of $BrdU^+$ cells from a total of 500-1000 cells counted. Representative images were presented on the right.

(**e-f**) LRIG1 KD experiments in VCaP cells. In clonogenic assays (**e**), VCaP cells infected with the CTL or pGIPZ-shLRIG1 (MOI=10) were mixed with Matrigel and plated in 12-well plates (1,000 or 2,000 cells/well) and colonies counted on day 14. In sphere assays (**f**), infected VCaP cells were seeded in 6-well ULA plates (2,000 cells/well) and spheres/spheroids were enumerated after 2 weeks.

(**g**) LRIG1 KD in LAPC9 cells promotes sphere formation. LAPC9 cells freshly purified from androgen-dependent xenograft tumors, infected with CTL or pGIPZ-shLRIG1 lentiviral vectors (MOI=10), and then seeded in 6-well ULA plates (5,000 cells/well). Spheres were enumerated after 2 weeks.

(**h-i**) siRNA-mediated LRIG1 KD promotes clonal growth in LNCaP cells. (**h**) Analysis of LRIG1 KD efficiency by Wes. Cultured LNCaP cells (50,000 cells/well in 6-well plate) were transfected, in duplicate, with 10 nM of LRIG1 SMARTpool (ST), non-targeting pool (NT), or 4 individual LRIG1-targeting siRNAs (see Methods). 72 h after transfection, cells were harvested and used in LRIG1 protein analysis by Wes (3 μg whole cell lysate/lane) using the Cell Signaling anti-LRIG1 antibody. Note that SMARTpool and siRNA 01 and 04 demonstrated the most prominent KD efficiency. (**i**) Clonal assays. LNCaP cells were plated in triplicate in 6-well culture plates (50,000 cells/well) and transfected with the indicated LRIG1-targeting siRNAs. The number of clones were determined on day 8 and *P*-values (in comparison with the non-targeting siRNA pool) were indicated in the bars (two-tailed Student's *t*-test).

In all other bar graphs, error bars represent the mean ± SD, and *P* values (two-tailed unpaired Student's *t*-test, compared to the corresponding controls) are indicated.

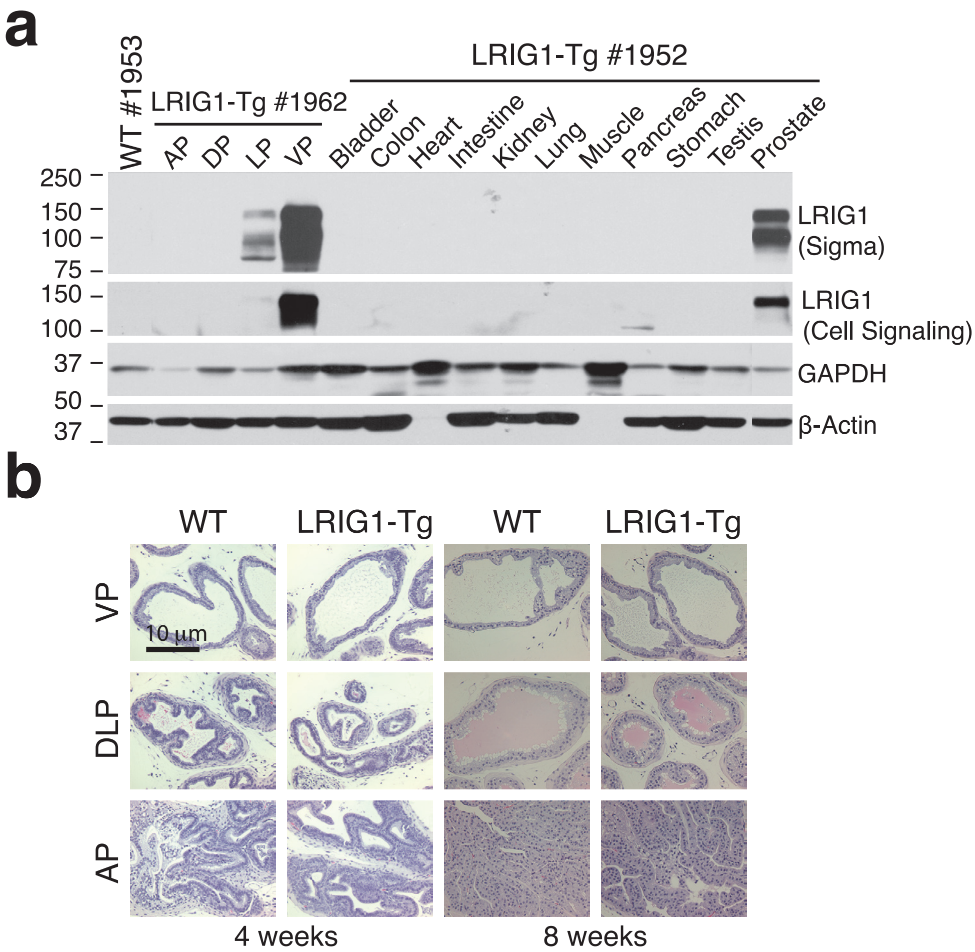

Supplementary Figure 10. Characterization of *ARR2PB*-LRIG1 transgenic (Tg) prostates

(**a**) WB analysis of human LRIG1 expression in different organs harvested from the LRIG1-Tg mouse (#1952) as well as the 4 prostate lobes (AP, DP, LP and VP represent anterior, dorsal, lateral, and ventral prostate, respectively) of the LRIG1-Tg animal #1962. The whole prostate lysate from a wild type (WT) mouse was also included (first lane). Both GAPDH and β-actin were used as loading control.

(**b**) Representative HE images of WT and LRIG1-Tg prostate lobes at 4 and 8 weeks. No significant differences in overall prostate morphologies and glandular structures were observed between the 2 different genotypes. Bar = 10 µm (for all images).

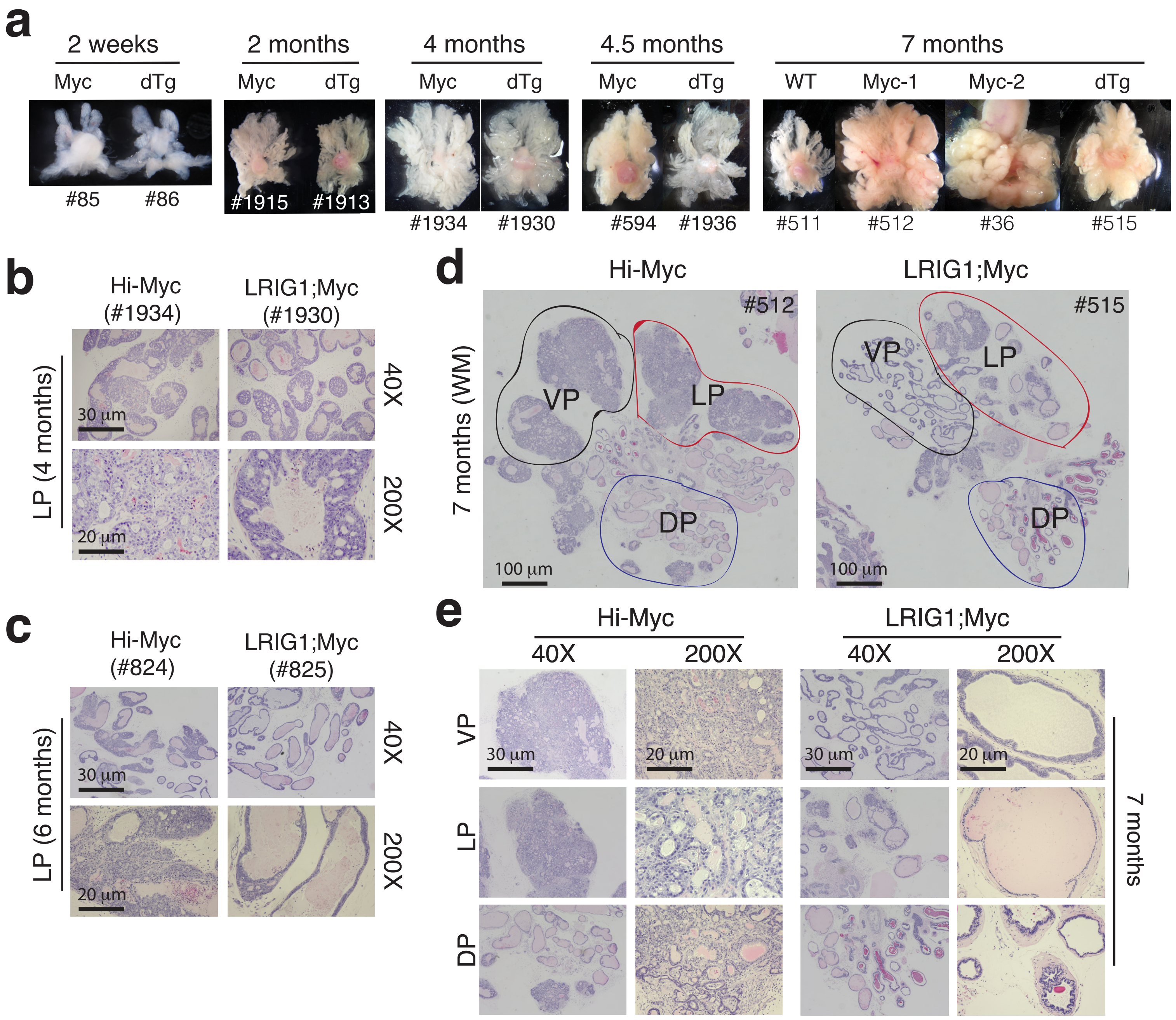

Supplementary Figure 11. LRIG1 inhibits Myc-driven PCa.

(**a**) Representative images of Hi-Myc and LRIG1;Myc (dTg) prostates at the indicated ages. Animal tag numbers are shown below.

(**b-c**) Representative HE images of Hi-Myc and LRIG1;Myc mouse prostates at 4 and 6 months, respectively.

(**d-e**) Representative HE images of prostate lobes in 7 month-old Hi-Myc and LRIG1;Myc mice. Shown in d are whole mount (WM) images with the prostate lobes indicated (VP, LP, and DP represent ventral, lateral, and dorsal prostate, respectively). Images in e are enlarged from d.

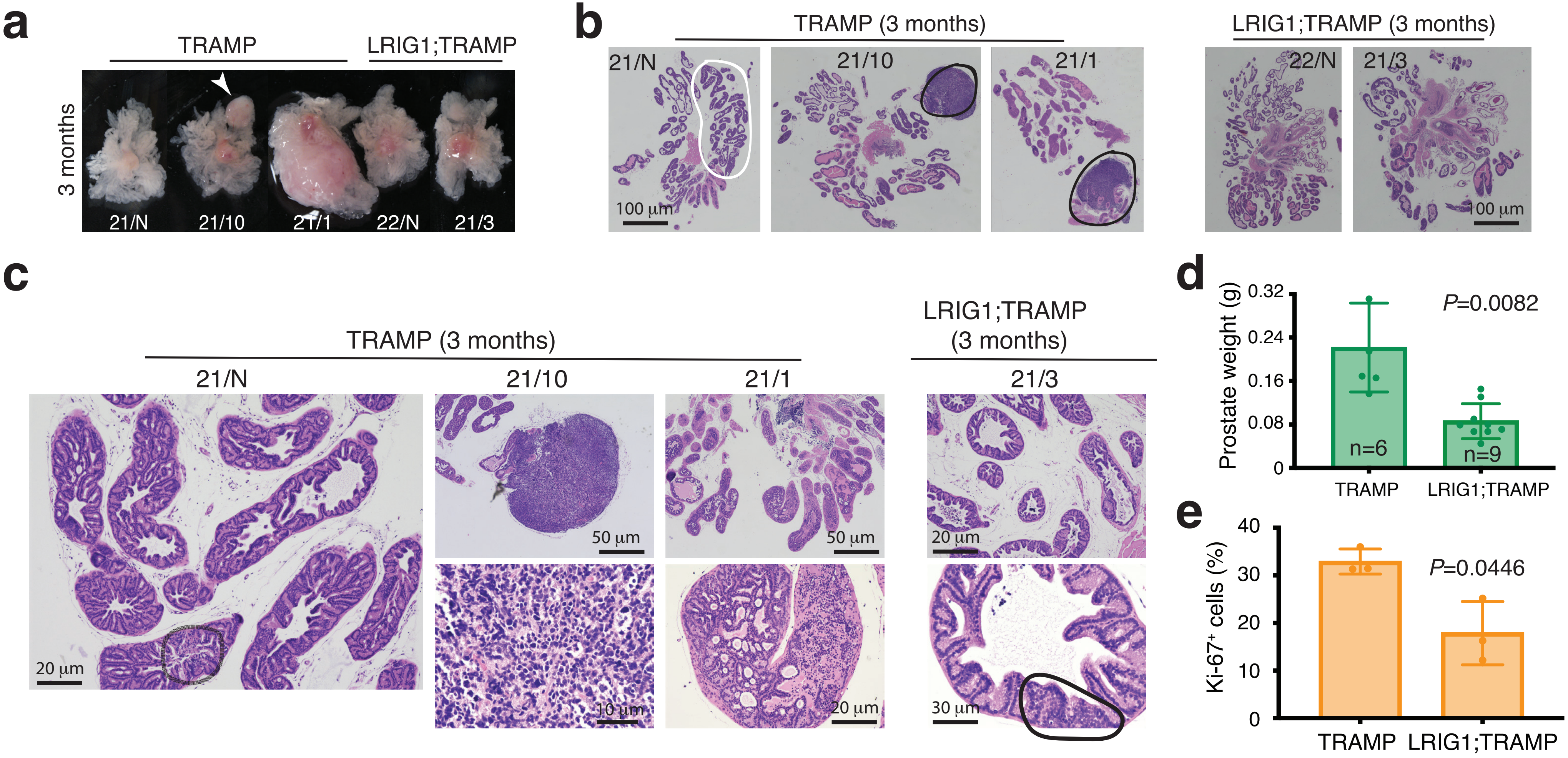

Supplementary Figure 12. LRIG1 inhibits TRAMP tumor development.

(**a-c**) Transgenic LRIG1 expression partially inhibits TRAMP tumor growth at 3 months. (a) Images of microdissected prostates in 3 TRAMP vs. 2 LRIG1;TRAMP mice. Animal tag numbers are indicated below. The white arrowhead indicates a tumor nodule. (b) Representative whole-mount HE images of the 3-month old TRAMP vs. LRIG1;TRAMP prostates presented in a. (c) Highly variable and pleiomorphic features of TRAMP prostates at 3 months. Shown are representative HE images of the 3 TRAMP prostates (21/N, 21/10, and 21/1) and 1 LRIG1;TRAMP prostate (21/3). Note that the two TRAMP prostates developed pleiomorphic and undifferentiated tumors (21/10 and 21/1) and the other showed focal low-grade PIN (prostate intraepithelial neoplasia, circled) whereas the LRIG1;TRAMP prostate in animal 21/3 only showed focal atypia (right, circled).

(**d**) Average prostate weights in 3 month-old TRAMP vs. LRIG1;TRAMP mice (n indicated). All 4 lobes from each animal were dissected out and weighed. The *P*-value was determined by two-tailed unpaired Student's *t*-test.

(**e**) Quantification of Ki-67^{+} cells in 3-month-old TRAMP and LRIG1;TRAMP prostates. Presented is the bar graph (mean +/- S.D) showing reduced Ki-67^{+} cells in LRIG1;TRAMP vs. TRAMP prostates (n=3 for each genotype; counting of 800-1,200 cells for each prostate).

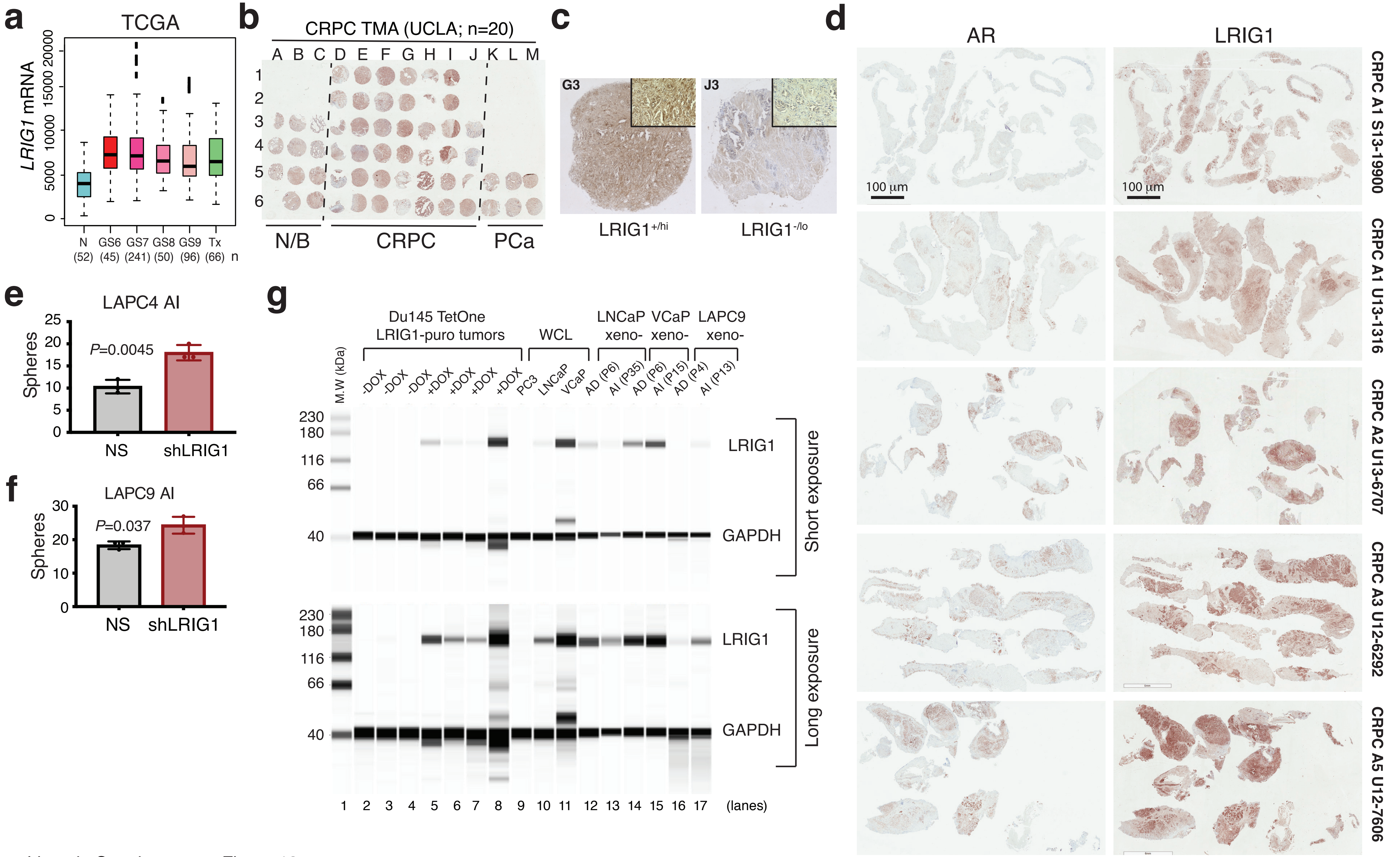

Li et al., Supplementary Figure 13

Supplementary Figure 13. Expression and functions of LRIG1 in CRPC and inducible expression of LRIG1

(a) LRIG1 mRNA changes in TCGA PCa samples. Shown are normalized mean *LRIG1* mRNA levels in normal prostate tissues (N), untreated PCa of increasing tumor grade (i.e., Gleason Scores (GS) 6-9), and treated (Tx) samples. The number of each type of samples is indicated in parentheses below. The differences between each type of PCa samples versus normal tissues are all statistically significant (P<0.000001; Student's t-test) whereas the differences between PCa of various Gleason grade are not statistically significant. The centerlines in boxplots represent the medians, box edges 25_{th} and 75_{th} percentiles, and whiskers the maximum and minimum values.

(**b-c**) Heterogeneous expression of LRIG1 in CRPC specimens. (b) IHC staining (Sigma mAb) reveals heterogeneous LRIG1 protein expression in the UCLA CRPC-TMA (see Methods), which contained 20 CRPC (middle, demarcated by dashed lines), 3 untreated PCa, and 6 normal/benign (N/B) prostate samples (duplicate spots for all samples). Shown in (c) are examples of CRPC expressing high vs. low levels of LRIG1 (insets: 400×).

(**d**) Discordant LRIG1 and AR expression patterns in CRPC. Consecutive FFPE sections in 6 cases of whole-mount CRPC specimens including the case shown in Fig. 5a (case# indicated) were stained for AR and LRIG1 (Sigma mAb). Note discordant expression patterns of AR and LRIG1 in that although some AR-positive PCa cells expressed LRIG1, the majority of AR-negative PCa cells also stained positive for LRIG1 (also see Fig. 5a).

(**e-f**) Knocking down endogenous LRIG1 in LAPC4 (e) and LAPC9 (f) AI tumor cells increased sphere formation. For sphere formation assays, LAPC9 and LAPC4 AI cells acutely purified from the respective AI tumors (maintained in castrated mice) were infected with the pGIPZ-NS (non-silencing control) or pGIPZ-shLRIG1 lentiviral vectors (MOI 10; 12 h) and seeded in 6-well ultra-low attachment (ULA) plates (1,000 cells/well) and spheres were enumerated 2 weeks after plating. Bar charts represent the mean ± S.D (n=3) and P values determined by two-tailed unpaired Student's t-test.

(**g**) Assessment, by Wes system, of LRIG1 levels in the indicated cultured PCa cells, PCa xenografts, and endpoint Du145 tumors infected with the DOX-inducible LRIG1 lentiviral vector. For all samples, whole cell lysates (WCL) were used in Wes analysis (3 μg/lane) of LRIG1 using the Cell Signaling antibody, and both short- and long-exposure images were presented. For cultured PC3, LNCaP and VCaP cells (lane 9-11), VCaP cells expressed significantly more LRIG1 than LNCaP cells and PC3 cells were negative for LRIG1. For the 3 xenograft AD/AI pairs of tumors analyzed (lanes 12-17), late passage VCaP and LAPC9 AI tumors showed higher levels of LRIG1 than corresponding earlier passaged AD tumors whereas a late-passage LNCaP AI tumor (P35) displayed lower levels of LRIG1 than an earlier passaged LNCaP AD tumor (P6). Note that, although the 3 control endpoint Du145 tumors in the absence of DOX showed no LRIG1 expression (lanes 2-4), the 4 DOX-treated Du145 tumors derived from the cells infected with the TetOne LRIG1-puro lentivectors (lanes 5-8) expressed LRIG1 at levels similarly to those in cultured LNCaP or VCaP cells or in the AD/AI xenograft tumors.

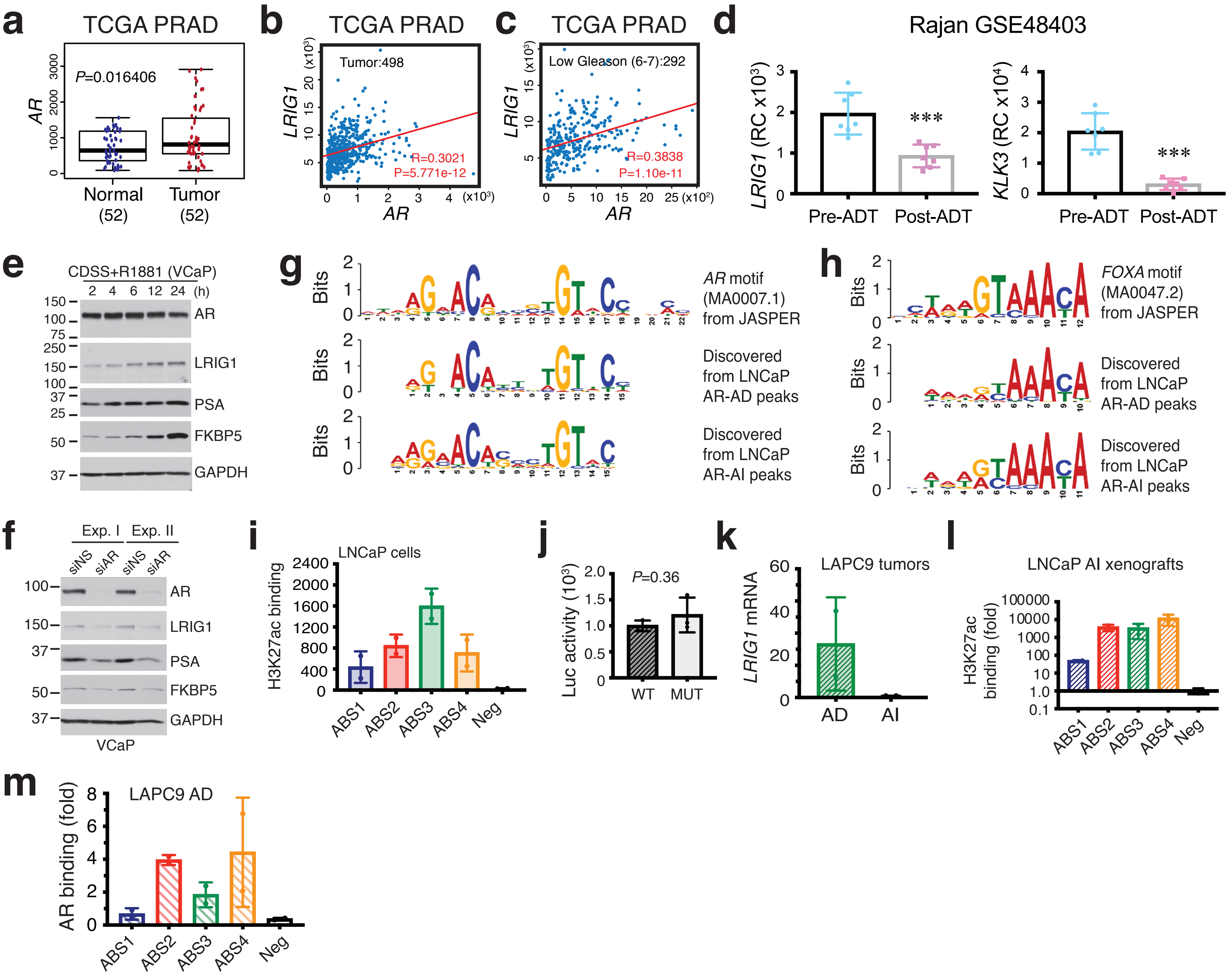

Li, et al., Supplementary Fig. 14

Supplementary Figure 14. AR directly regulates LRIG1 in PCa cells.

(**a**) Increased *AR* mRNA levels in the 52 prostate tumors compared to matched normal tissues in the TCGA PRAD dataset. The center lines in the box plots represent median values, box edges are 75th and 25th percentiles, and whiskers represent the maximum and minimum values, respectively.

(**b**-**c**) Positive correlations between *AR* and *LRIG1* mRNA levels in the entire TCGA PRAD cohort of prostate tumors (n=498 samples; b) or in low-grade (GS6-7) tumors (n=292 samples; c). Note the slightly better positive correlation between *AR* and *LRIG1* in low-grade tumors (compare the R value in b).

(**d**) In the Rajan dataset (GSE48403), the mRNA levels of both *LRIG1* and *KLK3* (*PSA*) were decreased in PCa patients post ADT treatment. ****P*<0.001 (two-tailed unpaired Student's *t*-test).

(**e**) R1881 (0.1 nM) induces upregulation of LRIG1 and other AR targets (PSA and FKBP5) in VCaP cells. Shown is a time course study.

(**f**) Knocking down endogenous AR reduces AR targets PSA and FKBP5 as well as LRIG1. VCaP cells were transfected with AR siRNAs (siAR, 20 nM; 72 h) or control siRNAs (siNS) and then harvested for Western blotting analysis of the molecules indicated. Results of two independent experiments (Exp) were shown.

(**g**-**h**) The AR (g) and FOXA (h) motifs identified in AR-binding ChIP-Seq peaks on LRIG1 genomic region (see Fig. 7c).

(**i**) ChIP-qPCR showing enrichment of acetylated lysine 27 of histone 3 (H3K27ac) in all 4 ABS in the *LRIG1* genomic region. Bar charts represent the relative H3K27ac binding compared to IgG control (mean ± S.D; n=3).

(**j**) Luciferase activities in LNCaP cells transfected with ABS1-WT or ABS-MUT constructs, both of which were normalized to LNCaP cells transfected with the control empty pREPORT vector. The relative luciferase activities in ABS1-WT cells were taken as the baseline (1) over which the luciferase activities in ABS1-MUT transfected LNCaP cells were compared (mean ± SEM from 3 independent experiments with quadruplicate measurements in each sample in each experiment). Note that mutation of the ARE eliminated increased luciferase activities observed in ABS1-WT transfected cells (see also Fig. 7j).

(**k**) qRT-PCR analysis demonstrating reduced *LRIG1* mRNA levels in LAPC9 AI tumors compared to AD tumors. Bars represent the mean ± S.D (n = 3-4 tumors each; *P*<0.001, two-tailed unpaired Student's *t*-test).

(**l**) ChIP-qPCR analysis demonstrating low H3K27ac binding to the ABS1 but significant H3K27ac binding to ABS2-4 in LNCaP AI xenografts. Bar charts represent the relative H3K27ac binding compared to IgG control (mean ± S.D; n=3).

(**m**) ChIP-qPCR analysis demonstrating AR binding to ABS2 and ABS4 in AD LAPC9 xenograft cells. Bar charts represent the relative AR binding compared to IgG control (mean ± S.D; n=3).

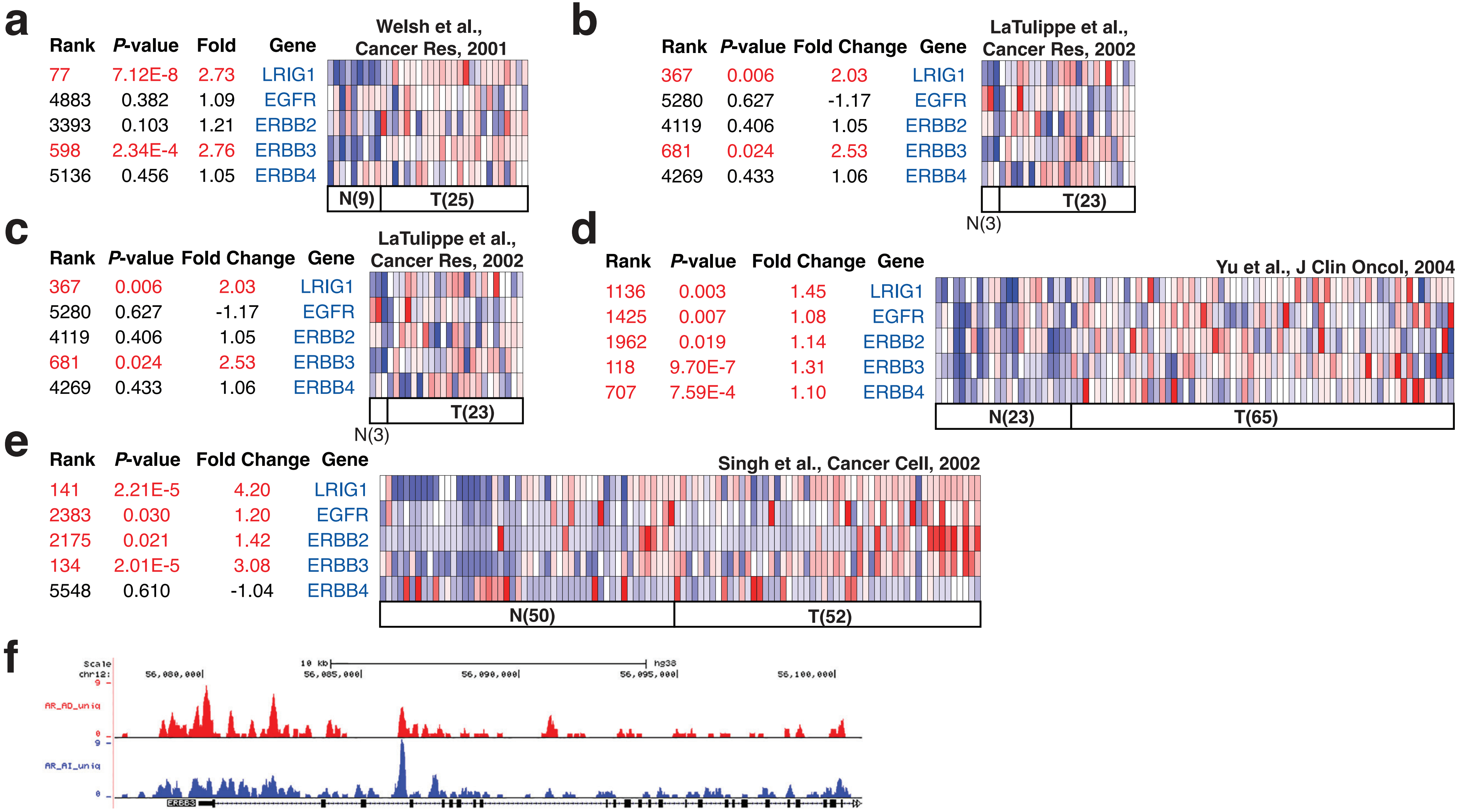

Supplementary Figure 15. ERBB expression and potential relationship with LRIG1 and AR in PCa

(**a-e**) Co-upregulation of *LRIG1* and *ERBB3* mRNA levels in PCa (T) compared to normal (N) tissues in 5 representative Oncomine datasets. Note that in all 5 datasets, the median gene rank of ERBB3 is the highest among the 4 ERBB members. In the Yu (d) and Singh (e) datasets, *EGFR* and *ERBB2* mRNA levels were also slightly upregulated in T compared to N (see Fig. 8c-d).

(**f**) UCSC genome browser tracks showing zoom-in images of AR binding to the *ERBB3* genomic region (see Fig. 8i).

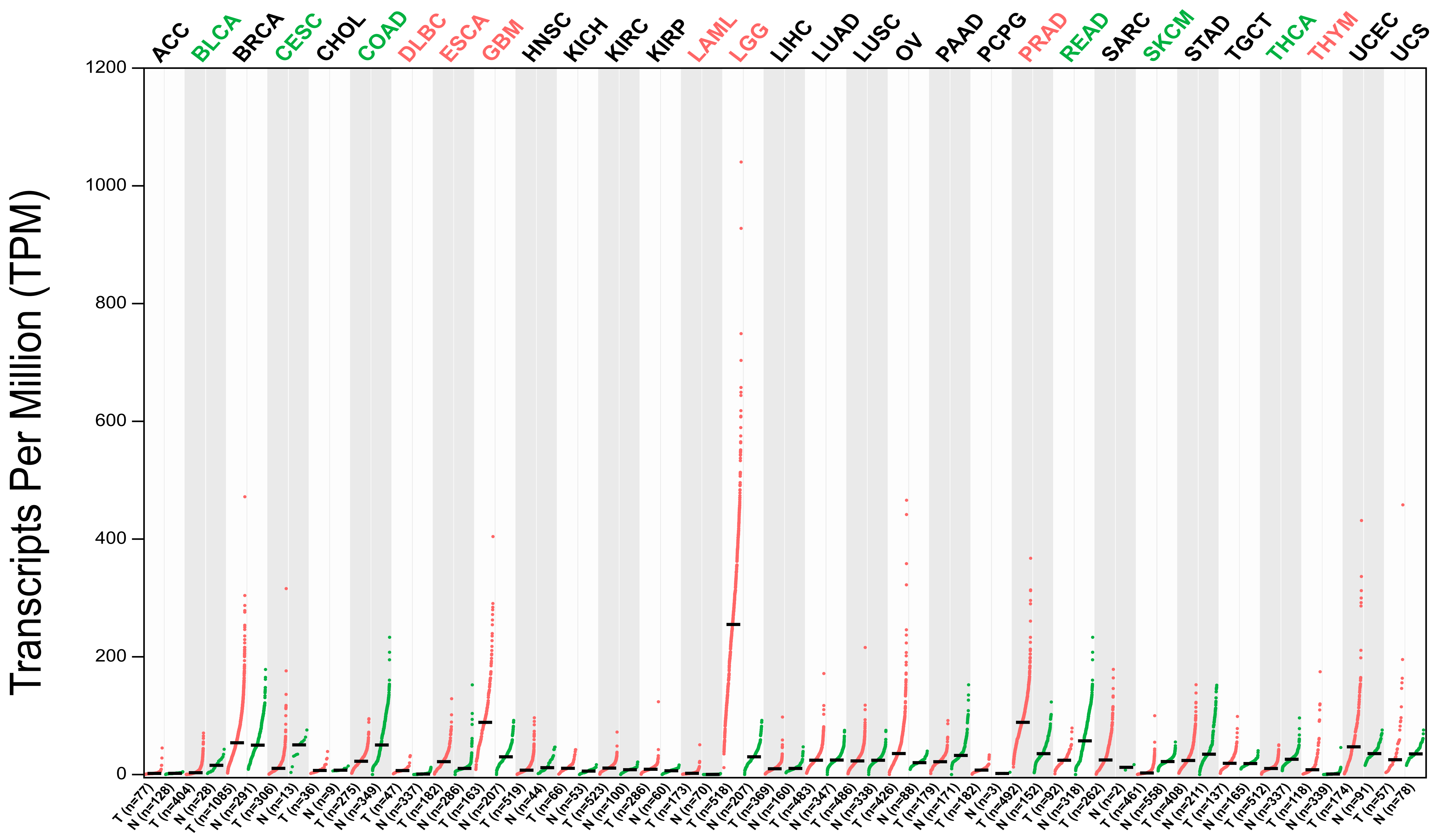

Li et al., Supplementary Figure 16

Supplementary Figure 16. LRIG1 mRNA expression in 31 human cancers compared to matched normal tissue.

The *LRIG1* mRNA levels (TPM), extracted from the TCGA tumors and normal tissue samples, are upregulated in 7 (red) and downregulated in 6 (green) human cancers (indicated above; *P*<0.05, Student's *t*-test). The abbreviations for each cancer are shown below.

ACC	Adrenocortical carcinoma
BLCA	Bladder Urothelial Carcinoma
BRCA	Breast invasive carcinoma
CESC	Cervical squamous cell carcinoma and endocervical adenocarcinoma
CHOL	Cholangiocarcinoma
COAD	Colon adenocarcinoma
DLBC	Lymphoid Neoplasm Diffuse Large B-cell Lymphoma
ESCA	Esophageal carcinoma
GBM	Glioblastoma multiforme
HNSC	Head and Neck squamous cell carcinoma
KICH	Kidney Chromophobe
KIRC	Kidney renal clear cell carcinoma
KIRP	Kidney renal papillary cell carcinoma
LAML	Acute Myeloid Leukemia
LGG	Brain Lower Grade Glioma
LIHC	Liver hepatocellular carcinoma
LUAD	Lung adenocarcinoma
LUSC	Lung squamous cell carcinoma
OV	Ovarian serous cystadenocarcinoma
PAAD	Pancreatic adenocarcinoma
PCPG	Pheochromocytoma and Paraganglioma
PRAD	Prostate adenocarcinoma
READ	Rectum adenocarcinoma
SARC	Sarcoma
SKCM	Skin Cutaneous Melanoma
STAD	Stomach adenocarcinoma
TGCT	Testicular Germ Cell Tumors
THCA	Thyroid carcinoma
THYM	Thymoma
UCEC	Uterine Corpus Endometrial Carcinoma
UCS	Uterine Carcinosarcoma

Supplementary Table 1. Primary antibodies used in the current study

Antibody	Source	Company	Catalog #	Remarks
β-Actin	Mouse mAb	Santa Cruz	SC-47778	(C-4)
AMACR	Rabbit mAb	Dako	M3616	P504s
AKT	Rabbit mAb	Cell signaling	4691	C67E7
AR	Mouse mAb	Santa Cruz	SC-7305	441
AR	Rabbit pAb	Santa Cruz	SC-816	N-20
AR	Rabbit mAb	Millipore Sigma	06-680	PG-21
BCL-2	Mouse mAb	BD	610538	
BrdU	Mouse mAb	Sigma	B2531	
c-MYC	Rabbit mAb	Abcam	Ab32072	Y69
c-MYC	Mouse mAb	Sigma	M4439	9E10
Caspase-3	Rabbit pAb	R&D	AF835	
Cleaved LAMIN A/C	Rabbit pAb	Cell Signaling	2035	
EGFR XP	Rabbit mAb	Cell signaling	4267	D38B1
EGFR	Rabbit pAb	Sigma	HPA018530	C225
ERBB2 XP	Rabbit mAb	Cell signaling	4290	
ERBB2	Rabbit pAb	Santa Cruz	SC-284	C-18
ERBB3	Mouse mAb	Millpore	05-390	
ERBB3	Rabbit pAb	Santa Cruz	SC-285	C-17
Flag	Mouse mAb	Sigma	F1804	
FKBP5	Rabbit pAb	Cell Signaling	8245	FL-335
GAPDH	Rabbit pAb	Santa Cruz	SC-25778	
H3 (K27acetyl)	Rabbit mAb	Abcam	Ab4729	
Ki67	Rabbit pAb	Leica Biosystems	NCL-Ki67p	Clone MM1
Ki67	Rabbit pAb	Novus Biological	NB110-89717	
LAMIN A/C	Rabbit pAb	Santa Cruz	SC-20681	H-110
LRIG1	Mouse mAb	Sigma	SAB4200445	
LRIG1	Rabbit pAb	Abcam	Ab30707	
LRIG1	Rabbit pAb	Cell Signaling	12752	
LRIG1	Sheep pAb	R&D	AF7498	aa 35-779
LRIG1	Goat pAB	R&D	AF3688	aa 37-794
LRIG1	Goat pAb	Santa Cruz	SC-50075	G-20 aa 620-670
LRIG1	Goat pAb	Santa Cruz	SC-50076	P16
p44/42 MAPK(ERK1/2)	Rabbit mAb	Cell signaling	4695	(137F5)
PCNA	Mouse mAb	Abcam	ab29	PC10
pERK1/2	Rabbit pAb	Cell signaling	9101	(Thr202/Tyr204)
pAKT	Rabbit mAb	Cell signaling	4060	(SER473)D9E
pEGFR	Rabbit mAb	Abcam	Ab40815	(Y1092)[EP774Y]
pERBB2	Rabbit mAb	Cell signaling	2243	(Tyr1221/1222) 6B12
pERBB2	Rabbit Ab	Cell signaling	2247	(Tyr1248)
pERBB3	Rabbit mAb	Cell signaling	4791	(Tyr1289) 21D3
pERBB3	Rabbit mAb	Cell signaling	2842	(Tyr1289) D1B5
PSA	Goat pAb	Santa Cruz	SC-7638	C-19

*Abbreviations: mAb, monoclonal antibody; pAb, polyclonal antibody.

Supplementarys Table 2. Primary tumor (HPCa) samples and TMAs used in the current study*

Patient	Age	Gleason	Experiments[#]
HPCa38	67	9 (4+5)	HE, IHC
HPCa59	55	9 (4+5)	HE, IHC
HPCa67	65	6 (3+3)	HE, IHC, IF
HPCa69	74	8 (4+4)	HE, IHC, IF
HPCa88	72	9 (5+4)	HE, IHC, IF
HPCa90	66	8 (4+4)	HE, IHC
HPCa93	58	7 (3+4)	HE, IHC
HPCa97	74	7 (3+4)	IF
HPCa105	63	9 (4+5)	IF
HPCa106	50	7 (3+4)	IF
HPCa118	58	7 (3+4)	IF
HPCa139	52	7 (3+4)	HE, IHC, IF
HPCa146	56	7 (3+4)	HE, IHC, IF
HPCa161	74	9 (4+5)	IF, WB
HPCa169	66	7 (3+4)	IF, WB
**TMA code**	**Patients No.**	**Gleason**	**Experiments**
TMA 75	75	N+T	IHC
TMA 115	115	N+T	IHC
TMA 124	124	N+T	IHC

*HPCa samples were obtained from radical prostatectomy by DaVinci procedure. From all samples, pieces of tumors and corresponding benign tissues were used in the experiments indicated. The 3 TMAs (tissue microarrays) contained matched normal (N) and tumor (T) samples from the indicated number of patients.

[#]Abbreviations: HE, Hematoxylin and eosin staining; IF, Immunofluoresence staining; WB, Western blot; IHC, immunohistochemistry

Supplementary Table 3: Primers, probes, and shRNAs used in this study

**qPCR genes**	**Forward (5' – 3')**	**Reverse (3' – 5')**
hGAPDH	ACTTTGGTATCGTGGAAGGACT	GCCTTGGCAGCGCCAGTAG
mGapdh	TGACCTCAACTACATGGTCTACA	CAAGGCCGAGAATGGGAAG
hLRIG1	TCTCCTTCTCTGGCTGCTTTTG	GTTGTAACTCAGGTTTAGGCTCCG
mLrig1	TTGAGGACTTGACGAATCTGC	CTCTCTTTTTGCAGCACAACAAG
EGFR	CCCACTCATGCTCTACAACCC	TCGCACTTCTTACACTTGCGG
ERBB2	TGTGACTGCCTGTCCCTACAA	CCAGACCATAGCACACTCGG
ERBB3	GACACAATTGACTGGAGGGACA	GACCATTACACTGAGGAGCACA
ERBB4	GGCCATTCCACTTTACCACAAC	ACTGGGAGTTAATGGTTCCACC
Human specific LRIG1	CAAGAGATGCAAGCCACTTTCC	GAGTAACAGTCCACTTCGGTGT
Mouse specific Lrig1	TGGAAGACGCCATATCTACTGA	TCTTTCCTGTAGCGACTGCTTT
**ChIP-qPCR**	**Forward (5' – 3')**	**Reverse (3' – 5')**
ABS1	TCGACTAGGCGAAAACAGATGA	TCTCCATTGTATCCTCAGCACG
ABS2	ACCTGCATCCTGGTTCATAAGG	CACCTGTACCTGAGTTCCTTCC
ABS3	GGACTAGCACAAAAGAGCTTGC	CTGTGTTTTTCCAAAGGGCGTT
ABS4	GGGGAATTTTGGCAGATTCTCA	TGACCCTAAAGGAAGTGAACGG
Negative	GCTCCTTGCCCTTGGATAGG	TCAATGTGCAAACTCCGGTCT
**Genotyping**	**Forward (5' – 3')**	**Reverse (5' – 3')**
LRIG1-Tg	GCTGACACAACTGGACCTCAATC	GAATGCTCTCTTAGCCACAGACTTG
Hi-Myc	CAATGTCTGTGTACAACTGCCAACT GGGATGC	TTACGCACAAGAGTTCCGTAGCTGTTC
TRAMP	GCGCTGCTGACTTTCTAAACATAAG	GAGCTCACGTTAAGTTTTGATGTGT
Lrig1 KO	For: ACTCACTGCGGGACTCCACAGA	WT Rev: GCAAGAGCAGCCAGAGCAGAAG<br>Mut Rev: TCCCCAGAAATGCCAGATTACG
**shRNA**	**Clone ID**	**Mature Antisense**
GIPZ-shLRIG1 -1	V2LHS_229246	TTAAACAGCAACCTGATAC
GIPZ-shLRIG1 -2	V3LHS_404471	TGTGATGGATTAACAACCC
GIPZ-shLRIG1 -3	V3LHS_332387	ACTCTTCACTCTTCTTCCT
**PCR and Sequencing**	**Forward**	**Reverse**
PCR for LRIG1 Site1	ACCTCGAGCAGGAAACTCACCTCGA TGACA	GCAGATCTGTGTCCTGGGCCATGAGATA AT
PCR for hLRIG1	TAGAATTCATGGCGCGGCCGGTCC	GCGCTAGCCTAGCTTTTTGGTGCC
Sequencing for hLRIG1	CTAACTCTTCGCCTGAGCAAAAAC<br>TGAACCTTGGAGGGAATGCG<br>AAACGCCCCACGACATAACC<br>CCAACACAGATGAAACCGTCG	
PCR for ratNeu*	GCGAATTCATGATCATCATGGAGC	TAGCGGCCGCTCATACAGGTACATCC
Sequencing for ratNeu*	GGACATCCAGGAAGTTCAGGGTTAC<br>CCAGGCAGTCAGAATGCTTGG<br>CGCAAGAATAACCAACTGGCTCC<br>CAACTACCTGTCTACGGAAGTGGG<br>GCTCTGATTCACCGCAACGC<br>TGCCCCAGTGGTGTGAAACC<br>GGAGAATGTGAAAATCCCCGTG<br>GGCTGCTGGACATTGATGAGAC<br>CTACCGTTCACTGCTGGAAGATG<br>GCTCCGATGTGTTTGATGGTGAC	

**Taqman Assays(mouse)**	**Supplier**	**Assay ID**
LRIG1	Thermofisher Scientific	Mm00456116_m1
Myc	Thermofisher Scientific	Mm00487803_m1
GusB	Thermofisher Scientific	Mm01197698_m1
