# Supplementary material for: LRIG1 is a pleiotropic androgen receptor-regulated feedback tumor suppressor in prostate cancer
Source: Nat Commun. 2019 Dec 2;10:5494. doi: 10.1038/s41467-019-13532-4 (PMC6889295; doi:10.1038/s41467-019-13532-4)
Supplement: Supplementary file 2 — Reporting Summary [file 41467_2019_13532_MOESM2_ESM.pdf]

nature research

Corresponding author(s): Dean Tang

Last updated by author(s): Jul 24, 2019

Reporting Summary

Nature Research wishes to improve the reproducibility of the work that we publish. This form provides structure for consistency and transparency in reporting. For further information on Nature Research policies, see Authors & Referees and the Editorial Policy Checklist.

Statistics

For all statistical analyses, confirm that the following items are present in the figure legend, table legend, main text, or Methods section.

n/a	Confirmed	
☐	☒	The exact sample size (*n*) for each experimental group/condition, given as a discrete number and unit of measurement
☐	☒	A statement on whether measurements were taken from distinct samples or whether the same sample was measured repeatedly
☐	☒	The statistical test(s) used AND whether they are one- or two-sided<br>*Only common tests should be described solely by name; describe more complex techniques in the Methods section.*
☐	☒	A description of all covariates tested
☐	☒	A description of any assumptions or corrections, such as tests of normality and adjustment for multiple comparisons
☐	☒	A full description of the statistical parameters including central tendency (e.g. means) or other basic estimates (e.g. regression coefficient) AND variation (e.g. standard deviation) or associated estimates of uncertainty (e.g. confidence intervals)
☐	☒	For null hypothesis testing, the test statistic (e.g. *F*, *t*, *r*) with confidence intervals, effect sizes, degrees of freedom and *P* value noted<br>*Give P values as exact values whenever suitable.*
☒	☐	For Bayesian analysis, information on the choice of priors and Markov chain Monte Carlo settings
☒	☐	For hierarchical and complex designs, identification of the appropriate level for tests and full reporting of outcomes
☒	☐	Estimates of effect sizes (e.g. Cohen's *d*, Pearson's *r*), indicating how they were calculated

Our web collection on statistics for biologists contains articles on many of the points above.

Software and code

Policy information about availability of computer code

Data collection	At the endpoint, tumors were harvested and various parameters were recorded, including tumor images, tumor weight, incidence, latency, and tumor-initiating frequency (TIF) (http://bioinf.wehi.edu.au/software/elda/).
Data analysis	HE or IHC stained glass slides containing sections of patient tumor, TMA and whole mount (WM) sections were scanned via an Aperio ScanScope imaging platform (Aperio Technologies, Vista, CA) with trainable GENIE morphometric software that permitsmorphometric quantification of scanned images.<br>For survival analysis, Kaplan-Meier survival plots were generated using the survival package in R.<br>For TCGA data analysis, we obtained TCGA level-3 data from TCGA data portal (https://tcgadata.nci.nih.gov). Box plot was drawn by boxplot in R.<br>To determine the linear relationship between LRIG1, AR and ERBB family members, we calculated Pearson correlation coefficient for linear regression by lm function and drew the scatter plot with regression line by plot and abline in R.<br>TCPA analysis was performed through the website (tcpaportal.org) developed by the Department of Systems Biology and Bioinformatics and Computational Biology at The University of Texas M.D Anderson Cancer Center.

For manuscripts utilizing custom algorithms or software that are central to the research but not yet described in published literature, software must be made available to editors/reviewers. We strongly encourage code deposition in a community repository (e.g. GitHub). See the Nature Research guidelines for submitting code & software for further information.

Data

Policy information about availability of data

All manuscripts must include a data availability statement. This statement should provide the following information, where applicable:

- Accession codes, unique identifiers, or web links for publicly available datasets
- A list of figures that have associated raw data
- A description of any restrictions on data availability

The authors confirm that the data supporting the findings of this study are available within the article and its supplementary materials, and are available from the corresponding author, upon request.

Field-specific reporting

Please select the one below that is the best fit for your research. If you are not sure, read the appropriate sections before making your selection.

☒ Life sciences ☐ Behavioural & social sciences ☐ Ecological, evolutionary & environmental sciences

For a reference copy of the document with all sections, see nature.com/documents/nr-reporting-summary-flat.pdf

Life sciences study design

All studies must disclose on these points even when the disclosure is negative.

Sample size	As described in the Methods, for all in vitro studies such as clonal and clonogenic assays, 3-4 technical replicates are generally used per condition and each individual experiment repeated 2-3 times (i.e., biological replicates). For in vivo studies, depending on the model system used, in general 8-20 mice/condition was used.
Data exclusions	In most cases we do not exclude any data points for analysis. Occasionally, for xenograft based tumor assays, it was clear that there existed 1 or 2 'outlier' responder(s) based on the tumor sizes and in these cases, the outliers in BOTH experimental and control groups were removed the same way and the remainder data was then used in statistical treatments.
Replication	As indicated in Methods (and sometimes, in Figures and legends), all studies were replicated 2-4 times. In vivo experiments were replicated in repeat experiments that collected data from several models and/or from multiple animals in each model.
Randomization	In our xenograft studies, immuno-deficient mice were implanted with human PCa cells expressing LRIG1 overexpressing/knockdown or the control construct and we measured the tumor growth kinetics and endpoint tumor weights. Genetic mouse tumor lines were pre-genotyped. In therapeutic studies, animals were randomly separated before treated with DOX.
Blinding	It is our standard laboratory practice to blind experiments as much as possible. Applicable to this project, the first author, Dr. Qiuhui Li, and the co-corresponding author, Dr. Xin Chen, work closely as a team for most of the experiments. For instance, for in vitro as well as in vivo studies, they design the experiments together but one will be the scientist to set up the experiments while the other will be the one to score and determine the results in a blind fashion. Then they come back to analyze and interpret data/results together.

Reporting for specific materials, systems and methods

We require information from authors about some types of materials, experimental systems and methods used in many studies. Here, indicate whether each material, system or method listed is relevant to your study. If you are not sure if a list item applies to your research, read the appropriate section before selecting a response.

Materials & experimental systems

n/a	Involved in the study	
☐	☒	Antibodies
☐	☒	Eukaryotic cell lines
☒	☐	Palaeontology
☐	☒	Animals and other organisms
☒	☐	Human research participants
☒	☐	Clinical data

Methods

n/a	Involved in the study	
☐	☒	ChIP-seq
☒	☐	Flow cytometry
☒	☐	MRI-based neuroimaging

Antibodies

Antibodies used	β-Actin Mouse mAb Santa Cruz SC-47778 (C-4)<br>AMACR Rabbit mAb Dako M3616 P504s<br>AKT Rabbit mAb Cell signaling 4691 C67E7<br>AR Mouse mAb Santa Cruz SC-7305 441

	AR Rabbit pAb Santa Cruz SC-816 N-20<br>AR Rabbit mAb Abcam Ab74272<br>BCL-2 Mouse mAb BD 610538<br>BrdU Mouse mAb Sigma B2531<br>c-MYC Rabbit mAb Abcam Ab32072 Y69<br>c-MYC Mouse mAb Sigma M4439 9E10<br>Caspase-3 Rabbit pAb R&D AF835<br>Cleaved LAMIN A/C Rabbit pAb Cell Signaling 2035<br>EGFR XP Rabbit mAb Cell signaling 4267 D38B1<br>EGFR Rabbit pAb Sigma HPA018530 C225<br>ERBB2 XP Rabbit mAb Cell signaling 4290<br>ERBB2 Rabbit pAb Santa Cruz SC-284 C-18<br>ERBB3 Mouse mAb Millpore 05-390<br>ERBB3 Rabbit pAb Santa Cruz SC-285 C-17<br>Flag Mouse mAb Sigma F1804<br>FKBP5 Rabbit pAb Cell Signaling 8245 FL-335<br>GAPDH Rabbit pAb Santa Cruz SC-25778<br>H3 (K27acetyl) Rabbit mAb Abcam Ab4729<br>Ki67 Rabbit pAb Leica Biosystems NCL-Ki67p Clone MM1<br>Ki67 Rabbit pAb Novus Biological NB110-89717<br>LAMIN A/C Rabbit pAb Santa Cruz SC-20681 H-110<br>LRIG1 Mouse mAb Sigma SAB4200445<br>LRIG1 Rabbit pAb Abcam Ab30707<br>LRIG1 Rabbit pAb Cell Signaling 12752<br>LRIG1 Sheep pAb R&D AF7498 aa 35-779<br>LRIG1 Goat pAB R&D AF3688 aa 37-794<br>LRIG1 Goat pAb Santa Cruz SC-50075 G-20 aa 620-670<br>LRIG1 Goat pAb Santa Cruz SC-50076 P16<br>p44/42 MAPK(ERK1/2) Rabbit mAb Cell signaling 4695 (137F5)<br>PCNA Mouse mAb Abcam ab29 PC10<br>pERK1/2 Rabbit pAb Cell signaling 9101 (Thr202/Tyr204)<br>pAKT Rabbit mAb Cell signaling 4060 (SER473)D9E<br>pEGFR Rabbit mAb Abcam Ab40815 (Y1092)[EP774Y]<br>pERBB2 Rabbit mAb Cell signaling 2243 (Tyr1221/1222) 6B12<br>pERBB2 Rabbit Ab Cell signaling 2247 (Tyr1248)<br>pERBB3 Rabbit mAb Cell signaling 4791 (Tyr1289) 21D3<br>pERBB3 Rabbit mAb Cell signaling 2842 (Tyr1289) D1B5<br>PSA Goat pAb Santa Cruz SC-7638 C-19
Validation	Data provided in the manuscript.

Eukaryotic cell lines

Policy information about cell lines

Cell line source(s)	Du145, PC3, PPC-1, LNCaP, VCaP, RWPE-1, and 22Rv1 PCa cells were obtained from ATCC.<br>IGR-1 (i.e., IGR-CaP1) cells were initially originated from a primary epithelial prostate cancer. LAPC9 and LAPC4 xenograft lines were initially provided by Dr. Robert Reiter (UCLA).
Authentication	All cell lines authenticated by institutional CCSG Cell Line Characterization Core via short tandem repeat (STR) analysis.
Mycoplasma contamination	All cell lines were regularly tested to be negative for mycoplasma contamination using the Agilent MycoSensor PCR Assay kit.
Commonly misidentified lines<br>(See ICLAC register)	*Name any commonly misidentified cell lines used in the study and provide a rationale for their use.*

Animals and other organisms

Policy information about studies involving animals; ARRIVE guidelines recommended for reporting animal research

Laboratory animals	The study involved male immunodeficient NOD/SCID and NOD/SCID-IL2Rγ-/- (i.e., NSG) mice aged from 2weeks to 9 month.<br>Male FVB-TRAMP and FVB-C-MYC mice aged from 2weeks to 9 month are involved in this study.
Wild animals	The study did not involve wild animals.
Field-collected samples	The study did not involve samples collected from the field.
Ethics oversight	All animal related (xenograft and genetic model) studies have been approved by the M.D Anderson Cancer Center (MDACC) IACUC (ACUF#00000923-RN00) or the Roswell Park Comprehensive Cancer Center (RPCCC) (animal protocol# 1331M).

Note that full information on the approval of the study protocol must also be provided in the manuscript.

ChIP-seq

Data deposition

☐ Confirm that both raw and final processed data have been deposited in a public database such as GEO.

☐ Confirm that you have deposited or provided access to graph files (e.g. BED files) for the called peaks.

Data access links<br>*May remain private before publication.*	*For "Initial submission" or "Revised version" documents, provide reviewer access links. For your "Final submission" document, provide a link to the deposited data.*
Files in database submission	*Provide a list of all files available in the database submission.*
Genome browser session<br>(e.g. UCSC)	*Provide a link to an anonymized genome browser session for "Initial submission" and "Revised version" documents only, to enable peer review. Write "no longer applicable" for "Final submission" documents.*

Methodology

Replicates	*Describe the experimental replicates, specifying number, type and replicate agreement.*
Sequencing depth	*Describe the sequencing depth for each experiment, providing the total number of reads, uniquely mapped reads, length of reads and whether they were paired- or single-end.*
Antibodies	*Describe the antibodies used for the ChIP-seq experiments; as applicable, provide supplier name, catalog number, clone name, and lot number.*
Peak calling parameters	*Specify the command line program and parameters used for read mapping and peak calling, including the ChIP, control and index files used.*
Data quality	*Describe the methods used to ensure data quality in full detail, including how many peaks are at FDR 5% and above 5-fold enrichment.*
Software	*Describe the software used to collect and analyze the ChIP-seq data. For custom code that has been deposited into a community repository, provide accession details.*
